# New improvements in grapevine genome editing: high efficiency biallelic homozygous knock-out from regenerated plantlets by using an optimized zCas9i

**DOI:** 10.1186/s13007-024-01173-8

**Published:** 2024-03-18

**Authors:** Jérémy Villette, Fatma Lecourieux, Eliot Bastiancig, Marie-Claire Héloir, Benoit Poinssot

**Affiliations:** 1 Agroécologie, INRAE, Institut Agro, Université de Bourgogne, Dijon, France; 2 UMR1287 EGFV, CNRS, Université de Bordeaux, INRAE, Bordeaux Sciences Agro, ISVV, Villenave d’Ornon, Dijon, France

**Keywords:** *Vitis vinifera*, CRISPR/cas9, Efficient gene editing, zCas9i

## Abstract

**Background:**

For ten years, CRISPR/cas9 system has become a very useful tool for obtaining site-specific mutations on targeted genes in many plant organisms. This technology opens up a wide range of possibilities for improved plant breeding in the future. In plants, the CRISPR/Cas9 system is mostly used through stable transformation with constructs that allow for the expression of the *Cas9* gene and sgRNA. Numerous studies have shown that site-specific mutation efficiency can vary greatly between different plant species due to factors such as plant transformation efficiency, Cas9 expression, Cas9 nucleotide sequence, the addition of intronic sequences, and many other parameters. Since 2016, when the first edited grapevine was created, the number of studies using functional genomic approaches in grapevine has remained low due to difficulties with plant transformation and gene editing efficiency. In this study, we optimized the process to obtain site-specific mutations and generate knock-out mutants of grapevine (*Vitis vinifera* cv. ‘Chardonnay’). Building on existing methods of grapevine transformation, we improved the method for selecting transformed plants at chosen steps of the developing process using fluorescence microscopy.

**Results:**

By comparison of two different *Cas9* gene and two different promoters, we increased site-specific mutation efficiency using a maize-codon optimized Cas9 containing 13 introns (zCas9i), achieving up to 100% biallelic mutation in grapevine plantlets cv. ‘Chardonnay’. These results are directly correlated with Cas9 expression level.

**Conclusions:**

Taken together, our results highlight a complete methodology for obtaining a wide range of homozygous knock-out mutants for functional genomic studies and future breeding programs in grapevine.

**Supplementary Information:**

The online version contains supplementary material available at 10.1186/s13007-024-01173-8.

## Background

One of the major concerns in viticulture is the adaptation of grapevine (*Vitis vinifera*) to biotic and abiotic stresses. Traditional breeding approaches are currently the only way to obtain new resistant cultivars against pathogens in a context of reducing chemical inputs and adapted to climate change. However, this method is extremely time-consuming and requires a lot of space and resources. As a result, quickly obtaining resistant cultivars against future pests remains a significant challenge [1]. New genome editing technologies could provide an alternative to accelerate the process of obtaining resistant cultivars. Genome editing using site-specific nucleases can induce targeted mutations to improve or suppress a function in many organisms. Genome editing technologies include three approaches: transcription activator-like effector nucleases (TALENs), zinc-finger nucleases (ZFNs), or clustered regularly interspaced short palindromic repeats/CRISPR-associated protein 9 system (CRISPR/Cas9). Among them, CRISPR/cas9 is highly efficient, the most cost-effective, the least time-consuming and consequently the most commonly used in many plant and animal species [2, 3].

Indeed, the discovery of CRISPR/cas9 in 2012 opened up new perspectives for functional genomic studies in many plant species with strong agronomic interest, including rice [4], wheat [5], maize [6], apple, pear [7], tomato [8], strawberry [9] and grapevine [10]. In plants, targeted mutations are mostly realized *via Agrobacterium*-mediated transformation to ultimately obtain stable transformed organisms. *Agrobacterium* contains a plasmid construct with an antibiotic resistance gene, the *Cas9* gene, and one or several sgRNA in multiplex to target one or several genes. The sgRNA multiplex consists of a spacer (tRNA), a complementary sequence of 20 nucleotides to specifically target a gene, and a scaffold that forms a stabilized complex with Cas9. The expression of sgRNA multiplex is most often controlled by the commonly used U6-26 promoter from *A. thaliana* [11]. After transformation, plant cells can express these different components to induce site-specific cleavage on genomic DNA and mediate site-specific mutations [12].

In grapevine, the first study using efficient CRISPR/cas9 appeared in 2016 on cv. ‘Chardonnay’ [10, 13]. With the availability of the whole-genome sequence, many research groups have developed CRISPR/cas9-based functional genomic projects to decipher the function of some genes of interest in grapevine [14]. For example, *VvCCD8* was shown to affect shoot architecture in grapevine rootstock ‘41B’, *IdnDH* gene have been described to be involved in tartaric acid synthesis in grapevine cv. ‘Chardonnay’ and *VvPR4b* gene was characterized to play an important role in downy mildew resistance of grapevine ‘Thompson seedless’ [10, 15, 16]. CRISPR/Cas9 technology also fits perfectly with a strategy of obtaining knock-out mutants for susceptibility (*S*) gene. Inactivation of *S* gene confers a broad and durable resistance of plants [17, 18]. In grapevine, edited *VvWRKY52* [19] and *VvMLO3* [20] conferred resistance to *Botrytis cinerea* and *Erysiphe necator*, respectively. Recently, the two *S* genes *VvDMR6s*, encoding enzymes involved in the salicylic acid catabolism, were described as decreasing susceptibility against downy-mildew in two grapevine varieties [21].

Even though some studies have shown site-specific mutated plants using CRISPR/cas9 obtained with a repeatable transformation process using *A. tumefaciens*, selection process including plant regeneration and genome editing on grapevine remains time-consuming and relatively low efficient compared to *Arabidopsis thaliana* or *Oryza sativa* [22–24]. Globally, the number of target edited genes remains relatively low in grapevine. Ren et al. [25]. reported on various functional genomics studies using knock-out mutants with varying percentages of genome editing efficiency. These different percentages obtained at the embryogenic cell stage seem to reflect high variability between studies and varieties used [25]. Thus, a stronger and more repeatable target gene efficiency could pave the way for more functional genomic studies on grapevine. In different species, recent studies have shown that Cas9 efficiency depends on many parameters such as sgRNA design, promoters and terminators used for Cas9 expression (as already shown in grapevine with endogenous *UBQ* promoter) and the presence of one or two NLS domains, with better efficiency being achieved with two NLS domains to target the nuclease to the nucleus [23, 26, 27]. However, the most important parameter to consider for achieving a better efficiency is the nucleotide sequence of the *Cas9* gene [28].

In this work, we optimized three distinct parameters in the process of obtaining a high level of site-specific mutated grapevine plants using CRISPR/Cas9. Firstly, we investigated a highly efficient screening step using antibiotic selection coupled with the fluorescent DsRed2 selection marker at different stages of development (embryogenic cells, embryos, and plantlets) to decrease chimeric effects [29–31]. Secondly, we considered the importance of nucleotide sequence of two *Cas9* genes. We used either human codon-optimized Cas9 (hCas9) which seems more efficient than dicotyledonous plant codon-optimized Cas9 [32] or a maize codon-optimized Cas9 (zCas9i) constituted of an optimized intron-containing sequence with 13 introns. The zCas9i successfully improved genome editing efficiency in various plant species including woody plants [23, 33–35]. Thirdly, we compared two independent promoters for *Cas9* expression. Knowing that the *RPS5a* promoter has been reported to be one of the most appropriate for controlling *Cas9* gene expression in *Arabidopsis thaliana* [28, 36], we compared genome editing efficiency between the *35S* and *RPS5a* promoters controlling the *zCas9i* expression in this study. To further compare genome editing efficiency between two different codon-optimized Cas9 and the use of two different promoters, transcription and translation levels of Cas9 for each construct were quantified.

We report in this study an optimized efficiency of targeted site-specific mutations using the codon-optimized for *Zea mays*, *zCas9i* in *Vitis vinifera*. We obtained homozygous knock-out mutants within 9 months using ‘Chardonnay’ cultivar. This work could contribute to enhance the use of New Breeding Techniques (NBT) for grapevine breeding programs.

## Results

### CRISPR/cas9 expression vectors using two codon-optimized Cas9 with two different promoters

**Fig. 1:**
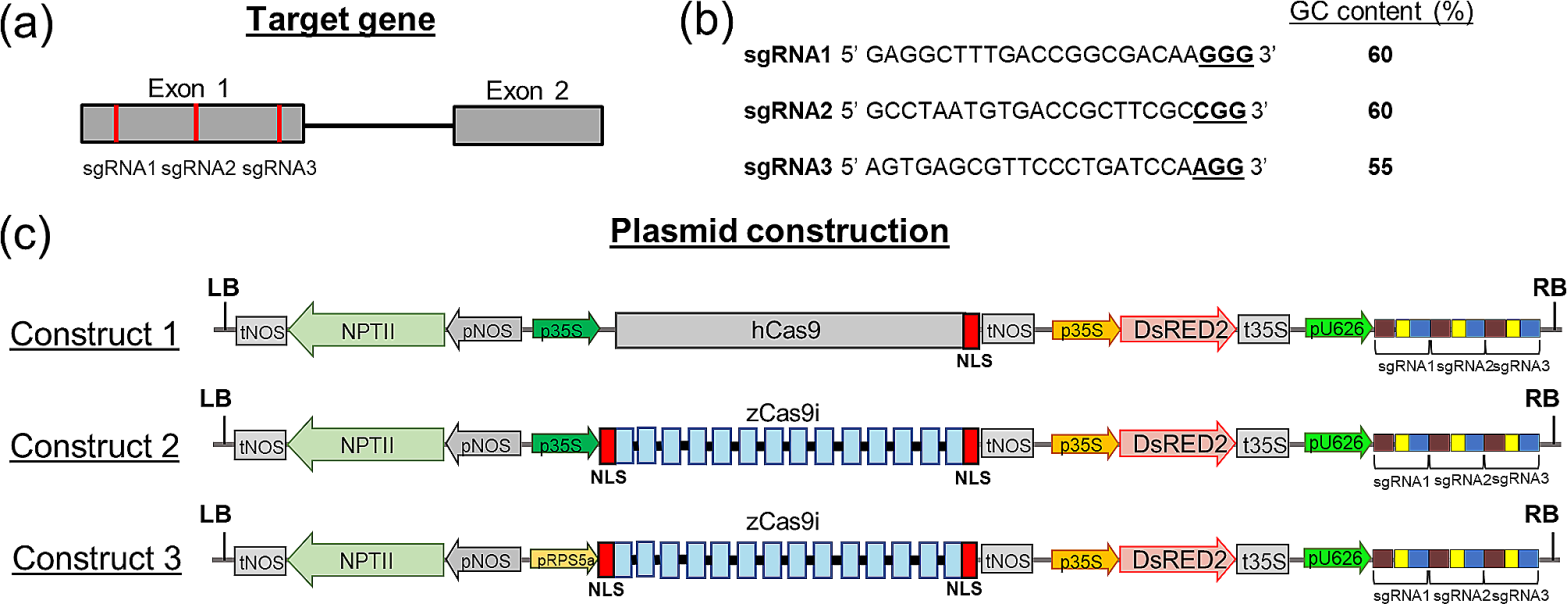
Schematic illustration of target gene and different plasmid constructions. (**a**) Illustration of the target gene belonging to LysM receptor-like kinase family (Vitvi05g00623) with the location in red of the sgRNA1, 2 and 3 in exon1. (**b**) Detailed sgRNA sequences with the Protospacer Adjacent Motif (PAM) underlined and the GC content. (**c**) Plasmid construction for grapevine embryogenic cells transformation. Both constructions are constituted of one cassette with *NPTII* gene under the control of *NOS* promoter for antibiotic selection, one cassette expressing the *DsRed2* gene under the control of *35S* promoter for fluorescent selection and a multiplex of 3 sgRNAs under the control of *U6-26* promoter from *A. thaliana*. Constructions differ by the nucleotide sequence encoding *Cas9* gene with integration of 13 introns for zCas9i and by the use of two promoters controlling *Cas9* gene expression

To compare genome editing efficiency, our study focused on one target gene belonging to the grapevine LysM receptor-like kinase family [37, 38]. The target gene consists of two exons. To obtain knock-out mutants for this gene, three distinct sgRNAs distributed in the first exon were chosen (Fig. 1a). The sequences of the 3 sgRNAs are shown with the NGG protospacer adjacent motif (PAM) underlined for adaptation to Cas9 recognition and binding (Fig. 1b). It can be noted that the first nucleotide in the PAM motif is different for each sgRNA and all sgRNAs show comparable GC content between 55% and 60% (Fig. 1b). Then, three Cas9 constructs were prepared to compare two codon-optimized Cas9 sequences and two promoters controlling *Cas9* expression (Fig. 1c). Each construct includes the same antibiotic resistant gene *NPTII* (kanamycin resistance), the *DsRed2* gene for fluorescent selection, and the sgRNA multiplex with the three sgRNA mentioned in Fig. 1a-b. Regarding the cassette for *Cas9* expression, construct 1 uses *hCas9* (human codon-optimized sequence) with one nuclear localization sequence (NLS) without intron under the control of the *35S* promoter (Fig. 1c). Construct 2 expresses *zCas9i* (codon-optimized for *Zea mays*, including 13 introns from *A. thaliana* genome) [23] and two NLS domain under the control of the *35S* promoter whereas construct 3 uses the *A. thaliana RPS5a* promoter to express *zCas9i* with two NLS domains (Fig. 1c). The *RPS5a* promoter has been reported to be among the most efficient in driving *Cas9* expression in *A. thaliana* [28, 36], a dicotyledonous plant species such as *V. vinifera*. These three constructs were used to compare two parameters related to Cas9 expression and translation: the optimization of codon usage on the *hCas9* gene compared with *zCas9i* which contains intronic sequences, and the effect of two different promoters (*p35S* and *pRPS5a*) on *zCas9i* expression.

### Transformation of embryonic cells and selection of stable transformed plantlets

**Fig. 2:**
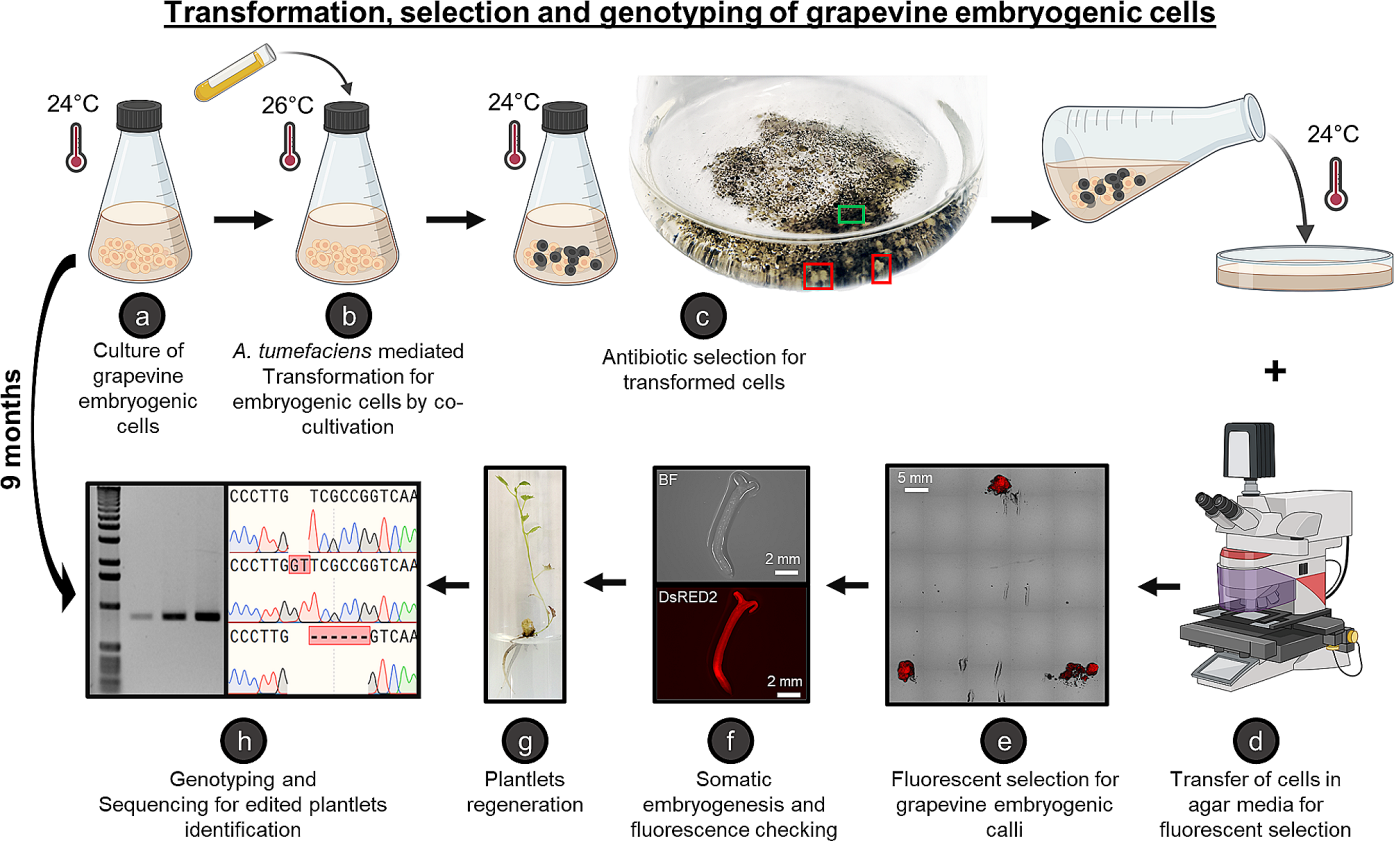
Detailed process to obtain transformed grapevine embryogenic cells (cv. Chardonnay) and regenerated plantlets. Antibiotics selection for transformed cells allows to discern resistant white embryogenic cells (c, red outlined) and non-resistant black embryogenic cells (c, green outlined). (**a**-**h**) Indicate the different steps, directly described in this figure, of the plant method developped to obtain high frequency biallelic homozygous knock-out grapevine plantlets. BF: Brightfield. Figure created with biorender.com

To compare the effectiveness of these three constructs in editing the selected gene, we transformed grapevine embryogenic cells of *V. vinifera* cv. ‘Chardonnay’ [22] and used DsRed2 fluorescence to mitigate the risk of obtaining plantlets with chimeric phenotypes at a macroscopic scale. The transformation was performed by co-cultivation at 26 °C, and using the available *V. vinifera* cv. ‘Chardonnay’ embryogenic cell culture and the *Agrobacterium tumefaciens* EHA105 harbouring the CRISPR/Cas9 expression vector (Fig. 2a-b). Then, co-cultivated cells were submitted to antibiotic selection. Two months of antibiotic selection were needed to obtain white and black micro calli corresponding to resistant (red outlined) and non-resistant (green outlined) embryogenic cells, respectively (Fig. 2c). At this step, fluorescence observations on white calli are performed to isolate homogenously transformed embryogenic calli and select fully transformed embryos that present potentially mutations (Fig. 2d). Somatic embryogenesis is then initiated for calli showing homogeneous fluorescence (Fig. 2e and f). Mature embryos are collected and placed in a medium promoting regeneration of plantlets (Fig. 2g). It is important to note that fluorescence was verified at some steps of development (callus, embryo and plantlet). The last observation of the transformed plantlets in fluorescence allowed the exclusion of those expressing DsRed2 in a heterogeneous way. Only plantlets uniformly displaying fluorescence are genotyped and assayed for targeted gene editing (Fig. 2h).

**Fig. 3:**
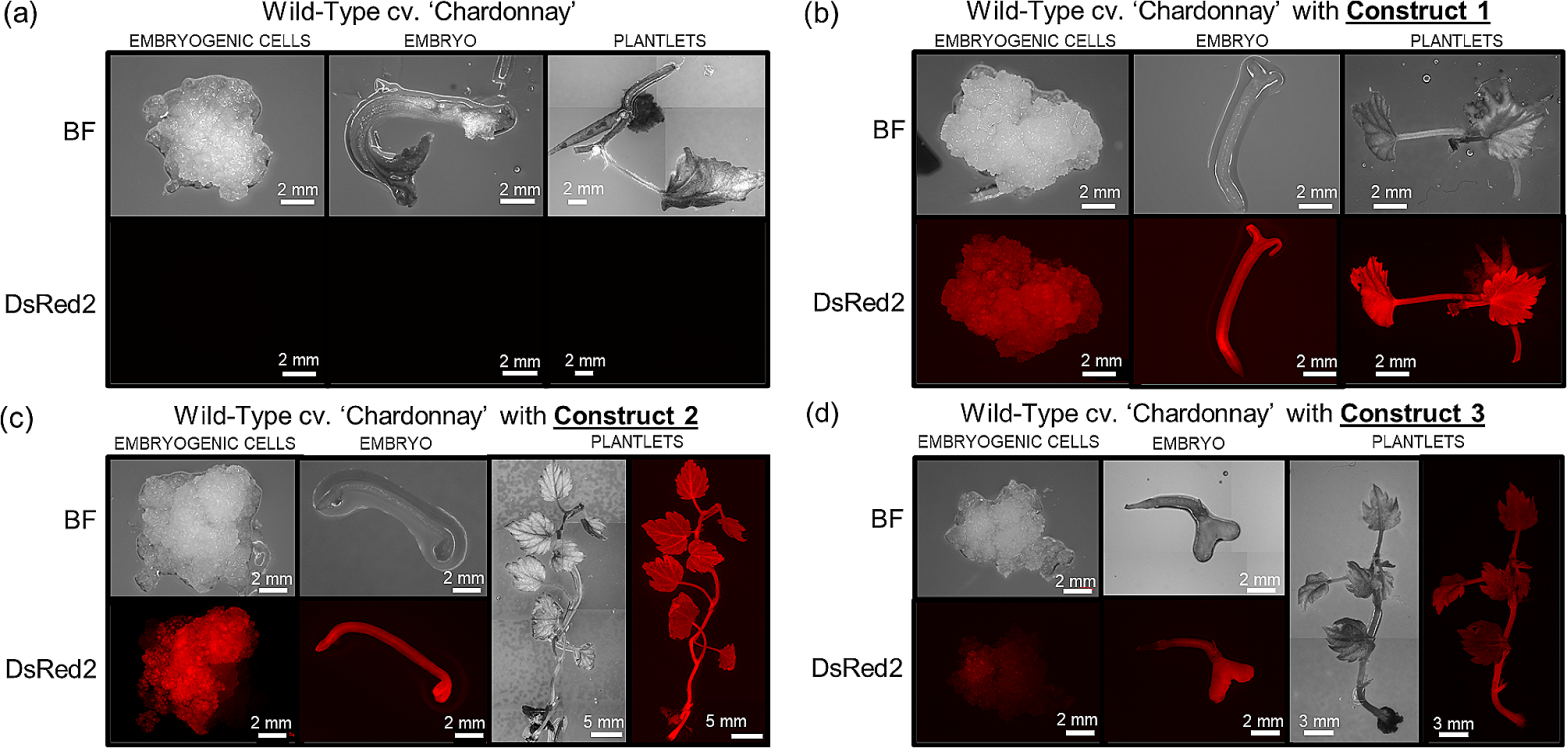
DsRed2 fluorescence emitted by transformed grapevine embryogenic cells, embryo and plantlets for transgenic lines containing each construction or wild-type. Embryogenic cells, embryo and plantlets growing on agar plate containing adapted media, were observed 3, 5 and 8 months after transformation, respectively. Brightfield (BF) and fluorescent (DsRed2) pictures of each stage are showed. (**a**) Non-transformed Wild-Type cv. ‘Chardonnay’ as a control of autofluorescence. (**b**) Wild-Type transformed with construct 1 (*p35S::hCas9*). (**c**) Wild-Type transformed with construct 2 (*p35S::zCas9i*). (**d**) Wild-Type transformed with construct 3 (*pRPS5a::zCas9i*)

Figure 3 presents one individual of each transformation, from the embryogenic cell stage to the plantlet stage and compared to Wild-Type (WT). No fluorescence was observed in WT embryogenic cells, embryos and plantlets. It is important to note that even in the presence of chlorophyll known to emit autofluorescence, WT grapevine plantlets do not emit fluorescence at the excitation wavelength (561 nm) used for DsRed2 (Fig. 3a) [39, 40]. Indeed, the emission wavelength of DsRed2 is around 587 nm whereas chlorophyll emission wavelength is known to be around 680 nm. Comparing the three constructs used, all of them are able to express DsRed2 at different development stages (Fig. 3) and the regenerated plantlets are homogenously transformed, without any apparent chimerism on a macroscopic scale. The use of fluorescence has two main objectives: firstly, we can accurately screen transformed cells very early in the selection process; secondly, we can decrease the risk of chimerism in our regenerated plantlets which is a recurrent problem when plantlets are regenerated from embryogenic cells (Fig. 3b, c, d) [41, 42].

### Analysis of CRISPR/cas9-mediated gene editing efficiency

Despite the reduction in chimerism with DsRed2 using during transformation and selection protocol, target gene editing can also lead to different types of mutation within the same individual. This aspect of chimerism can be reduced by optimizing the efficiency of genome editing. Consequently, to optimize CRISPR/cas9 efficacy on grapevine, we focused on parameters controlling *Cas9* expression (adding 13 intronic sequences from *A. thaliana* and using two different promoters) and Cas9 translation (codon optimized for the genome of human or maize).

**Fig. 4:**
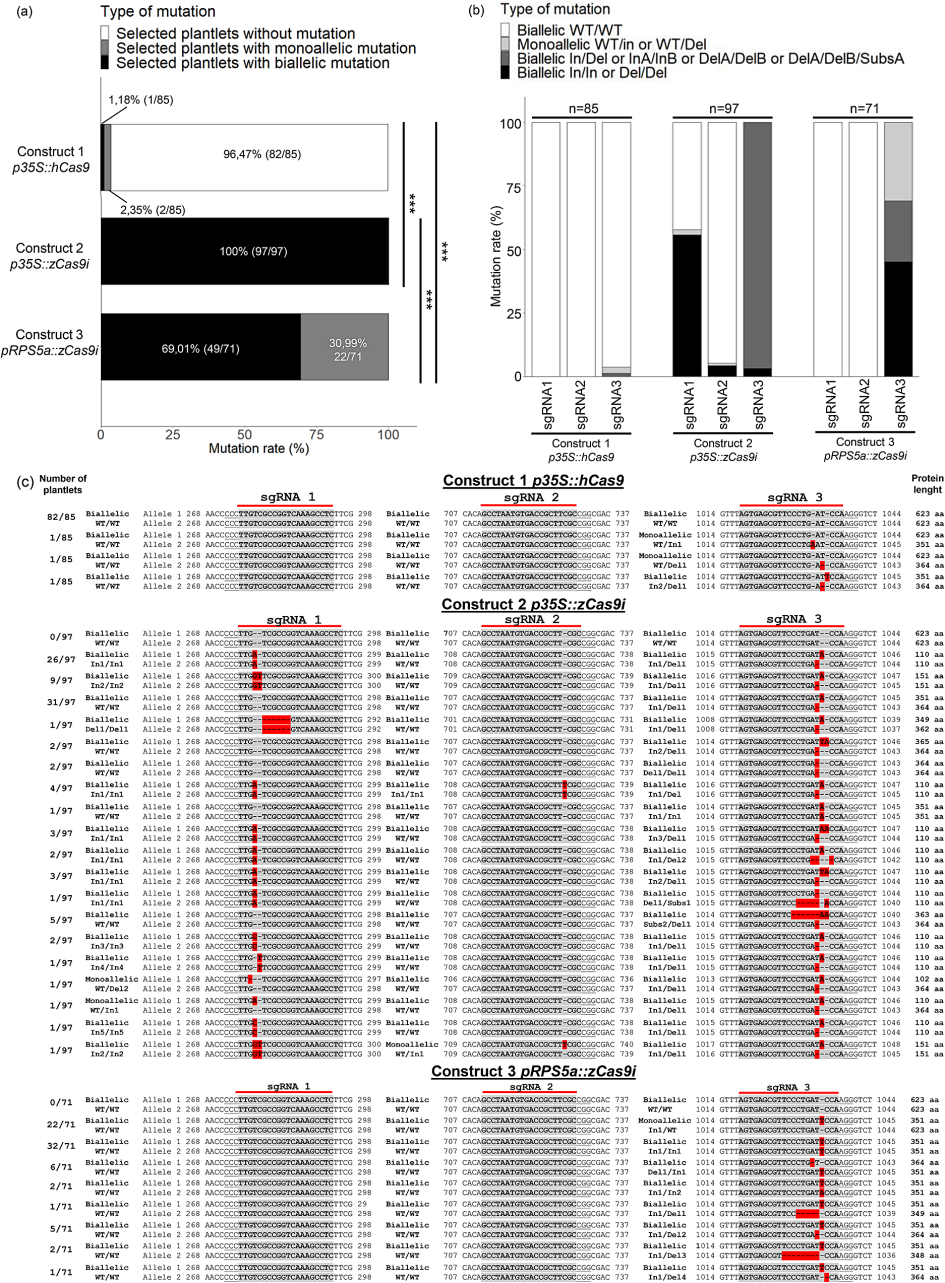
Comparison of target edited gene mutation efficiency for transgenic lines of ‘Chardonnay’ expressing each construction. (**a**). Efficiency of target edited gene for each construction considering all sgRNAs. Types of mutation are divided in three groups (selected plantlets without mutation in white, selected plantlets with monoallelic mutation in grey, selected plantlets with biallelic mutation in black). All plantlets previously selected by *Cas9* genotyping allowed to amplify a PCR product corresponding to the target gene. PCR products were then sequenced and aligned to identify mutation rate. 85, 97 and 71 plantlets were selected for construct 1, 2 and 3, respectively (Pairwise comparison of proportions; ***, *P* < 0.001). (**b**). Comparison of target edited mutation rate for each sgRNA and each construction. All sequences analyzed in Fig. 3a have been ordered according to the nature of the mutation. 4 types of mutation are considered: biallelic WT/WT in white, monoallelic WT/in or WT/Del in light grey, biallelic In/Del or InA/InB or DelA/DelB or DelA/DelB/SubsA in dark grey (where A and B are random mutation), Biallelic In/In or Del/Del in black. In = insertion of one or several nucleotides, Del = Deletion of one or several nucleotides, subs = substitution. (**c**). Alignment of the different edited sequences against wild-type sequence for the three sgRNAs of each construction. Wild-type sequence is highlighted in grey and all insertions or deletions are highlighted in red. Two alleles are represented for each plantlet to highlight monoallelic and biallelic mutations. The protein length and the ratio of plantlets showing the corresponding sequence are mentioned on the right and on the left, respectively

We compared the level of the targeted gene edition with two different nucleotide sequences of nucleases (*hCas9* or *zCas9i*) and two different promoters (*p35S* or *pRPS5a*, Fig. 1b). We obtained 85, 97 and 71 plantlets from at least four independent red-selected callus embryogenic cells transformed with the construct 1, 2 or 3, respectively. The first step was to verify the presence of T-DNA insertion by amplifying a part of the *Cas9* gene for each plantlet regenerated. We firstly checked the specificity of the primers designed for *hCas9* and *zCas9i* genotyping and observed no amplification for WT plantlets (Fig. S1). Results from amplification of a part of the *Cas9* gene showed that all 85 plantlets from transformed embryogenic cells with construct 1 contained the T-DNA insertion (Fig. S2a). Similarly, 97 and 71 plantlets transformed with the construct 2 or 3, respectively, also exhibited 100% of plantlets containing T-DNA insertions (Fig. S3a, S4a). After checking that all plantlets could express the *Cas9* gene, we amplified the region of the target gene containing the three sgRNAs to determine the gene editing efficiency by sequencing (Fig. S2b, S3b and S4b). All sequenced PCR products were aligned against WT sequence and direct-site edits were identified on both alleles for all plantlets expressing construct 1 (Fig. S5), 2 (Fig. S6) or 3 (Fig. S7). Firstly, if we consider all mutations on the target gene, we observed that 100% of selected plantlets transformed with construct 2 are edited with biallelic mutations resulting in a predicted truncated protein (Fig. 4a, c). By comparison, only one out of the 85 selected plantlets expressing construct 1 had a biallelic mutation, proving a significant difference in gene editing efficiency between transgenic plantlets expressing construct 1 or 2. Then, analysis of mutations reflecting target gene editing efficiency showed that 69% of plantlets expressing construct 3 had biallelic mutations. Construct 3 showed a relatively important mutation rate for biallelic mutations, significantly higher compared to construct 1 but significantly lower compared to construct 2 (Fig. 4a). To go deeper into mutation rate analysis, we organized all gene editing events into four groups of mutations according to sgRNA for each construct: biallelic WT/WT, monoallelic WT/In or WT/Del, biallelic In/Del or InA/InB or DelA/DelB or DelA/DelB/SubsA and biallelic In/In or Del/Del (Fig. 4). We chose this classification due to the presence of many heterozygous and biallelic mutations. For construct 1 using hCas9, we did not observe any mutation on sgRNA1 and sgRNA2. Concerning sgRNA3, only one plantlet out of 85 was edited through a biallelic mutation leading to a KO mutant. We can consider that regardless of sgRNA, editing of the target gene is ineffective on grapevine cv ‘Chardonnay’ using construct 1. Overall, construct 2 showed a higher level of target gene editing. Indeed, for sgRNA1, we observed that 56% of plantlets had biallelic insertion corresponding to homozygous mutation on two alleles (Fig. 4b, c). With the first sgRNA1, more than half of the 97 selected plantlets had a truncated predicted protein of either 110 or 151 amino acids whereas the length of the control WT protein is 623 amino acids (Fig. 4c). The efficiency of target gene editing with sgRNA2 for construct 2 showed an efficiency of 4% for biallelic insertion and a 1% percent for monoallelic WT/In mutation which is very low compared to the sgRNA1 (Fig. 4b, c). By contrast, the third sgRNA3 triggered 100% edition of the target gene. Among them, 3% were biallelic homozygous In/In or Del/Del mutations and 97% were biallelic mutations of either In/Del, InA/InB, DelA/DelB or DelA/DelB/SubsA, as detailed in Fig. 4c. Even if we observed different type of mutations on the two alleles for the same plantlet, these mutations lead to truncated proteins with lengths between 351 and 364 amino acids (Fig. 4c). To conclude, transgenic lines expressing construct 2 showed a higher editing efficiency for the sgRNA3 compared to the two other sgRNAs. However, the use of zCas9i considerably improved the efficiency of target gene editing, with 100% of the 97 selected plantlets showing 19 types of mutation. Plantlets transformed with construct 3 using *zCas9i* with the *RPS5a* promoter from *A. thaliana* did not show any mutation for sgRNA1 and sgRNA2. Nevertheless, for sgRNA3, we observed 45% of biallelic homozygous insertions and 24% biallelic In/Del and InA/InB mutations leading to KO mutants. Finally, the rest of the plantlets showed 31% monoallelic WT/In mutations. To conclude on construct 3, we observed high efficiency of target gene editing with various kinds of mutations only for sgRNA3 (Fig. 4b, c). Overall, we observed a higher frequency of gene editing events using sgRNA3 when compared to other sgRNAs, displaying different kinds of deletions (between 1 and 6 nucleotides) and different types of insertions (1 or 2 nucleotides). Notably, sgRNA1 used with construct 2 led to insertions of 1 or 2 nucleotides and deletions of up to 6 nucleotides at the targeted site. Surprisingly, despite the use of three sgRNA in the same exon of the target gene, we did not observe huge deletions of several hundred nucleotides as already reported in rice and Arabidopsis, suggesting a high capacity for DNA repair in grapevine (Fig. 4c) [23, 24]. When we looked at constructs expressing *zCas9i*, we observed a significant improvement in target gene editing compared to constructs expressing *hCas9*. These results suggest that the optimized sequence of zCas9i is better adapted to generate gene editing in grapevine, contrary to hCas9. To complete this study, we tried to understand which parameters influenced these significant differences in target gene editing by comparing transcripts accumulation and protein amounts according to the three constructs used.

### Cas9 transcript and protein accumulation depends on the different constructs used

**Fig. 5:**
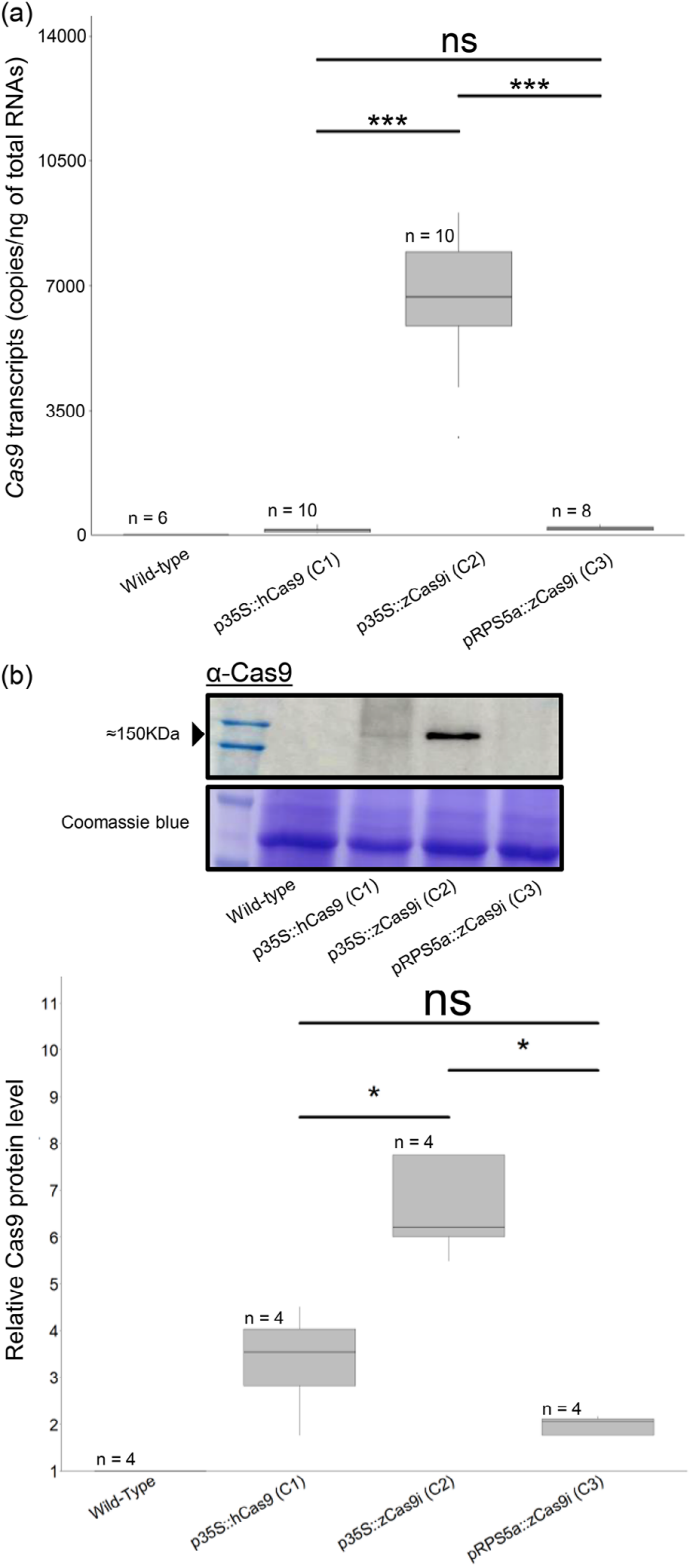
Cas9 transcripts and protein accumulation in stable transformed grapevine plantlets expressing each construction or in wild-type plantlets. (**a**) *Cas9* transcripts levels from leaves collected on one-month grapevine plantlets. Asterisks indicate a statistically significant difference between two constructions highlighted by a bar (Wilcoxon test; ***, *P* < 0,001; N.S., no significant). (**b**) Immunodetection of Cas9 accumulation by western blotting in grapevine plantlets upon the expression of different genes and promoters. Asterisks indicate a statistically significant difference between two constructions highlighted by a bar (Wilcoxon test; *, *P* < 0,05; N.S., no significant). For both experiments, “n” represents the number of biological replicates. Each independent biological replicate involved sampling three leaves from three distinct plantlets. Different plantlets were sampled for each independent biological replicate. All the plantlets sampled correspond to those sequenced in Fig. 4

To understand the effect of intron optimization and the use of different promoters on *zCas9i* expression, we quantified *hCas9* and *zCas9i* transcripts by RT-qPCR at the plantlet stage. All transgenic plantlets with each construct showed *Cas9* expression (Fig. 5a). Based on the means of biological replicates, construct 1, expressing the *hCas9* under the control of the *35S* promoter, showed an average expression level of 141 copies of *hCas9* transcripts per ng of total RNAs. Transgenic plantlets expressing construct 2, with a codon-optimized *zCas9i* under the control of the *35S* promoter, showed a statistically higher expression level compared to construct 1, with 7338 copies of *zCas9i* transcripts per ng of total RNAs. Finally, the transgenic plantlets expressing construct 3, with *zCas9i* under the control of the *RPS5a* promoter, showed an average expression level of 189 copies of *zCas9i* transcripts per ng of total RNAs, comparable to transgenic lines expressing construct 1 and statistically lower than plantlets transformed with construct 2 (Fig. 5a). When placed under the control of the same *35S* promoter, the accumulation of *Cas9* transcripts, assessed by RT-qPCR, shows that it is greater in the intron-optimized *Cas9* than in the intron-free one. Interestingly, the same *zCas9i* under the control of the *RPS5a* promoter showed a very low level of expression. This indicates that both the promoter used and the type of Cas9 are important for a high expression of the *Cas9* in ‘Chardonnay’ lines.

To further investigate the optimization of Cas9 efficiency on the basis of the construct used, we analysed protein accumulation by western blotting using a *Streptococcus thermophilus* Cas9-specific antibody. Interestingly, results were directly correlated with RT-qPCR results because the Cas9 protein amounts in transgenic plantlets expressing construct 2 were significantly higher compared to others constructs (Fig. 5b). In agreement with the expression level of the *Cas9* transcripts for transgenic lines expressing the construct 1 or 3, protein amounts remained very low by immunodetection, compared to plantlets expressing construct 2. Taken together, these results show better transcription with intron-optimized *zCas9i* and consequently more translated protein available to perform genome editing. Interestingly, despite low protein expression for transgenic lines expressing construct 3 (Fig. 5b), we observed a relatively high level of gene editing (Fig. 4a). Improvement in transcript and protein expression correlated with a high target gene editing efficiency for transgenic lines expressing construct 2 could also increase the probability of inducing mutations in off-target sequences.

### Off-target sequences analysis

**Fig. 6:**
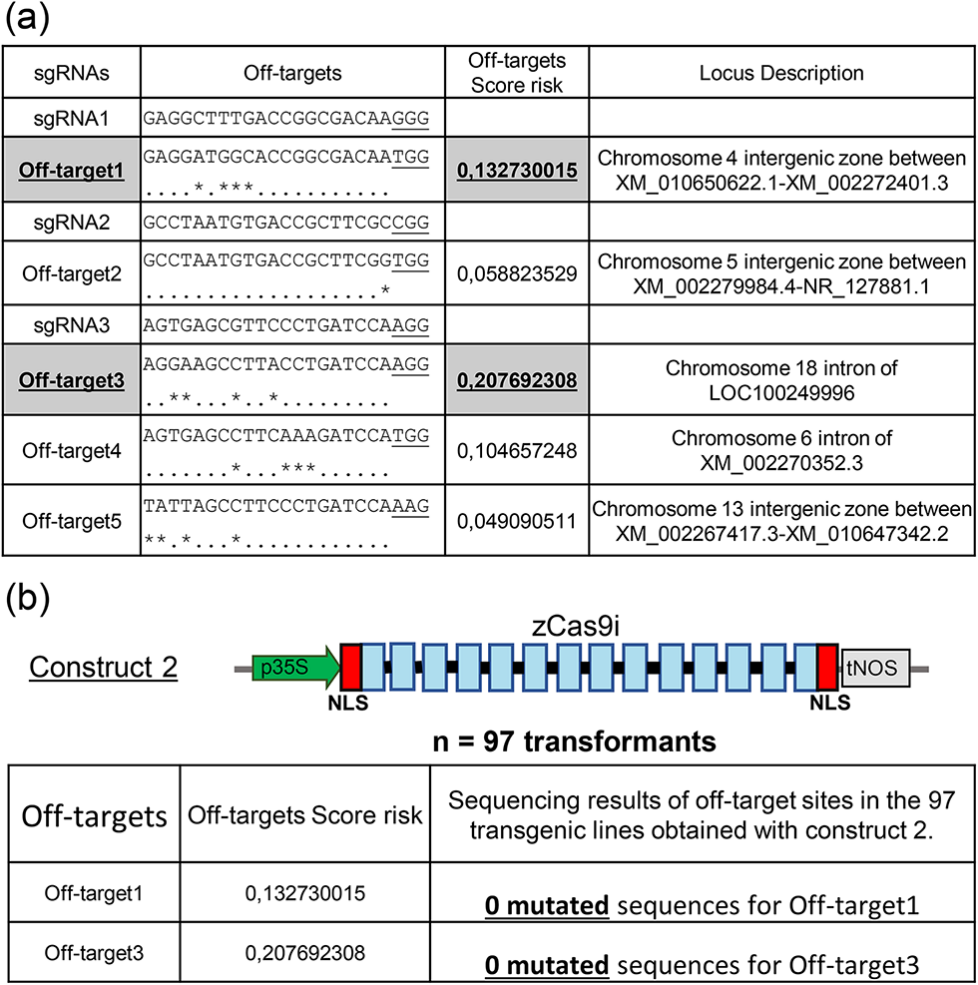
Off-target analysis in transgenic lines expressing construct 2. (**a**) Listing of predicted off-targets for both sgRNAs. One off-target is predicted for the sgRNA1 and sgRNA2 and 3 off-targets are predicted for sgRNA3. Sequence of different off-targets, the off-targets score risk and locus description are detailed in the second, third and fourth column respectively. “*” represent mismatch between sgRNA and off-target sequences. (**b**) Off-target 1 and 3 analysis on transgenic lines expressing construct 2. Off-target 1 and 3 have the most elevated score risk. Each off-target region has been amplified by PCR and sequenced. Sequencing results for each plantlet are presented in the third column

To determine potential off-target events, off-target prediction analyses were carried out using the CRISPOR database. Five putative off-target sites were identified for the three sgRNAs studied (Fig. 6a). sgRNA1 and sgRNA2 each had one predicted off-target site while sgRNA3 had three off-targets. Off-target prediction analyses were complemented by off-target risk scores, which reflect the probability of obtaining mutations for each off-target site. To analyse putative off-target events, we chose the two most probable off-targets: off-target 1 and off-target 3 with risk scores of 0.133 and 0.208, respectively (Fig. 6). The first is located in an intergenic region of chromosome 4 on the grapevine genome. The second off-target site is located on chromosome 18 in an intron between two exons of LOC100249996, which could impact the gene expression. To analyse the presence of putative off-targets, we focused on the most efficient transgenic lines expressing construct 2 (*p35S::zCas9i*) (Fig. 6b). PCR products corresponding to the DNA regions of off-target 1 and off-target 3 were sequenced and aligned to the corresponding WT sequence to identify potential mutations (Fig. S8, S9, S10). For both studied off-targets, no mutations were found in the 97 independent transgenic lines sequenced PCR products (Fig. 6b, S10). In conclusion, despite a high mutation efficiency and a strong accumulation of the Cas9 protein, the analysis of some putative off-target sequences shows that they were not impacted.

## Discussion

In this study, we obtained 100% biallelic mutations leading to knock-out grapevine plantlets for the target gene by using the combination of antibiotic and DsRed2 selection, and an intron-optimized z*Cas9i*. The prerequisite steps of transformation and antibiotic selection of embryogenic cells from *V. vinifera* cv. ‘Chardonnay’ were already described [22]. Moreover, the using of reporter genes as *GFP* (Green Fluorescent Protein) for selecting positive transformants and mitigating chimerism has indeed been previously employed in grapevine [43–45]. In our study, we demonstrated that the DsRed2 fluorescent probe also allows a non-destructive selection process in transformed grapevine cells and then reduces the risk to obtain chimeric plantlets (Figs. 2 and 3). The reporter gene *DsRed2* has proven to be useful in tobacco transgenic plantlets [31]. Regarding gene editing efficiency, we focused on other parameters at molecular scale. Indeed, many studies have shown that gene editing efficiency can be affected by sgRNA design, promoter choice for *Cas9* expression, and the nucleotide sequence of the *Cas9* gene, including the number of NLS domains, the presence of intronic sequence, and the nature of codons [22, 23, 28, 46].

In grapevine, a recent study compared sgRNA with different GC content and found that those with more than 50% GC had higher target gene efficiency than those with less than 50% [22]. Therefore, in our study we selected three sgRNAs with a GC content between 55% and 60%. Despite a similar GC content among the three sgRNAs targeting the same gene, we observed a significant difference in mutation efficiency among them (Fig. 4b). SgRNA3 seemed to greatly increase target gene efficiency compared to sgRNA1 and 2, for all three constructs (Fig. 4b). The analysis of the 3D structure using the RNAfold software for each sgRNA revealed that sgRNA3 exhibits the most stable conformation [42, 47]. Consequently, the analysis of 3D structure for sgRNA design appears to be an important step. Focusing on the most efficient construct (construct 2), sgRNA1 also seemed more efficient than sgRNA2. These results prove that GC content is not the only factor that influences sgRNA efficiency. As other parameter, Doench and his collaborators [48] proposed a sgRNA model sequence that highlighted two nucleotides (‘A’ and ‘G’) located before the PAM site as favourable for target gene efficiency. Interestingly, in our study sgRNA1 and sgRNA3 possess the nucleotide ‘A’ before the PAM site, while sgRNA2 had a ’C’, which was unfavourable. The sgRNA efficiency results seemed to agree with the sgRNA model sequence proposed by Doench *et al* [48]. and could be another criterion to design and select sgRNA with an ‘A’ or a ‘G’ before the PAM site to improve target gene efficiency. On the other hand, the first nucleotide of the PAM site could also affect target gene efficiency, but no correlation could be established in our study [48].

Several studies on different plant species have shown that the promoter used for *Cas9* expression is another important parameter to enhance target gene efficiency. In *A. thaliana*, a comparison of different promoters, including *35S*, *RPS5a*, *YAO*, *UBI10*, *ICU2* and *MGE1* promoters, revealed a high variability of mutation rates, with the highest efficiency achieved with *RPS5a* promoter [28]. In our study, the *zCas9i* expression was driven using the *35S* or *RPS5a* promoter from cauliflower mosaic virus (CaMV) and *A. thaliana*, respectively. Interestingly, in grapevine, the comparison of results obtained with construct 2 and 3 showed that the target gene efficiency was significantly higher using the *35S* promoter (Fig. 4). These results were directly correlated with *Cas9* expression and the amount of protein available in the cells (Fig. 5), regardless of the targeted gene chosen (Fig. S11). Surprisingly, construct 3, which consisted of expressing *zCas9i* under the control of the *RPS5a* promoter, had a very low expression level with no detected protein at the plantlet stage (Fig. 5b). Nevertheless, we observed a mutation rate of 69% and obtained biallelic mutants (Fig. 4a). One hypothesis is that *RPS5a*– mediated *Cas9* expression could occur early in the selection and somatic embryogenesis process to induce target gene mutations and disappear when plantlets regenerate. Indeed, in *A. thaliana*, *RPS5a* promoter is mainly expressed during embryonic development [49, 50]. In future work, it would be interesting to control *zCas9i* expression with endogenous grapevine promoter such as *RPS5a* orthologs or *VvUBQ2* promoters. Indeed, only one previous study in grapevine showed that *Cas9* transcripts were 8 times more expressed under the control of *UBQ2* promoter than under *35S* promoter [27]. The promoter used for sgRNA expression could also be investigated. Ren et al [27] showed a higher efficiency by using *AtU6-26* orthologous promoters from grapevine.

The last improvement to consider is the nucleotide sequence of *Cas9* gene. Firstly, the number of NLS domains plays an important role in the Cas9 targeting to the nucleus. A study in *A. thaliana* and *Nicotiana benthamiana* clearly demonstrated that targeting of Cas9 to the nucleus was significantly better with two NLS domains [23]. Interestingly, these authors also showed that Cas9 with two NLS domains but without intronic sequence in its gene was not expressed. That is why we chose to compare the human codon-optimized Cas9 without intron and only one NLS domain with the maize codon-optimized Cas9 including 13 intronic sequences and 2 NLS domains. The second crucial step to optimize target gene efficiency in grapevine is the introduction of intronic sequence in the *Cas9* gene sequence, as described by Grützner et al. [23]. The comparison of the gene editing efficiency between two constructs under the same *35S* promoter using two distinct codon-optimized *hCas9* and *zCas9i*, without and with intron, respectively, showed a significant difference in the mutation rate. The *zCas9i* possessing introns lead to a high frequency of biallelic mutations on target gene without increasing the mutation rate in off-target sites (Figs. 4, 5 and 6). Correlated with these results, we highlighted an increased expression of z*Cas9i* possessing intronic sequences (Fig. 5). According to the results obtained in *A. thaliana* by Grützner et al. [23], the addition of intronic sequences from the *A. thaliana* genome to the coding sequence of *Cas9* gene seems to be the most important parameter to improve gene editing efficiency compared to codon optimization, considered for a long time as the most important parameter in plant genome editing [23, 46, 51, 52]. By comparison with the only study showing a biallelic mutation rate in a significant number of grapevine plantlets, the percentage of biallelic mutations in the first generation was around 20% with a plant codon-optimized *Cas9* gene without intronic sequences, compared to 100% in our study with a *Cas9* containing intronic sequences [19]. This comparison supports the importance of introducing introns in *Cas9* gene sequence. The addition of 13 intronic sequences and the increase of *Cas9* transcripts could be due to an intron-mediated transcriptional enhancement and/or a better mRNA stability [53, 54]. Using intronic sequences from grapevine genome could be a new way to further improve genome editing in grapevine. Indeed, despite a high efficiency of gene editing leading to nucleotide addition or deletion, we did not observe long deletion corresponding to the absence of complete DNA sequence between 2 sgRNA as already observed in Arabidopsis or rice [23, 24]. The absence of long deletion could be explained by the fragment size between the two most effective sgRNA1 and sgRNA3 (757 bp). Indeed, Wang et al. [19] obtained some transgenic lines with long deletion up to 152 bp which is shorter than our sequence between sgRNA1 and sgRNA3.

To summarize, by an original comparison of three independent constructs, we demonstrated that we are able to obtain 100% biallelic mutants leading to knockout plantlets without increasing off-target mutations in *V. vinifera* cv. ‘Chardonnay’. Our work greatly improves the target gene efficiency to induce mutations in grapevine leading to non-functional proteins. Our innovative technology could be useful in the identification of new *S* genes to obtain new grapevine cv. highly resistant against pests or diseases. For example, the LysM receptor-like kinases family described in *A. thaliana* showed that one member named AtLYK3 is involved in inhibition of plant defence [55]. Thus, KO mutation in this gene should lead to plantlets with increased immune responses. In wheat, mutation in the *enhanced disease resistance 1* gene, leading to non-functional protein EDR1, enhanced the resistance against powdery mildew [56]. Last, a non-functional sugar transporter AtSWEET4 in *A. thaliana* increased its resistance against *Botrytis cinerea* [57]. Interestingly, its orthologous in grapevine (VvSWEET4) seems to be also involved in the interaction with *Botrytis cinerea* [57]. Future studies targeting these putative *S* genes in grapevine could be investigated using our highly efficient CRISPR/Cas9 system to quickly generate homozygous mutants in different *V. vinifera* cultivars. Combination of multiple non-functional *S* genes seems a durable strategy to obtain resistant grapevine plants against different diseases.

For future prospects, CRISPR/Cas9 technology can be adapted for other purposes such as to up- or down-regulate specific genes involved in known functions or to precisely modify one or several nucleotides with the base- or prime-editing technology. Modification of expression using a deactivated Cas9 (dCas9) coupled with a transcription activator domain to enhance gene expression could also be an alternative solution to improve resistance to biotic stress [58]. Only one group has succeeded in modifying the expression of a target gene in grapevine with this approach [59]. In contrast, no study demonstrating base- or prime-editing has been reported in grapevine. This approach, mostly developed in rice, could be a real agronomic lever for grapevine selection and improvement process [60]. A recent study retraced evolution and domestication events in grapevine by sequencing 3525 accessions from all worldwide regions [61]. These genetic resources could enable the identification of new alleles correlated with resistance and susceptibility of grapevine accessions to different stresses. Base-editing could be used to modify specific amino acids known to improve plant resistance. This technology aims to mimic existing genetic variability to obtain cultivars with great agronomical interest and mutated traits that enhance resistance to environmental biotic and abiotic stresses, as well as improved yield or quality.

## Conclusion

In conclusion, a decade of research on CRISPR/Cas9 technologies has yielded numerous advancements in plant genome editing, spanning various molecular parameters and methodologies. This period serves as a crucial foundation for future endeavors and breeding initiatives [62]. Our optimization of the selection process, using DsRed2 and zCas9i for target gene editing via T-DNA, holds promise for genomic functional studies and fundamental research. However, the integration of our method into traditional breeding practices remains complex, time-consuming and poorly accepted by public opinion. Conversely, the use of T-DNA-free genome editing presents a promising challenge in grapevine research. Notably, some studies have successfully generated T-DNA-free edited plants through protoplast regeneration establishing this approach as the most suitable strategy, particularly in light of new regulations proposed by the European committee [13, 42, 45, 63]. This innovative technique is poised to facilitate accelerated breeding programs in grapevine, essential for addressing the future challenges associated with climate change.

## Materials and methods

### Plant material and growth

Stabilized embryogenic cells from anther of ‘Chardonnay’ variety (*Vitis vinifera* L.) were cultivated in 25 mL liquid GM medium as described in Ren et al. [22]. under stirring at 25 °C in the dark. Half of cells were pricked out in a new medium every 7 days. To initiate somatic embryogenesis, embryogenic cells were pricked out in GM medium without NOA (2-Naphthoxyacetic acid). Newly formed embryos are then transferred in McCown (M0220) Media (pH 5,8) supplemented with 30 g/L saccharose, 0.2 mg/L Indole 3 Butyric acid and 7 g/L Agar for plantlets regeneration under light (50 µmoL m^− 2^ s^− 1^).

### Cloning of target gene and design of sgRNA

First-strand cDNAs were generated from total RNA extracted from leaves of grapevine cv. “Chardonnay” using transcriptase inverse SuperScript IV (Invitrogen 18,090,010). Full length of 1869 bp target gene (Vitvi05g00623) was then amplified using specific primers (Table S1) prior to cloning into PCR8 entry vector with pCR8/GW/TOPO TA Cloning kit (K250020). Verification of target gene sequence for sgRNA design was subcontracted by Sanger sequencing (Eurofins genomics).

SgRNAs for CRISPR/Cas9 were designed from the online CRISPOR database (http://crispor.tefor.net/). Three sgRNAs (Fig. 1a) were selected according to Doench ’16 score for predicted efficiency [64], Out-of Frame score, GC content, and potential off-targets sites.

### Assembly of CRISPR/cas9 plasmid construction

All plasmid constructions have been generated with GoldenBraid system [65]. Human codon optimized Cas9 construction (GB0575) was obtained from Addgene company. *Zea mays* codon optimized Cas9 (zCas9i) sequence and *RPS5a* (At3g11940) promoter were isolated from pAGM65879 available on Addgene website (https://www.addgene.org/). zCas9i was then domesticated in the GoldenBraid system to generate construction presented in Fig. 1b.

### Embryogenic cells transformation and antibiotic selection

Final plasmids for each construction were introduced in *A. tumefaciens* EHA105 strain by electroporation method with Gene Pulser Xcell Microbial System (1,652,662, Biorad). Selected and verified *A. tumefaciens* cells were cultivated up to saturation in YEB media at pH7.2 supplemented with 5mM MgSO_4_ and corresponding antibiotics. Grapevine embryogenic cells were transformed by co-cultivation with *A. tumefaciens* culture with an absorbance of 0.3 during 30 min. Then embryogenic cells were transferred in GM medium with agar (0.7%) and incubated during 72 h at 26 °C. Embryogenic cells were then re-suspended in liquid GM medium and washed three times. After that, grapevine embryogenic cells were cultivated in GM supplemented with 200 µg/ml cefotaxime and 2 µg/ml paromomycin for selection. Liquid GM medium and antibiotics were changed every 7 days.

### Fluorescent selection of embryogenic cells, embryos and plantlets

For fluorescent selection with DsRed2 (FPbase ID: TXLFX), plant material was disposed on sterile agar plate with suitable medium. Observations were carried out in the ImaFlow core facility and conducted on an Axio Zoom.V16 (Zeiss) equipped with an AxioCam HRm camera (Zeiss) at the X10 objective magnifications and filter 63 HE mRFP (excitation wavelength at 561 nm and emission wavelength at 587 nm). Pictures were then treated with ZEN software.

### PCR amplification of exogenous T-DNA insertion, targeted gene and off-targets

Specific primers for hCas9, zCas9i and targeted gene (Table S1) were used for PCR amplification with the Phire Hot Start II DNA Polymerase from Phire Plant Direct PCR kit (F-130WH) for embryogenic cells and plantlets material. For amplification, thermocycler program started at 98 °C during 5 min. Then 35 cycles composed of denaturation step at 98 °C during 10 s, hybridization step at corresponding temperature according primers used (Table S1) and elongation step of 20 s at 72 °C. Cycles were followed by a final elongation step at 72 °C during 5 min. Amplicons were separated on 1% agarose gel with 1 kb plus marker (ThermoFisher scientific-10787018). Amplicons corresponding to targeted gene were directly purified and sequenced by Sanger method (Eurofins genomics).

### Total RNA extraction and real-time quantitative PCR (RT-qPCR) analysis

Total RNA was extracted from leaves of one month-aged plantlets using Spectrum plant total RNA kit (Sigma Aldrich) including DNAse I treatment and quantified by NanoDrop Spectrophotometer 2000 (Thermofisher) with three technical replicates. First-strand cDNAs were synthesized with transcriptase inverse SuperScript IV and used as a template for the RT-qPCR experiments. RT-qPCR analyses were performed with ABsolute QPCR Mix SYBR Green low ROX (Thermofisher) using 10ng of total RNA in 5 µl. Reactions were performed in duplicate using between six and ten independent biological samples. Amplification of cDNA was carried out with a ViiA7 (Applied Biosystem) thermocycler applying following program: 2 min of denaturation and activation of DNA polymerase at 95 °C followed by 40 cycles composed of 3 steps (Denaturation at 95 °C during 15 s, primer hybridization at 61 °C during 30 s and PCR extension at 72 °C during 30 s). A final step consisting of 15 s at 95 °C, 1 min at primer hybridization temperature (61 °C) then increasing by incremented temperature of 0.05 °C/sec until 95 °C during allowing to obtain the melting curves to verify primer specificity. All amplifications were analysed with QuantStudio Real Time PCR software v1.2 using threshold of 0.2 to obtain Cycle Threshold (C_T_) values. Expression level of both *Cas9* genes including *hCAS9* and *zCAS9i* were normalized with the corresponding C_T_ values of two control housekeeping genes (*VvEF1-alpha* and *VvVATP16*) [66, 67]. Standard curves were obtained for housekeeping and *Cas9* genes by successive dilution with known quantities of cDNA amplicons for each gene with specific primers provided in supplementary table S1.

### Immunodetection of Cas9 in grapevine plantlets

Leaves from plantlets were firstly placed in liquid nitrogen and grinded with the TissueLyserII (Qiagen). Total proteins were extracted using Laemmli buffer (0.125 M Tris-HCl pH 6.8, 2%SDS, 100µM Dithiothreitol) quantified with RCDC protein assay Kit I (Biorad). Total extracts were then dropped in 10% SDS-PAGE gels. After migration, proteins were transferred either in Coomassie blue for quantification staining or in nitrocellulose membranes (0.2 μm). After saturation, a rabbit polyclonal anti-Cas9 antibody (Agrisera AS163690) derived from *Streptococcus thermophilus* Cas9/Csn1 protein sequence, was used for immunodetection. Revealing was carried out with an Amersham^™^ ImageQuant^™^ 800 (Cytiva) using ECL^™^ prime as immunodetection reagent. Quantification of Cas9 intensity was normalized by total proteins highlights by Coomassie blue staining with the ImageQuant software.

### Electronic supplementary material

Below is the link to the electronic supplementary material.

Supplementary Material 1

## Data Availability

All data generated or analysed during this study are included in this published article and its supplementary information files.
