# Publisher Correction: New improvements in grapevine genome editing: high efficiency biallelic homozygous knock-out from regenerated plantlets by using an optimized zCas9i

**DOI:** 10.1186/s13007-024-01204-4

**Published:** 2024-06-14

**Authors:** Jérémy Villette, Fatma Lecourieux, Eliot Bastiancig, Marie-Claire Héloir, Benoit Poinssot

**Affiliations:** 1 Agroécologie, INRAE, Institut Agro, Université de Bourgogne, Dijon, France; 2 UMR1287 EGFV, CNRS, Université de Bordeaux, INRAE, Bordeaux Sciences Agro, ISVV, Villenave d’Ornon, Dijon, France

**Correction: Plant Methods (2024) 20:45** 10.1186/s13007-024-01173-8

In this article [1] the wrong figure appeared as Fig. 5. In Fig. 5a and 5b, between the x-axis and the axis legends, there is a line that was not initially present in figure.

Uncorrected figure:
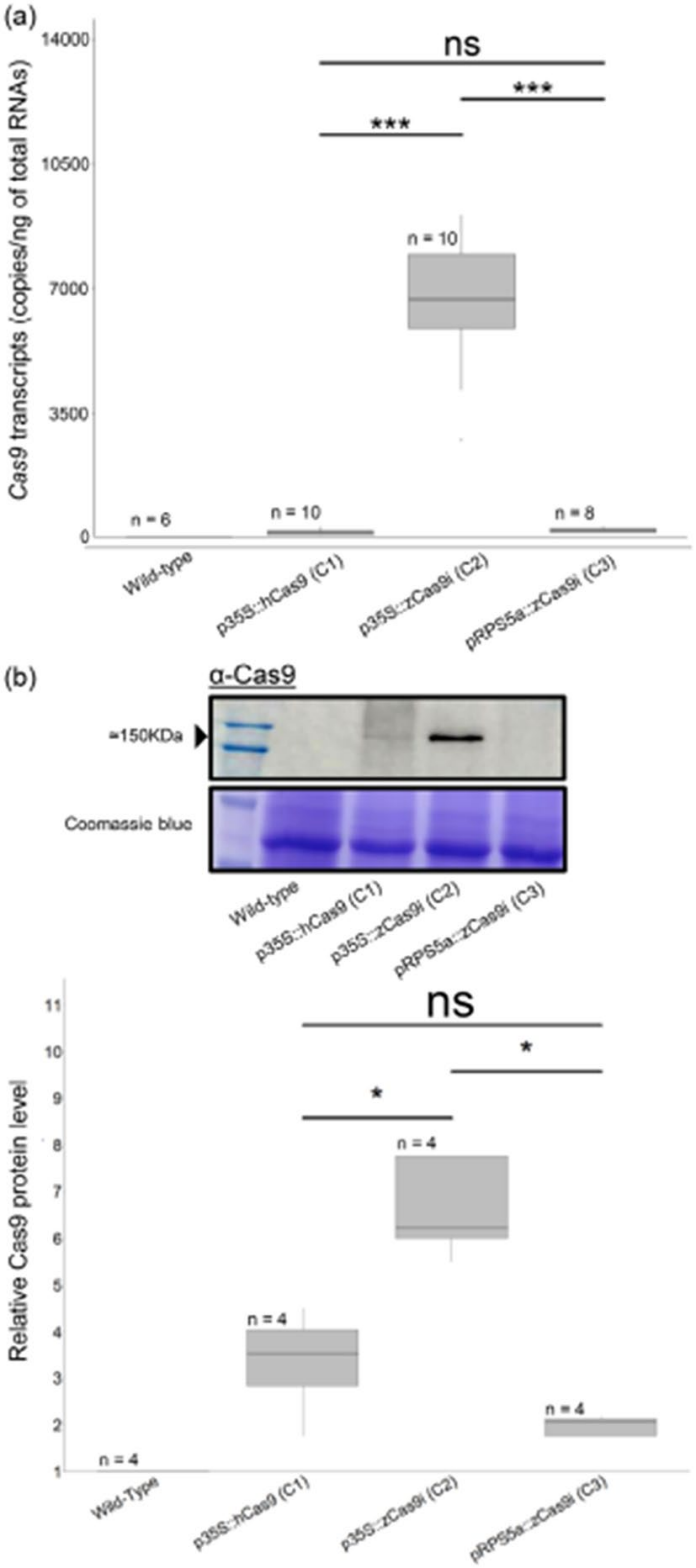


The figure should have appeared as shown below.

Corrected figure
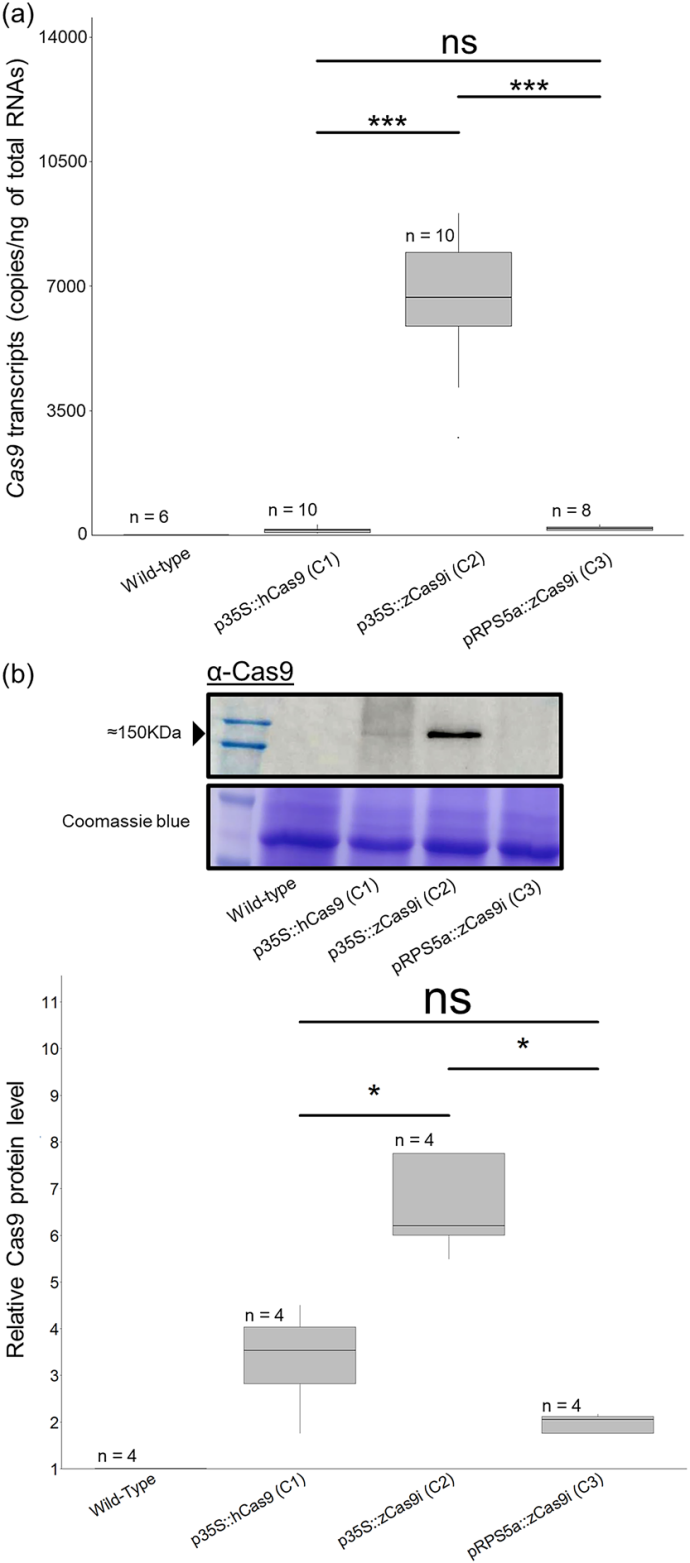


## References

[1] Villette J, Lecourieux F, Bastiancig E, Héloir M-C, Poinssot B (2024). New improvements in grapevine genome editing: high efficiency biallelic homozygous knock-out from regenerated plantlets by using an optimized zCas9i. Plant Methods.

