# Supplementary material for: New improvements in grapevine genome editing: high efficiency biallelic homozygous knock-out from regenerated plantlets by using an optimized zCas9i
Source: Plant Methods. 2024 Mar 18;20:45. doi: 10.1186/s13007-024-01173-8 (PMC10949784; doi:10.1186/s13007-024-01173-8)
Supplement: Supplementary file 1 — Supplementary Material 1 [file 13007_2024_1173_MOESM1_ESM.pdf]

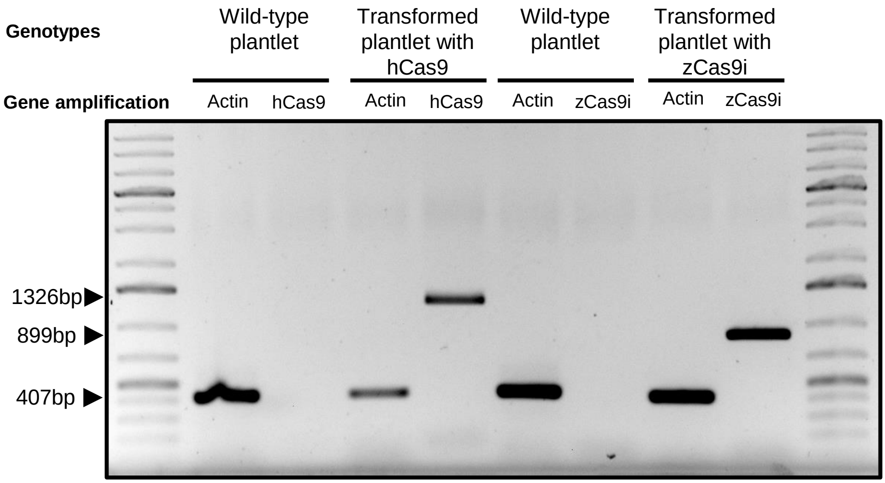

Figure S1. Specificity of Cas9 gene amplification on genomic DNA. PCR amplification of actin for wild-type and selected plantlets as positive control with a PCR product size of 407bp. PCR amplification of two different Cas9 gene on wild-type and corresponding transformed plantlets. PCR product size are 1326bp and 899bp for hCas9 and zCas9i gene respectively.

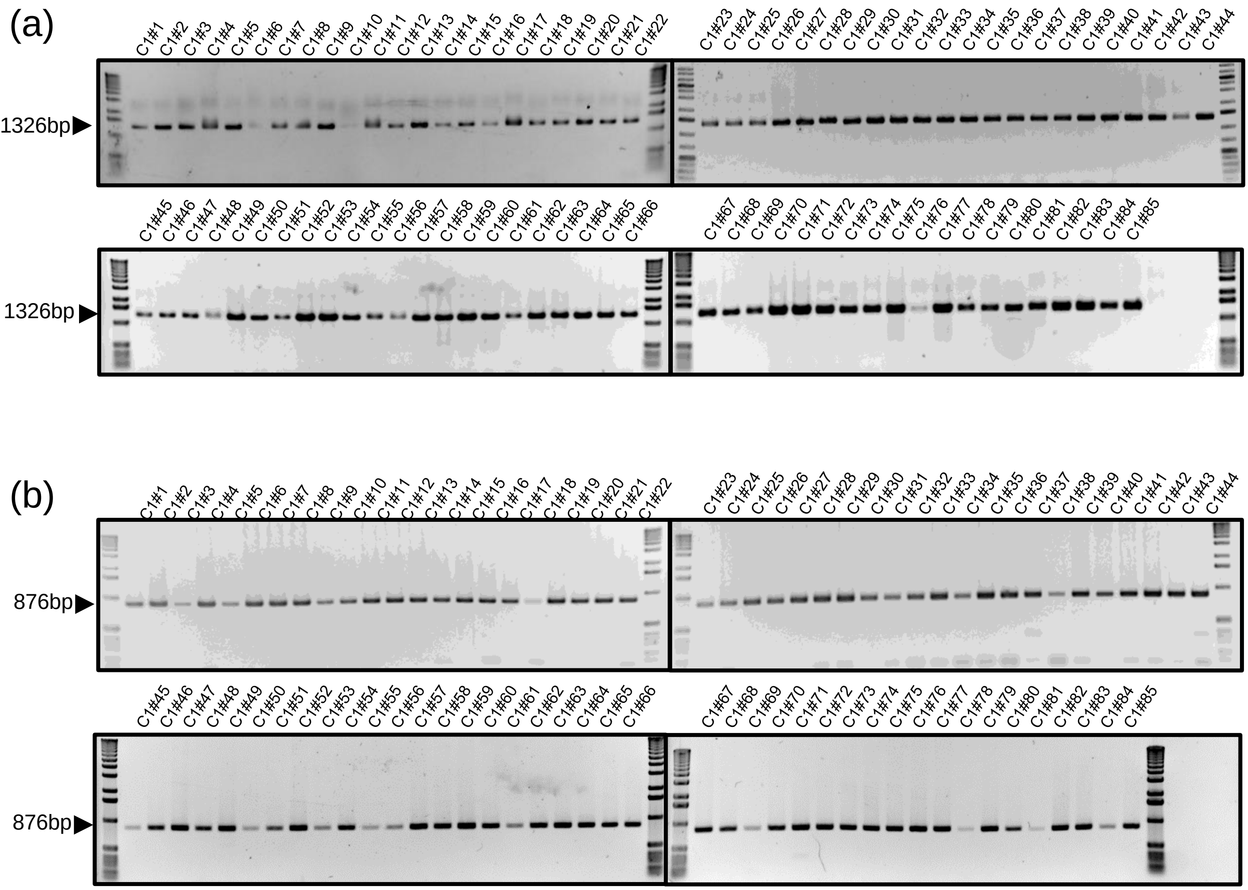

Figure S2. Genotyping of 85 selected plantlets transformed with the construct 1 (*p35S::hCas9*) for mutation rate analysis. (a) PCR amplification of a part of the *Cas9* gene (1326bp) in 85 independent plantlets showing the presence of T-DNA insertion. (b) PCR amplification of the target gene (876bp) on the same 85 plantlets for sequencing analysis and study of target gene editing efficiency.

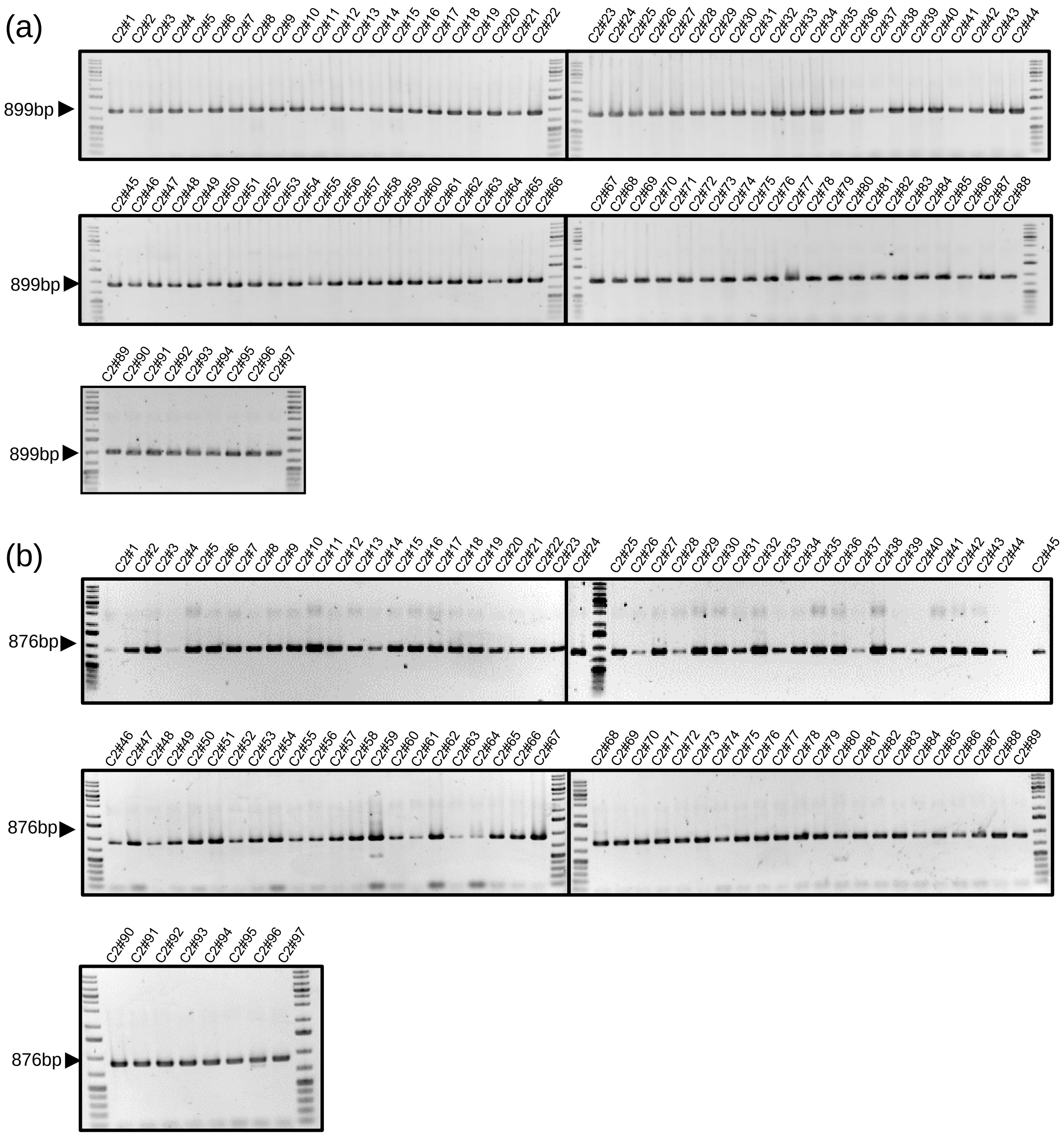

Figure S3. Genotyping of 97 selected plantlets transformed with the construct 2 (*p35S::zCas9i*) for mutation rate analysis. (a) PCR amplification of a part of the *Cas9* gene (899bp) in 97 independent plantlets showing the presence of T-DNA insertion. (b) PCR amplification of the target gene (876bp) of the same 97 plantlets for sequencing analysis and study of target gene editing efficiency.

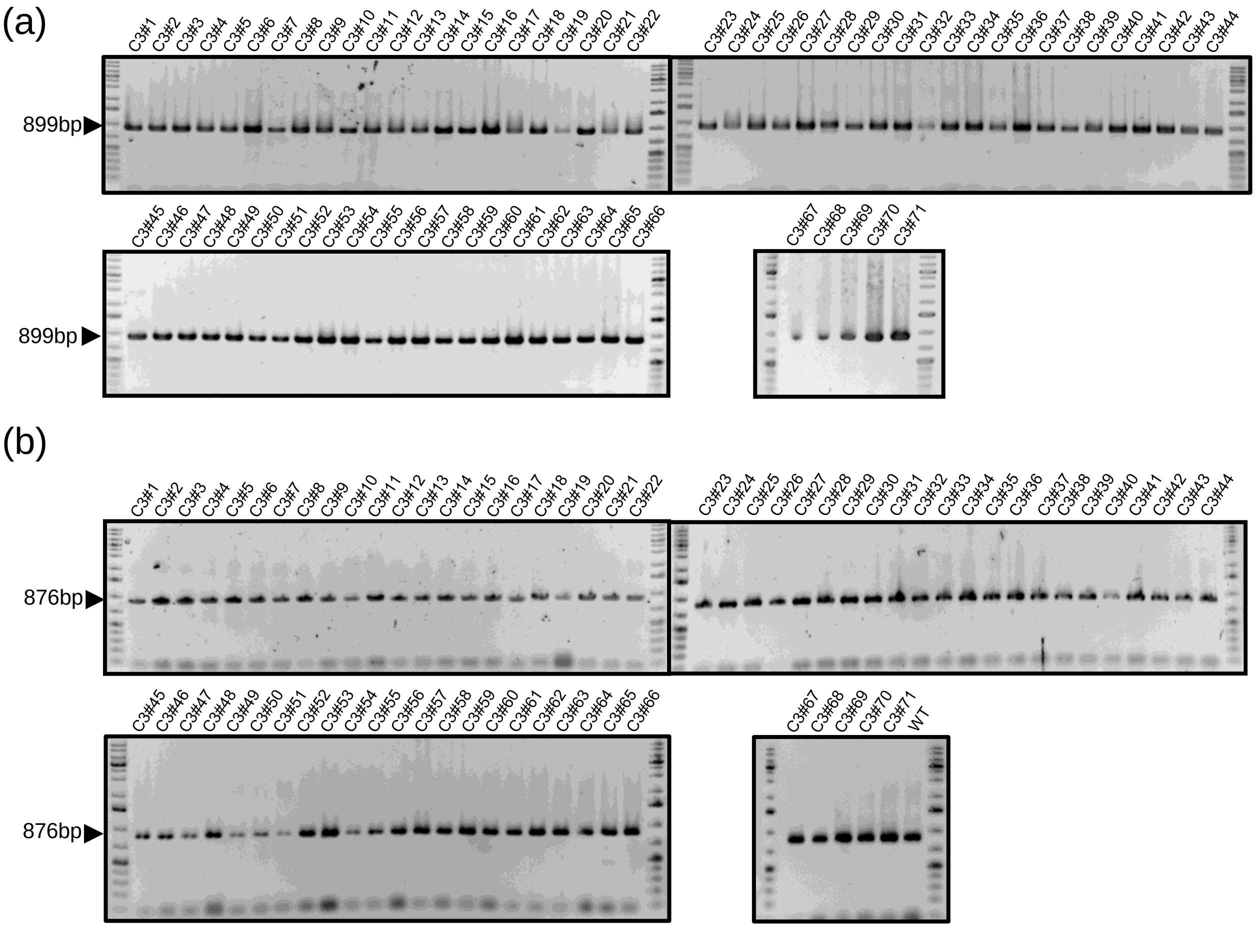

Figure S4. Genotyping of 71 selected plantlets transformed with the construct 3 (*pRPS5a::zCas9i*) for mutation rate analysis. (a) PCR amplification of a part of the *Cas9* gene (899bp) in 71 independent plantlets showing the presence of T-DNA insertion. (b) PCR amplification of the target gene (876bp) of the same 71 plantlets for sequencing analysis and study of target gene editing efficiency.

		sgRNA 1	sgRNA 2	sgRNA 3
WT	Allele 1	268 AACCCCCTTGTCGCCGGTCAAAGCCTCTTCG 298	…707 CACAGCCTAATGTGACCGCTTCGCCGGCGAC 737	…1014 GTTTAGTGAGCGTTCCCTG-AT-CCAAGGGTCT 1044
	Allele 2	268 AACCCCCTTGTCGCCGGTCAAAGCCTCTTCG 298	…707 CACAGCCTAATGTGACCGCTTCGCCGGCGAC 737	…1014 GTTTAGTGAGCGTTCCCTG-AT-CCAAGGGTCT 1044
C1_1	Allele 1	268 AACCCCCTTGTCGCCGGTCAAAGCCTCTTCG 298	…707 CACAGCCTAATGTGACCGCTTCGCCGGCGAC 737	…1014 GTTTAGTGAGCGTTCCCTG-AT-CCAAGGGTCT 1044
	Allele 2	268 AACCCCCTTGTCGCCGGTCAAAGCCTCTTCG 298	…707 CACAGCCTAATGTGACCGCTTCGCCGGCGAC 737	…1014 GTTTAGTGAGCGTTCCCTG-AT-CCAAGGGTCT 1044
C1_2	Allele 1	268 AACCCCCTTGTCGCCGGTCAAAGCCTCTTCG 298	…707 CACAGCCTAATGTGACCGCTTCGCCGGCGAC 737	…1014 GTTTAGTGAGCGTTCCCTG-AT-CCAAGGGTCT 1044
	Allele 2	268 AACCCCCTTGTCGCCGGTCAAAGCCTCTTCG 298	…707 CACAGCCTAATGTGACCGCTTCGCCGGCGAC 737	…1014 GTTTAGTGAGCGTTCCCTG-AT-CCAAGGGTCT 1044
C1_3	Allele 1	268 AACCCCCTTGTCGCCGGTCAAAGCCTCTTCG 298	…707 CACAGCCTAATGTGACCGCTTCGCCGGCGAC 737	…1014 GTTTAGTGAGCGTTCCCTG-AT-CCAAGGGTCT 1044
	Allele 2	268 AACCCCCTTGTCGCCGGTCAAAGCCTCTTCG 298	…707 CACAGCCTAATGTGACCGCTTCGCCGGCGAC 737	…1014 GTTTAGTGAGCGTTCCCTG-AT-CCAAGGGTCT 1044
C1_4	Allele 1	268 AACCCCCTTGTCGCCGGTCAAAGCCTCTTCG 298	…707 CACAGCCTAATGTGACCGCTTCGCCGGCGAC 737	…1014 GTTTAGTGAGCGTTCCCTG-AT-CCAAGGGTCT 1044
	Allele 2	268 AACCCCCTTGTCGCCGGTCAAAGCCTCTTCG 298	…707 CACAGCCTAATGTGACCGCTTCGCCGGCGAC 737	…1014 GTTTAGTGAGCGTTCCCTG-AT-CCAAGGGTCT 1044
C1_5	Allele 1	268 AACCCCCTTGTCGCCGGTCAAAGCCTCTTCG 298	…707 CACAGCCTAATGTGACCGCTTCGCCGGCGAC 737	…1014 GTTTAGTGAGCGTTCCCTG-AT-CCAAGGGTCT 1044
	Allele 2	268 AACCCCCTTGTCGCCGGTCAAAGCCTCTTCG 298	…707 CACAGCCTAATGTGACCGCTTCGCCGGCGAC 737	…1014 GTTTAGTGAGCGTTCCCTG-AT-CCAAGGGTCT 1044
C1_6	Allele 1	268 AACCCCCTTGTCGCCGGTCAAAGCCTCTTCG 298	…707 CACAGCCTAATGTGACCGCTTCGCCGGCGAC 737	…1014 GTTTAGTGAGCGTTCCCTG-AT-CCAAGGGTCT 1044
	Allele 2	268 AACCCCCTTGTCGCCGGTCAAAGCCTCTTCG 298	…707 CACAGCCTAATGTGACCGCTTCGCCGGCGAC 737	…1014 GTTTAGTGAGCGTTCCCTG-AT-CCAAGGGTCT 1044
C1_7	Allele 1	268 AACCCCCTTGTCGCCGGTCAAAGCCTCTTCG 298	…707 CACAGCCTAATGTGACCGCTTCGCCGGCGAC 737	…1014 GTTTAGTGAGCGTTCCCTG-AT-CCAAGGGTCT 1044
	Allele 2	268 AACCCCCTTGTCGCCGGTCAAAGCCTCTTCG 298	…707 CACAGCCTAATGTGACCGCTTCGCCGGCGAC 737	…1014 GTTTAGTGAGCGTTCCCTG-AT-CCAAGGGTCT 1044
C1_8	Allele 1	268 AACCCCCTTGTCGCCGGTCAAAGCCTCTTCG 298	…707 CACAGCCTAATGTGACCGCTTCGCCGGCGAC 737	…1014 GTTTAGTGAGCGTTCCCTG-AT-CCAAGGGTCT 1044
	Allele 2	268 AACCCCCTTGTCGCCGGTCAAAGCCTCTTCG 298	…707 CACAGCCTAATGTGACCGCTTCGCCGGCGAC 737	…1014 GTTTAGTGAGCGTTCCCTG-AT-CCAAGGGTCT 1044
C1_9	Allele 1	268 AACCCCCTTGTCGCCGGTCAAAGCCTCTTCG 298	…707 CACAGCCTAATGTGACCGCTTCGCCGGCGAC 737	…1014 GTTTAGTGAGCGTTCCCTG-AT-CCAAGGGTCT 1044
	Allele 2	268 AACCCCCTTGTCGCCGGTCAAAGCCTCTTCG 298	…707 CACAGCCTAATGTGACCGCTTCGCCGGCGAC 737	…1014 GTTTAGTGAGCGTTCCCTGAAT-CCAAGGGTCT 1045
C1_10	Allele 1	268 AACCCCCTTGTCGCCGGTCAAAGCCTCTTCG 298	…707 CACAGCCTAATGTGACCGCTTCGCCGGCGAC 737	…1014 GTTTAGTGAGCGTTCCCTG-AT-CCAAGGGTCT 1044
	Allele 2	268 AACCCCCTTGTCGCCGGTCAAAGCCTCTTCG 298	…707 CACAGCCTAATGTGACCGCTTCGCCGGCGAC 737	…1014 GTTTAGTGAGCGTTCCCTG-AT-CCAAGGGTCT 1044
C1_11	Allele 1	268 AACCCCCTTGTCGCCGGTCAAAGCCTCTTCG 298	…707 CACAGCCTAATGTGACCGCTTCGCCGGCGAC 737	…1014 GTTTAGTGAGCGTTCCCTG-AT-CCAAGGGTCT 1044
	Allele 2	268 AACCCCCTTGTCGCCGGTCAAAGCCTCTTCG 298	…707 CACAGCCTAATGTGACCGCTTCGCCGGCGAC 737	…1014 GTTTAGTGAGCGTTCCCTG-AT-CCAAGGGTCT 1044
C1_12	Allele 1	268 AACCCCCTTGTCGCCGGTCAAAGCCTCTTCG 298	…707 CACAGCCTAATGTGACCGCTTCGCCGGCGAC 737	…1014 GTTTAGTGAGCGTTCCCTG-AT-CCAAGGGTCT 1044
	Allele 2	268 AACCCCCTTGTCGCCGGTCAAAGCCTCTTCG 298	…707 CACAGCCTAATGTGACCGCTTCGCCGGCGAC 737	…1014 GTTTAGTGAGCGTTCCCTG-AT-CCAAGGGTCT 1044
C1_13	Allele 1	268 AACCCCCTTGTCGCCGGTCAAAGCCTCTTCG 298	…707 CACAGCCTAATGTGACCGCTTCGCCGGCGAC 737	…1014 GTTTAGTGAGCGTTCCCTG-AT-CCAAGGGTCT 1044
	Allele 2	268 AACCCCCTTGTCGCCGGTCAAAGCCTCTTCG 298	…707 CACAGCCTAATGTGACCGCTTCGCCGGCGAC 737	…1014 GTTTAGTGAGCGTTCCCTG-AT-CCAAGGGTCT 1044
C1_14	Allele 1	268 AACCCCCTTGTCGCCGGTCAAAGCCTCTTCG 298	…707 CACAGCCTAATGTGACCGCTTCGCCGGCGAC 737	…1014 GTTTAGTGAGCGTTCCCTG-AT-CCAAGGGTCT 1044
	Allele 2	268 AACCCCCTTGTCGCCGGTCAAAGCCTCTTCG 298	…707 CACAGCCTAATGTGACCGCTTCGCCGGCGAC 737	…1014 GTTTAGTGAGCGTTCCCTG-AT-CCAAGGGTCT 1044
C1_15	Allele 1	268 AACCCCCTTGTCGCCGGTCAAAGCCTCTTCG 298	…707 CACAGCCTAATGTGACCGCTTCGCCGGCGAC 737	…1014 GTTTAGTGAGCGTTCCCTG-AT-CCAAGGGTCT 1044
	Allele 2	268 AACCCCCTTGTCGCCGGTCAAAGCCTCTTCG 298	…707 CACAGCCTAATGTGACCGCTTCGCCGGCGAC 737	…1014 GTTTAGTGAGCGTTCCCTG-AT-CCAAGGGTCT 1044
C1_16	Allele 1	268 AACCCCCTTGTCGCCGGTCAAAGCCTCTTCG 298	…707 CACAGCCTAATGTGACCGCTTCGCCGGCGAC 737	…1014 GTTTAGTGAGCGTTCCCTG-AT-CCAAGGGTCT 1044
	Allele 2	268 AACCCCCTTGTCGCCGGTCAAAGCCTCTTCG 298	…707 CACAGCCTAATGTGACCGCTTCGCCGGCGAC 737	…1014 GTTTAGTGAGCGTTCCCTG-AT-CCAAGGGTCT 1044
C1_17	Allele 1	268 AACCCCCTTGTCGCCGGTCAAAGCCTCTTCG 298	…707 CACAGCCTAATGTGACCGCTTCGCCGGCGAC 737	…1014 GTTTAGTGAGCGTTCCCTG-AT-CCAAGGGTCT 1044
	Allele 2	268 AACCCCCTTGTCGCCGGTCAAAGCCTCTTCG 298	…707 CACAGCCTAATGTGACCGCTTCGCCGGCGAC 737	…1014 GTTTAGTGAGCGTTCCCTG-AT-CCAAGGGTCT 1044
C1_18	Allele 1	268 AACCCCCTTGTCGCCGGTCAAAGCCTCTTCG 298	…707 CACAGCCTAATGTGACCGCTTCGCCGGCGAC 737	…1014 GTTTAGTGAGCGTTCCCTG-AT-CCAAGGGTCT 1044
	Allele 2	268 AACCCCCTTGTCGCCGGTCAAAGCCTCTTCG 298	…707 CACAGCCTAATGTGACCGCTTCGCCGGCGAC 737	…1014 GTTTAGTGAGCGTTCCCTG-AT-CCAAGGGTCT 1044
C1_19	Allele 1	268 AACCCCCTTGTCGCCGGTCAAAGCCTCTTCG 298	…707 CACAGCCTAATGTGACCGCTTCGCCGGCGAC 737	…1014 GTTTAGTGAGCGTTCCCTG-AT-CCAAGGGTCT 1044
	Allele 2	268 AACCCCCTTGTCGCCGGTCAAAGCCTCTTCG 298	…707 CACAGCCTAATGTGACCGCTTCGCCGGCGAC 737	…1014 GTTTAGTGAGCGTTCCCTG-AT-CCAAGGGTCT 1044
C1_20	Allele 1	268 AACCCCCTTGTCGCCGGTCAAAGCCTCTTCG 298	…707 CACAGCCTAATGTGACCGCTTCGCCGGCGAC 737	…1014 GTTTAGTGAGCGTTCCCTG-AT-CCAAGGGTCT 1044
	Allele 2	268 AACCCCCTTGTCGCCGGTCAAAGCCTCTTCG 298	…707 CACAGCCTAATGTGACCGCTTCGCCGGCGAC 737	…1014 GTTTAGTGAGCGTTCCCTG-AT-CCAAGGGTCT 1044
C1_21	Allele 1	268 AACCCCCTTGTCGCCGGTCAAAGCCTCTTCG 298	…707 CACAGCCTAATGTGACCGCTTCGCCGGCGAC 737	…1014 GTTTAGTGAGCGTTCCCTG-AT-CCAAGGGTCT 1044
	Allele 2	268 AACCCCCTTGTCGCCGGTCAAAGCCTCTTCG 298	…707 CACAGCCTAATGTGACCGCTTCGCCGGCGAC 737	…1014 GTTTAGTGAGCGTTCCCTG-AT-CCAAGGGTCT 1044
C1_22	Allele 1	268 AACCCCCTTGTCGCCGGTCAAAGCCTCTTCG 298	…707 CACAGCCTAATGTGACCGCTTCGCCGGCGAC 737	…1014 GTTTAGTGAGCGTTCCCTG-AT-CCAAGGGTCT 1044
	Allele 2	268 AACCCCCTTGTCGCCGGTCAAAGCCTCTTCG 298	…707 CACAGCCTAATGTGACCGCTTCGCCGGCGAC 737	…1014 GTTTAGTGAGCGTTCCCTG-AT-CCAAGGGTCT 1044
C1_23	Allele 1	268 AACCCCCTTGTCGCCGGTCAAAGCCTCTTCG 298	…707 CACAGCCTAATGTGACCGCTTCGCCGGCGAC 737	…1014 GTTTAGTGAGCGTTCCCTG-AT-CCAAGGGTCT 1044
	Allele 2	268 AACCCCCTTGTCGCCGGTCAAAGCCTCTTCG 298	…707 CACAGCCTAATGTGACCGCTTCGCCGGCGAC 737	…1014 GTTTAGTGAGCGTTCCCTG-AT-CCAAGGGTCT 1044
C1_24	Allele 1	268 AACCCCCTTGTCGCCGGTCAAAGCCTCTTCG 298	…707 CACAGCCTAATGTGACCGCTTCGCCGGCGAC 737	…1014 GTTTAGTGAGCGTTCCCTG-AT-CCAAGGGTCT 1044
	Allele 2	268 AACCCCCTTGTCGCCGGTCAAAGCCTCTTCG 298	…707 CACAGCCTAATGTGACCGCTTCGCCGGCGAC 737	…1014 GTTTAGTGAGCGTTCCCTG-AT-CCAAGGGTCT 1044
C1_25	Allele 1	268 AACCCCCTTGTCGCCGGTCAAAGCCTCTTCG 298	…707 CACAGCCTAATGTGACCGCTTCGCCGGCGAC 737	…1014 GTTTAGTGAGCGTTCCCTG-AT-CCAAGGGTCT 1044
	Allele 2	268 AACCCCCTTGTCGCCGGTCAAAGCCTCTTCG 298	…707 CACAGCCTAATGTGACCGCTTCGCCGGCGAC 737	…1014 GTTTAGTGAGCGTTCCCTG-AT-CCAAGGGTCT 1044
C1_26	Allele 1	268 AACCCCCTTGTCGCCGGTCAAAGCCTCTTCG 298	…707 CACAGCCTAATGTGACCGCTTCGCCGGCGAC 737	…1014 GTTTAGTGAGCGTTCCCTG-AT-CCAAGGGTCT 1044
	Allele 2	268 AACCCCCTTGTCGCCGGTCAAAGCCTCTTCG 298	…707 CACAGCCTAATGTGACCGCTTCGCCGGCGAC 737	…1014 GTTTAGTGAGCGTTCCCTG-AT-CCAAGGGTCT 1044
C1_27	Allele 1	268 AACCCCCTTGTCGCCGGTCAAAGCCTCTTCG 298	…707 CACAGCCTAATGTGACCGCTTCGCCGGCGAC 737	…1014 GTTTAGTGAGCGTTCCCTG-AT-CCAAGGGTCT 1044
	Allele 2	268 AACCCCCTTGTCGCCGGTCAAAGCCTCTTCG 298	…707 CACAGCCTAATGTGACCGCTTCGCCGGCGAC 737	…1014 GTTTAGTGAGCGTTCCCTG-AT-CCAAGGGTCT 1044
C1_28	Allele 1	268 AACCCCCTTGTCGCCGGTCAAAGCCTCTTCG 298	…707 CACAGCCTAATGTGACCGCTTCGCCGGCGAC 737	…1014 GTTTAGTGAGCGTTCCCTG-AT-CCAAGGGTCT 1044
	Allele 2	268 AACCCCCTTGTCGCCGGTCAAAGCCTCTTCG 298	…707 CACAGCCTAATGTGACCGCTTCGCCGGCGAC 737	…1014 GTTTAGTGAGCGTTCCCTG-AT-CCAAGGGTCT 1044
C1_29	Allele 1	268 AACCCCCTTGTCGCCGGTCAAAGCCTCTTCG 298	…707 CACAGCCTAATGTGACCGCTTCGCCGGCGAC 737	…1014 GTTTAGTGAGCGTTCCCTG-AT-CCAAGGGTCT 1044
	Allele 2	268 AACCCCCTTGTCGCCGGTCAAAGCCTCTTCG 298	…707 CACAGCCTAATGTGACCGCTTCGCCGGCGAC 737	…1014 GTTTAGTGAGCGTTCCCTG-AT-CCAAGGGTCT 1044
C1_30	Allele 1	268 AACCCCCTTGTCGCCGGTCAAAGCCTCTTCG 298	…707 CACAGCCTAATGTGACCGCTTCGCCGGCGAC 737	…1014 GTTTAGTGAGCGTTCCCTG-AT-CCAAGGGTCT 1044
	Allele 2	268 AACCCCCTTGTCGCCGGTCAAAGCCTCTTCG 298	…707 CACAGCCTAATGTGACCGCTTCGCCGGCGAC 737	…1014 GTTTAGTGAGCGTTCCCTG-AT-CCAAGGGTCT 1044
C1_31	Allele 1	268 AACCCCCTTGTCGCCGGTCAAAGCCTCTTCG 298	…707 CACAGCCTAATGTGACCGCTTCGCCGGCGAC 737	…1014 GTTTAGTGAGCGTTCCCTG-AT-CCAAGGGTCT 1044
	Allele 2	268 AACCCCCTTGTCGCCGGTCAAAGCCTCTTCG 298	…707 CACAGCCTAATGTGACCGCTTCGCCGGCGAC 737	…1014 GTTTAGTGAGCGTTCCCTG-AT-CCAAGGGTCT 1044
C1_32	Allele 1	268 AACCCCCTTGTCGCCGGTCAAAGCCTCTTCG 298	…707 CACAGCCTAATGTGACCGCTTCGCCGGCGAC 737	…1014 GTTTAGTGAGCGTTCCCTG-AT-CCAAGGGTCT 1044
	Allele 2	268 AACCCCCTTGTCGCCGGTCAAAGCCTCTTCG 298	…707 CACAGCCTAATGTGACCGCTTCGCCGGCGAC 737	…1014 GTTTAGTGAGCGTTCCCTG-AT-CCAAGGGTCT 1044
C1_33	Allele 1	268 AACCCCCTTGTCGCCGGTCAAAGCCTCTTCG 298	…707 CACAGCCTAATGTGACCGCTTCGCCGGCGAC 737	…1014 GTTTAGTGAGCGTTCCCTG-AT-CCAAGGGTCT 1044
	Allele 2	268 AACCCCCTTGTCGCCGGTCAAAGCCTCTTCG 298	…707 CACAGCCTAATGTGACCGCTTCGCCGGCGAC 737	…1014 GTTTAGTGAGCGTTCCCTG-AT-CCAAGGGTCT 1044
C1_34	Allele 1	268 AACCCCCTTGTCGCCGGTCAAAGCCTCTTCG 298	…707 CACAGCCTAATGTGACCGCTTCGCCGGCGAC 737	…1014 GTTTAGTGAGCGTTCCCTG-AT-CCAAGGGTCT 1044
	Allele 2	268 AACCCCCTTGTCGCCGGTCAAAGCCTCTTCG 298	…707 CACAGCCTAATGTGACCGCTTCGCCGGCGAC 737	…1014 GTTTAGTGAGCGTTCCCTG-AT-CCAAGGGTCT 1044
C1_35	Allele 1	268 AACCCCCTTGTCGCCGGTCAAAGCCTCTTCG 298	…707 CACAGCCTAATGTGACCGCTTCGCCGGCGAC 737	…1014 GTTTAGTGAGCGTTCCCTG-AT-CCAAGGGTCT 1044
	Allele 2	268 AACCCCCTTGTCGCCGGTCAAAGCCTCTTCG 298	…707 CACAGCCTAATGTGACCGCTTCGCCGGCGAC 737	…1014 GTTTAGTGAGCGTTCCCTG-AT-CCAAGGGTCT 1044
C1_36	Allele 1	268 AACCCCCTTGTCGCCGGTCAAAGCCTCTTCG 298	…707 CACAGCCTAATGTGACCGCTTCGCCGGCGAC 737	…1014 GTTTAGTGAGCGTTCCCTG-AT-CCAAGGGTCT 1044
	Allele 2	268 AACCCCCTTGTCGCCGGTCAAAGCCTCTTCG 298	…707 CACAGCCTAATGTGACCGCTTCGCCGGCGAC 737	…1014 GTTTAGTGAGCGTTCCCTG-AT-CCAAGGGTCT 1044
C1_37	Allele 1	268 AACCCCCTTGTCGCCGGTCAAAGCCTCTTCG 298	…707 CACAGCCTAATGTGACCGCTTCGCCGGCGAC 737	…1014 GTTTAGTGAGCGTTCCCTG-AT-CCAAGGGTCT 1044
	Allele 2	268 AACCCCCTTGTCGCCGGTCAAAGCCTCTTCG 298	…707 CACAGCCTAATGTGACCGCTTCGCCGGCGAC 737	…1014 GTTTAGTGAGCGTTCCCTG-AT-CCAAGGGTCT 1044
C1_38	Allele 1	268 AACCCCCTTGTCGCCGGTCAAAGCCTCTTCG 298	…707 CACAGCCTAATGTGACCGCTTCGCCGGCGAC 737	…1014 GTTTAGTGAGCGTTCCCTG-AT-CCAAGGGTCT 1044
	Allele 2	268 AACCCCCTTGTCGCCGGTCAAAGCCTCTTCG 298	…707 CACAGCCTAATGTGACCGCTTCGCCGGCGAC 737	…1014 GTTTAGTGAGCGTTCCCTG-AT-CCAAGGGTCT 1044
C1_39	Allele 1	268 AACCCCCTTGTCGCCGGTCAAAGCCTCTTCG 298	…707 CACAGCCTAATGTGACCGCTTCGCCGGCGAC 737	…1014 GTTTAGTGAGCGTTCCCTG-AT-CCAAGGGTCT 1044
	Allele 2	268 AACCCCCTTGTCGCCGGTCAAAGCCTCTTCG 298	…707 CACAGCCTAATGTGACCGCTTCGCCGGCGAC 737	…1014 GTTTAGTGAGCGTTCCCTG-AT-CCAAGGGTCT 1044
C1_40	Allele 1	268 AACCCCCTTGTCGCCGGTCAAAGCCTCTTCG 298	…707 CACAGCCTAATGTGACCGCTTCGCCGGCGAC 737	…1014 GTTTAGTGAGCGTTCCCTG-AT-CCAAGGGTCT 1044
	Allele 2	268 AACCCCCTTGTCGCCGGTCAAAGCCTCTTCG 298	…707 CACAGCCTAATGTGACCGCTTCGCCGGCGAC 737	…1014 GTTTAGTGAGCGTTCCCTG-AT-CCAAGGGTCT 1044
C1_41	Allele 1	268 AACCCCCTTGTCGCCGGTCAAAGCCTCTTCG 298	…707 CACAGCCTAATGTGACCGCTTCGCCGGCGAC 737	…1014 GTTTAGTGAGCGTTCCCTG-AT-CCAAGGGTCT 1044
	Allele 2	268 AACCCCCTTGTCGCCGGTCAAAGCCTCTTCG 298	…707 CACAGCCTAATGTGACCGCTTCGCCGGCGAC 737	…1014 GTTTAGTGAGCGTTCCCTG-AT-CCAAGGGTCT 1044
C1_42	Allele 1	268 AACCCCCTTGTCGCCGGTCAAAGCCTCTTCG 298	…707 CACAGCCTAATGTGACCGCTTCGCCGGCGAC 737	…1014 GTTTAGTGAGCGTTCCCTG-AT-CCAAGGGTCT 1044
	Allele 2	268 AACCCCCTTGTCGCCGGTCAAAGCCTCTTCG 298	…707 CACAGCCTAATGTGACCGCTTCGCCGGCGAC 737	…1014 GTTTAGTGAGCGTTCCCTG-AT-CCAAGGGTCT 1044

WT	Allele 1	268	AACCCCCTTGTCGCCGGTCAAAGCCTCTTCG	298…707	CACAGCCTAATGTGACCGCTTCGCCGGCGAC	737…1014	GTTTAGTGAGCGTTCCCTG-AT-CCAAGGGTCT	1044
	Allele 2	268	AACCCCCTTGTCGCCGGTCAAAGCCTCTTCG	298…707	CACAGCCTAATGTGACCGCTTCGCCGGCGAC	737…1014	GTTTAGTGAGCGTTCCCTG-AT-CCAAGGGTCT	1044
C1_43	Allele 1	268	AACCCCCTTGTCGCCGGTCAAAGCCTCTTCG	298…707	CACAGCCTAATGTGACCGCTTCGCCGGCGAC	737…1014	GTTTAGTGAGCGTTCCCTG-ATTCCAAGGGTCT	1045
	Allele 2	268	AACCCCCTTGTCGCCGGTCAAAGCCTCTTCG	298…707	CACAGCCTAATGTGACCGCTTCGCCGGCGAC	737…1014	GTTTAGTGAGCGTTCCCTG-A--CCAAGGGTCT	1043
C1_44	Allele 1	268	AACCCCCTTGTCGCCGGTCAAAGCCTCTTCG	298…707	CACAGCCTAATGTGACCGCTTCGCCGGCGAC	737…1014	GTTTAGTGAGCGTTCCCTG-AT-CCAAGGGTCT	1044
	Allele 2	268	AACCCCCTTGTCGCCGGTCAAAGCCTCTTCG	298…707	CACAGCCTAATGTGACCGCTTCGCCGGCGAC	737…1014	GTTTAGTGAGCGTTCCCTG-AT-CCAAGGGTCT	1044
C1_45	Allele 1	268	AACCCCCTTGTCGCCGGTCAAAGCCTCTTCG	298…707	CACAGCCTAATGTGACCGCTTCGCCGGCGAC	737…1014	GTTTAGTGAGCGTTCCCTG-AT-CCAAGGGTCT	1044
	Allele 2	268	AACCCCCTTGTCGCCGGTCAAAGCCTCTTCG	298…707	CACAGCCTAATGTGACCGCTTCGCCGGCGAC	737…1014	GTTTAGTGAGCGTTCCCTG-AT-CCAAGGGTCT	1044
C1_46	Allele 1	268	AACCCCCTTGTCGCCGGTCAAAGCCTCTTCG	298…707	CACAGCCTAATGTGACCGCTTCGCCGGCGAC	737…1014	GTTTAGTGAGCGTTCCCTG-AT-CCAAGGGTCT	1044
	Allele 2	268	AACCCCCTTGTCGCCGGTCAAAGCCTCTTCG	298…707	CACAGCCTAATGTGACCGCTTCGCCGGCGAC	737…1014	GTTTAGTGAGCGTTCCCTG-A--CCAAGGGTCT	1043
C1_47	Allele 1	268	AACCCCCTTGTCGCCGGTCAAAGCCTCTTCG	298…707	CACAGCCTAATGTGACCGCTTCGCCGGCGAC	737…1014	GTTTAGTGAGCGTTCCCTG-AT-CCAAGGGTCT	1044
	Allele 2	268	AACCCCCTTGTCGCCGGTCAAAGCCTCTTCG	298…707	CACAGCCTAATGTGACCGCTTCGCCGGCGAC	737…1014	GTTTAGTGAGCGTTCCCTG-AT-CCAAGGGTCT	1044
C1_48	Allele 1	268	AACCCCCTTGTCGCCGGTCAAAGCCTCTTCG	298…707	CACAGCCTAATGTGACCGCTTCGCCGGCGAC	737…1014	GTTTAGTGAGCGTTCCCTG-AT-CCAAGGGTCT	1044
	Allele 2	268	AACCCCCTTGTCGCCGGTCAAAGCCTCTTCG	298…707	CACAGCCTAATGTGACCGCTTCGCCGGCGAC	737…1014	GTTTAGTGAGCGTTCCCTG-AT-CCAAGGGTCT	1044
C1_49	Allele 1	268	AACCCCCTTGTCGCCGGTCAAAGCCTCTTCG	298…707	CACAGCCTAATGTGACCGCTTCGCCGGCGAC	737…1014	GTTTAGTGAGCGTTCCCTG-AT-CCAAGGGTCT	1044
	Allele 2	268	AACCCCCTTGTCGCCGGTCAAAGCCTCTTCG	298…707	CACAGCCTAATGTGACCGCTTCGCCGGCGAC	737…1014	GTTTAGTGAGCGTTCCCTG-AT-CCAAGGGTCT	1044
C1_50	Allele 1	268	AACCCCCTTGTCGCCGGTCAAAGCCTCTTCG	298…707	CACAGCCTAATGTGACCGCTTCGCCGGCGAC	737…1014	GTTTAGTGAGCGTTCCCTG-AT-CCAAGGGTCT	1044
	Allele 2	268	AACCCCCTTGTCGCCGGTCAAAGCCTCTTCG	298…707	CACAGCCTAATGTGACCGCTTCGCCGGCGAC	737…1014	GTTTAGTGAGCGTTCCCTG-AT-CCAAGGGTCT	1044
C1_51	Allele 1	268	AACCCCCTTGTCGCCGGTCAAAGCCTCTTCG	298…707	CACAGCCTAATGTGACCGCTTCGCCGGCGAC	737…1014	GTTTAGTGAGCGTTCCCTG-AT-CCAAGGGTCT	1044
	Allele 2	268	AACCCCCTTGTCGCCGGTCAAAGCCTCTTCG	298…707	CACAGCCTAATGTGACCGCTTCGCCGGCGAC	737…1014	GTTTAGTGAGCGTTCCCTG-AT-CCAAGGGTCT	1044
C1_52	Allele 1	268	AACCCCCTTGTCGCCGGTCAAAGCCTCTTCG	298…707	CACAGCCTAATGTGACCGCTTCGCCGGCGAC	737…1014	GTTTAGTGAGCGTTCCCTG-AT-CCAAGGGTCT	1044
	Allele 2	268	AACCCCCTTGTCGCCGGTCAAAGCCTCTTCG	298…707	CACAGCCTAATGTGACCGCTTCGCCGGCGAC	737…1014	GTTTAGTGAGCGTTCCCTG-AT-CCAAGGGTCT	1044
C1_53	Allele 1	268	AACCCCCTTGTCGCCGGTCAAAGCCTCTTCG	298…707	CACAGCCTAATGTGACCGCTTCGCCGGCGAC	737…1014	GTTTAGTGAGCGTTCCCTG-AT-CCAAGGGTCT	1044
	Allele 2	268	AACCCCCTTGTCGCCGGTCAAAGCCTCTTCG	298…707	CACAGCCTAATGTGACCGCTTCGCCGGCGAC	737…1014	GTTTAGTGAGCGTTCCCTG-AT-CCAAGGGTCT	1044
C1_54	Allele 1	268	AACCCCCTTGTCGCCGGTCAAAGCCTCTTCG	298…707	CACAGCCTAATGTGACCGCTTCGCCGGCGAC	737…1014	GTTTAGTGAGCGTTCCCTG-AT-CCAAGGGTCT	1044
	Allele 2	268	AACCCCCTTGTCGCCGGTCAAAGCCTCTTCG	298…707	CACAGCCTAATGTGACCGCTTCGCCGGCGAC	737…1014	GTTTAGTGAGCGTTCCCTG-AT-CCAAGGGTCT	1044
C1_55	Allele 1	268	AACCCCCTTGTCGCCGGTCAAAGCCTCTTCG	298…707	CACAGCCTAATGTGACCGCTTCGCCGGCGAC	737…1014	GTTTAGTGAGCGTTCCCTG-AT-CCAAGGGTCT	1044
	Allele 2	268	AACCCCCTTGTCGCCGGTCAAAGCCTCTTCG	298…707	CACAGCCTAATGTGACCGCTTCGCCGGCGAC	737…1014	GTTTAGTGAGCGTTCCCTG-AT-CCAAGGGTCT	1044
C1_56	Allele 1	268	AACCCCCTTGTCGCCGGTCAAAGCCTCTTCG	298…707	CACAGCCTAATGTGACCGCTTCGCCGGCGAC	737…1014	GTTTAGTGAGCGTTCCCTG-AT-CCAAGGGTCT	1044
	Allele 2	268	AACCCCCTTGTCGCCGGTCAAAGCCTCTTCG	298…707	CACAGCCTAATGTGACCGCTTCGCCGGCGAC	737…1014	GTTTAGTGAGCGTTCCCTG-AT-CCAAGGGTCT	1044
C1_57	Allele 1	268	AACCCCCTTGTCGCCGGTCAAAGCCTCTTCG	298…707	CACAGCCTAATGTGACCGCTTCGCCGGCGAC	737…1014	GTTTAGTGAGCGTTCCCTG-AT-CCAAGGGTCT	1044
	Allele 2	268	AACCCCCTTGTCGCCGGTCAAAGCCTCTTCG	298…707	CACAGCCTAATGTGACCGCTTCGCCGGCGAC	737…1014	GTTTAGTGAGCGTTCCCTG-AT-CCAAGGGTCT	1044
C1_58	Allele 1	268	AACCCCCTTGTCGCCGGTCAAAGCCTCTTCG	298…707	CACAGCCTAATGTGACCGCTTCGCCGGCGAC	737…1014	GTTTAGTGAGCGTTCCCTG-AT-CCAAGGGTCT	1044
	Allele 2	268	AACCCCCTTGTCGCCGGTCAAAGCCTCTTCG	298…707	CACAGCCTAATGTGACCGCTTCGCCGGCGAC	737…1014	GTTTAGTGAGCGTTCCCTG-AT-CCAAGGGTCT	1044
C1_59	Allele 1	268	AACCCCCTTGTCGCCGGTCAAAGCCTCTTCG	298…707	CACAGCCTAATGTGACCGCTTCGCCGGCGAC	737…1014	GTTTAGTGAGCGTTCCCTG-AT-CCAAGGGTCT	1044
	Allele 2	268	AACCCCCTTGTCGCCGGTCAAAGCCTCTTCG	298…707	CACAGCCTAATGTGACCGCTTCGCCGGCGAC	737…1014	GTTTAGTGAGCGTTCCCTG-AT-CCAAGGGTCT	1044
C1_60	Allele 1	268	AACCCCCTTGTCGCCGGTCAAAGCCTCTTCG	298…707	CACAGCCTAATGTGACCGCTTCGCCGGCGAC	737…1014	GTTTAGTGAGCGTTCCCTG-AT-CCAAGGGTCT	1044
	Allele 2	268	AACCCCCTTGTCGCCGGTCAAAGCCTCTTCG	298…707	CACAGCCTAATGTGACCGCTTCGCCGGCGAC	737…1014	GTTTAGTGAGCGTTCCCTG-AT-CCAAGGGTCT	1044
C1_61	Allele 1	268	AACCCCCTTGTCGCCGGTCAAAGCCTCTTCG	298…707	CACAGCCTAATGTGACCGCTTCGCCGGCGAC	737…1014	GTTTAGTGAGCGTTCCCTG-AT-CCAAGGGTCT	1044
	Allele 2	268	AACCCCCTTGTCGCCGGTCAAAGCCTCTTCG	298…707	CACAGCCTAATGTGACCGCTTCGCCGGCGAC	737…1014	GTTTAGTGAGCGTTCCCTG-AT-CCAAGGGTCT	1044
C1_62	Allele 1	268	AACCCCCTTGTCGCCGGTCAAAGCCTCTTCG	298…707	CACAGCCTAATGTGACCGCTTCGCCGGCGAC	737…1014	GTTTAGTGAGCGTTCCCTG-AT-CCAAGGGTCT	1044
	Allele 2	268	AACCCCCTTGTCGCCGGTCAAAGCCTCTTCG	298…707	CACAGCCTAATGTGACCGCTTCGCCGGCGAC	737…1014	GTTTAGTGAGCGTTCCCTG-AT-CCAAGGGTCT	1044
C1_63	Allele 1	268	AACCCCCTTGTCGCCGGTCAAAGCCTCTTCG	298…707	CACAGCCTAATGTGACCGCTTCGCCGGCGAC	737…1014	GTTTAGTGAGCGTTCCCTG-AT-CCAAGGGTCT	1044
	Allele 2	268	AACCCCCTTGTCGCCGGTCAAAGCCTCTTCG	298…707	CACAGCCTAATGTGACCGCTTCGCCGGCGAC	737…1014	GTTTAGTGAGCGTTCCCTG-AT-CCAAGGGTCT	1044
C1_64	Allele 1	268	AACCCCCTTGTCGCCGGTCAAAGCCTCTTCG	298…707	CACAGCCTAATGTGACCGCTTCGCCGGCGAC	737…1014	GTTTAGTGAGCGTTCCCTG-AT-CCAAGGGTCT	1044
	Allele 2	268	AACCCCCTTGTCGCCGGTCAAAGCCTCTTCG	298…707	CACAGCCTAATGTGACCGCTTCGCCGGCGAC	737…1014	GTTTAGTGAGCGTTCCCTG-AT-CCAAGGGTCT	1044
C1_65	Allele 1	268	AACCCCCTTGTCGCCGGTCAAAGCCTCTTCG	298…707	CACAGCCTAATGTGACCGCTTCGCCGGCGAC	737…1014	GTTTAGTGAGCGTTCCCTG-AT-CCAAGGGTCT	1044
	Allele 2	268	AACCCCCTTGTCGCCGGTCAAAGCCTCTTCG	298…707	CACAGCCTAATGTGACCGCTTCGCCGGCGAC	737…1014	GTTTAGTGAGCGTTCCCTG-AT-CCAAGGGTCT	1044
C1_66	Allele 1	268	AACCCCCTTGTCGCCGGTCAAAGCCTCTTCG	298…707	CACAGCCTAATGTGACCGCTTCGCCGGCGAC	737…1014	GTTTAGTGAGCGTTCCCTG-AT-CCAAGGGTCT	1044
	Allele 2	268	AACCCCCTTGTCGCCGGTCAAAGCCTCTTCG	298…707	CACAGCCTAATGTGACCGCTTCGCCGGCGAC	737…1014	GTTTAGTGAGCGTTCCCTG-AT-CCAAGGGTCT	1044
C1_67	Allele 1	268	AACCCCCTTGTCGCCGGTCAAAGCCTCTTCG	298…707	CACAGCCTAATGTGACCGCTTCGCCGGCGAC	737…1014	GTTTAGTGAGCGTTCCCTG-AT-CCAAGGGTCT	1044
	Allele 2	268	AACCCCCTTGTCGCCGGTCAAAGCCTCTTCG	298…707	CACAGCCTAATGTGACCGCTTCGCCGGCGAC	737…1014	GTTTAGTGAGCGTTCCCTG-AT-CCAAGGGTCT	1044
C1_68	Allele 1	268	AACCCCCTTGTCGCCGGTCAAAGCCTCTTCG	298…707	CACAGCCTAATGTGACCGCTTCGCCGGCGAC	737…1014	GTTTAGTGAGCGTTCCCTG-AT-CCAAGGGTCT	1044
	Allele 2	268	AACCCCCTTGTCGCCGGTCAAAGCCTCTTCG	298…707	CACAGCCTAATGTGACCGCTTCGCCGGCGAC	737…1014	GTTTAGTGAGCGTTCCCTG-AT-CCAAGGGTCT	1044
C1_69	Allele 1	268	AACCCCCTTGTCGCCGGTCAAAGCCTCTTCG	298…707	CACAGCCTAATGTGACCGCTTCGCCGGCGAC	737…1014	GTTTAGTGAGCGTTCCCTG-AT-CCAAGGGTCT	1044
	Allele 2	268	AACCCCCTTGTCGCCGGTCAAAGCCTCTTCG	298…707	CACAGCCTAATGTGACCGCTTCGCCGGCGAC	737…1014	GTTTAGTGAGCGTTCCCTG-AT-CCAAGGGTCT	1044
C1_70	Allele 1	268	AACCCCCTTGTCGCCGGTCAAAGCCTCTTCG	298…707	CACAGCCTAATGTGACCGCTTCGCCGGCGAC	737…1014	GTTTAGTGAGCGTTCCCTG-AT-CCAAGGGTCT	1044
	Allele 2	268	AACCCCCTTGTCGCCGGTCAAAGCCTCTTCG	298…707	CACAGCCTAATGTGACCGCTTCGCCGGCGAC	737…1014	GTTTAGTGAGCGTTCCCTG-AT-CCAAGGGTCT	1044
C1_71	Allele 1	268	AACCCCCTTGTCGCCGGTCAAAGCCTCTTCG	298…707	CACAGCCTAATGTGACCGCTTCGCCGGCGAC	737…1014	GTTTAGTGAGCGTTCCCTG-AT-CCAAGGGTCT	1044
	Allele 2	268	AACCCCCTTGTCGCCGGTCAAAGCCTCTTCG	298…707	CACAGCCTAATGTGACCGCTTCGCCGGCGAC	737…1014	GTTTAGTGAGCGTTCCCTG-AT-CCAAGGGTCT	1044
C1_72	Allele 1	268	AACCCCCTTGTCGCCGGTCAAAGCCTCTTCG	298…707	CACAGCCTAATGTGACCGCTTCGCCGGCGAC	737…1014	GTTTAGTGAGCGTTCCCTG-AT-CCAAGGGTCT	1044
	Allele 2	268	AACCCCCTTGTCGCCGGTCAAAGCCTCTTCG	298…707	CACAGCCTAATGTGACCGCTTCGCCGGCGAC	737…1014	GTTTAGTGAGCGTTCCCTG-AT-CCAAGGGTCT	1044
C1_73	Allele 1	268	AACCCCCTTGTCGCCGGTCAAAGCCTCTTCG	298…707	CACAGCCTAATGTGACCGCTTCGCCGGCGAC	737…1014	GTTTAGTGAGCGTTCCCTG-AT-CCAAGGGTCT	1044
	Allele 2	268	AACCCCCTTGTCGCCGGTCAAAGCCTCTTCG	298…707	CACAGCCTAATGTGACCGCTTCGCCGGCGAC	737…1014	GTTTAGTGAGCGTTCCCTG-AT-CCAAGGGTCT	1044
C1_74	Allele 1	268	AACCCCCTTGTCGCCGGTCAAAGCCTCTTCG	298…707	CACAGCCTAATGTGACCGCTTCGCCGGCGAC	737…1014	GTTTAGTGAGCGTTCCCTG-AT-CCAAGGGTCT	1044
	Allele 2	268	AACCCCCTTGTCGCCGGTCAAAGCCTCTTCG	298…707	CACAGCCTAATGTGACCGCTTCGCCGGCGAC	737…1014	GTTTAGTGAGCGTTCCCTG-AT-CCAAGGGTCT	1044
C1_75	Allele 1	268	AACCCCCTTGTCGCCGGTCAAAGCCTCTTCG	298…707	CACAGCCTAATGTGACCGCTTCGCCGGCGAC	737…1014	GTTTAGTGAGCGTTCCCTG-AT-CCAAGGGTCT	1044
	Allele 2	268	AACCCCCTTGTCGCCGGTCAAAGCCTCTTCG	298…707	CACAGCCTAATGTGACCGCTTCGCCGGCGAC	737…1014	GTTTAGTGAGCGTTCCCTG-AT-CCAAGGGTCT	1044
C1_76	Allele 1	268	AACCCCCTTGTCGCCGGTCAAAGCCTCTTCG	298…707	CACAGCCTAATGTGACCGCTTCGCCGGCGAC	737…1014	GTTTAGTGAGCGTTCCCTG-AT-CCAAGGGTCT	1044
	Allele 2	268	AACCCCCTTGTCGCCGGTCAAAGCCTCTTCG	298…707	CACAGCCTAATGTGACCGCTTCGCCGGCGAC	737…1014	GTTTAGTGAGCGTTCCCTG-AT-CCAAGGGTCT	1044
C1_77	Allele 1	268	AACCCCCTTGTCGCCGGTCAAAGCCTCTTCG	298…707	CACAGCCTAATGTGACCGCTTCGCCGGCGAC	737…1014	GTTTAGTGAGCGTTCCCTG-AT-CCAAGGGTCT	1044
	Allele 2	268	AACCCCCTTGTCGCCGGTCAAAGCCTCTTCG	298…707	CACAGCCTAATGTGACCGCTTCGCCGGCGAC	737…1014	GTTTAGTGAGCGTTCCCTG-AT-CCAAGGGTCT	1044
C1_78	Allele 1	268	AACCCCCTTGTCGCCGGTCAAAGCCTCTTCG	298…707	CACAGCCTAATGTGACCGCTTCGCCGGCGAC	737…1014	GTTTAGTGAGCGTTCCCTG-AT-CCAAGGGTCT	1044
	Allele 2	268	AACCCCCTTGTCGCCGGTCAAAGCCTCTTCG	298…707	CACAGCCTAATGTGACCGCTTCGCCGGCGAC	737…1014	GTTTAGTGAGCGTTCCCTG-AT-CCAAGGGTCT	1044
C1_79	Allele 1	268	AACCCCCTTGTCGCCGGTCAAAGCCTCTTCG	298…707	CACAGCCTAATGTGACCGCTTCGCCGGCGAC	737…1014	GTTTAGTGAGCGTTCCCTG-AT-CCAAGGGTCT	1044
	Allele 2	268	AACCCCCTTGTCGCCGGTCAAAGCCTCTTCG	298…707	CACAGCCTAATGTGACCGCTTCGCCGGCGAC	737…1014	GTTTAGTGAGCGTTCCCTG-AT-CCAAGGGTCT	1044
C1_80	Allele 1	268	AACCCCCTTGTCGCCGGTCAAAGCCTCTTCG	298…707	CACAGCCTAATGTGACCGCTTCGCCGGCGAC	737…1014	GTTTAGTGAGCGTTCCCTG-AT-CCAAGGGTCT	1044
	Allele 2	268	AACCCCCTTGTCGCCGGTCAAAGCCTCTTCG	298…707	CACAGCCTAATGTGACCGCTTCGCCGGCGAC	737…1014	GTTTAGTGAGCGTTCCCTG-AT-CCAAGGGTCT	1044
C1_81	Allele 1	268	AACCCCCTTGTCGCCGGTCAAAGCCTCTTCG	298…707	CACAGCCTAATGTGACCGCTTCGCCGGCGAC	737…1014	GTTTAGTGAGCGTTCCCTG-AT-CCAAGGGTCT	1044
	Allele 2	268	AACCCCCTTGTCGCCGGTCAAAGCCTCTTCG	298…707	CACAGCCTAATGTGACCGCTTCGCCGGCGAC	737…1014	GTTTAGTGAGCGTTCCCTG-AT-CCAAGGGTCT	1044
C1_82	Allele 1	268	AACCCCCTTGTCGCCGGTCAAAGCCTCTTCG	298…707	CACAGCCTAATGTGACCGCTTCGCCGGCGAC	737…1014	GTTTAGTGAGCGTTCCCTG-AT-CCAAGGGTCT	1044
	Allele 2	268	AACCCCCTTGTCGCCGGTCAAAGCCTCTTCG	298…707	CACAGCCTAATGTGACCGCTTCGCCGGCGAC	737…1014	GTTTAGTGAGCGTTCCCTG-AT-CCAAGGGTCT	1044
C1_83	Allele 1	268	AACCCCCTTGTCGCCGGTCAAAGCCTCTTCG	298…707	CACAGCCTAATGTGACCGCTTCGCCGGCGAC	737…1014	GTTTAGTGAGCGTTCCCTG-AT-CCAAGGGTCT	1044
	Allele 2	268	AACCCCCTTGTCGCCGGTCAAAGCCTCTTCG	298…707	CACAGCCTAATGTGACCGCTTCGCCGGCGAC	737…1014	GTTTAGTGAGCGTTCCCTG-AT-CCAAGGGTCT	1044
C1_84	Allele 1	268	AACCCCCTTGTCGCCGGTCAAAGCCTCTTCG	298…707	CACAGCCTAATGTGACCGCTTCGCCGGCGAC	737…1014	GTTTAGTGAGCGTTCCCTG-AT-CCAAGGGTCT	1044
	Allele 2	268	AACCCCCTTGTCGCCGGTCAAAGCCTCTTCG	298…707	CACAGCCTAATGTGACCGCTTCGCCGGCGAC	737…1014	GTTTAGTGAGCGTTCCCTG-AT-CCAAGGGTCT	1044
C1_85	Allele 1	268	AACCCCCTTGTCGCCGGTCAAAGCCTCTTCG	298…707	CACAGCCTAATGTGACCGCTTCGCCGGCGAC	737…1014	GTTTAGTGAGCGTTCCCTG-AT-CCAAGGGTCT	1044
	Allele 2	268	AACCCCCTTGTCGCCGGTCAAAGCCTCTTCG	298…707	CACAGCCTAATGTGACCGCTTCGCCGGCGAC	737…1014	GTTTAGTGAGCGTTCCCTG-AT-CCAAGGGTCT	1044

Figure S5. Alignment of the 85 PCR products sequenced against wild-type sequence in the region of the 3 sgRNA for transgenic lines transformed with construct 1 (*p35S*::*hCas9*). Wild-type sgRNA sequence is highlighted in grey and all insertions or deletions are highlighted in red. Each allele is represented for all plantlets to highlight monoallelic or biallelic mutations.

```
                                   sgRNA 1                                          sgRNA 2                                              sgRNA 3
WT    Allele 1 268 AACCCCCTTG--TCGCCGGTCAAAGCCTCTTCG 298…707 CACAGCCTAATGTGACCGCTT-CGCCGGCGAC 737…1014 GTTTAGTGAGCGTTCCCTGAT--CCAAGGGTCT 1044
      Allele 2 268 AACCCCCTTG--TCGCCGGTCAAAGCCTCTTCG 298…707 CACAGCCTAATGTGACCGCTT-CGCCGGCGAC 737…1014 GTTTAGTGAGCGTTCCCTGAT--CCAAGGGTCT 1044
C2_1  Allele 1 268 AACCCCCTTGA-TCGCCGGTCAAAGCCTCTTCG 299…708 CACAGCCTAATGTGACCGCTT-CGCCGGCGAC 738…1015 GTTTAGTGAGCGTTCCCTGATA-CCAAGGGTCT 1046
      Allele 2 268 AACCCCCTTGA-TCGCCGGTCAAAGCCTCTTCG 299…708 CACAGCCTAATGTGACCGCTT-CGCCGGCGAC 738…1015 GTTTAGTGAGCGTTCCCTGA---CCAAGGGTCT 1044
C2_2  Allele 1 268 AACCCCCTTGGTTCGCCGGTCAAAGCCTCTTCG 300…709 CACAGCCTAATGTGACCGCTT-CGCCGGCGAC 739…1016 GTTTAGTGAGCGTTCCCTGATA-CCAAGGGTCT 1047
      Allele 2 268 AACCCCCTTGGTTCGCCGGTCAAAGCCTCTTCG 300…709 CACAGCCTAATGTGACCGCTT-CGCCGGCGAC 739…1016 GTTTAGTGAGCGTTCCCTGA---CCAAGGGTCT 1045
C2_3  Allele 1 268 AACCCCCTTGGTTCGCCGGTCAAAGCCTCTTCG 300…709 CACAGCCTAATGTGACCGCTT-CGCCGGCGAC 739…1016 GTTTAGTGAGCGTTCCCTGATA-CCAAGGGTCT 1047
      Allele 2 268 AACCCCCTTGGTTCGCCGGTCAAAGCCTCTTCG 300…709 CACAGCCTAATGTGACCGCTT-CGCCGGCGAC 739…1016 GTTTAGTGAGCGTTCCCTGA---CCAAGGGTCT 1045
C2_4  Allele 1 268 AACCCCCTTG--TCGCCGGTCAAAGCCTCTTCG 298…707 CACAGCCTAATGTGACCGCTT-CGCCGGCGAC 737…1014 GTTTAGTGAGCGTTCCCTGATA-CCAAGGGTCT 1045
      Allele 2 268 AACCCCCTTG--TCGCCGGTCAAAGCCTCTTCG 298…707 CACAGCCTAATGTGACCGCTT-CGCCGGCGAC 737…1014 GTTTAGTGAGCGTTCCCTGA---CCAAGGGTCT 1043
C2_5  Allele 1 268 AACCCCCTTGA-TCGCCGGTCAAAGCCTCTTCG 299…708 CACAGCCTAATGTGACCGCTT-CGCCGGCGAC 738…1015 GTTTAGTGAGCGTTCCCTGATA-CCAAGGGTCT 1046
      Allele 2 268 AACCCCCTTGA-TCGCCGGTCAAAGCCTCTTCG 299…708 CACAGCCTAATGTGACCGCTT-CGCCGGCGAC 738…1015 GTTTAGTGAGCGTTCCCTGA---CCAAGGGTCT 1044
C2_6  Allele 1 268 AACCCCCTTGA-TCGCCGGTCAAAGCCTCTTCG 299…708 CACAGCCTAATGTGACCGCTT-CGCCGGCGAC 738…1015 GTTTAGTGAGCGTTCCCTGATA-CCAAGGGTCT 1046
      Allele 2 268 AACCCCCTTGA-TCGCCGGTCAAAGCCTCTTCG 299…708 CACAGCCTAATGTGACCGCTT-CGCCGGCGAC 738…1015 GTTTAGTGAGCGTTCCCTGA---CCAAGGGTCT 1044
C2_7  Allele 1 268 AACCCCCTTG--------GTCAAAGCCTCTTCG 292…701 CACAGCCTAATGTGACCGCTT-CGCCGGCGAC 731…1008 GTTTAGTGAGCGTTCCCTGATA-CCAAGGGTCT 1039
      Allele 2 268 AACCCCCTTG--------GTCAAAGCCTCTTCG 292…701 CACAGCCTAATGTGACCGCTT-CGCCGGCGAC 731…1008 GTTTAGTGAGCGTTCCCTGA---CCAAGGGTCT 1037
C2_8  Allele 1 268 AACCCCCTTG--TCGCCGGTCAAAGCCTCTTCG 298…707 CACAGCCTAATGTGACCGCTT-CGCCGGCGAC 737…1014 GTTTAGTGAGCGTTCCCTGATA-CCAAGGGTCT 1045
      Allele 2 268 AACCCCCTTG--TCGCCGGTCAAAGCCTCTTCG 298…707 CACAGCCTAATGTGACCGCTT-CGCCGGCGAC 737…1014 GTTTAGTGAGCGTTCCCTGA---CCAAGGGTCT 1043
C2_9  Allele 1 268 AACCCCCTTGA-TCGCCGGTCAAAGCCTCTTCG 299…708 CACAGCCTAATGTGACCGCTT-CGCCGGCGAC 738…1015 GTTTAGTGAGCGTTCCCTGATA-CCAAGGGTCT 1046
      Allele 2 268 AACCCCCTTGA-TCGCCGGTCAAAGCCTCTTCG 299…708 CACAGCCTAATGTGACCGCTT-CGCCGGCGAC 738…1015 GTTTAGTGAGCGTTCCCTGA---CCAAGGGTCT 1044
C2_10 Allele 1 268 AACCCCCTTG--TCGCCGGTCAAAGCCTCTTCG 298…707 CACAGCCTAATGTGACCGCTT-CGCCGGCGAC 737…1014 GTTTAGTGAGCGTTCCCTGATA-CCAAGGGTCT 1045
      Allele 2 268 AACCCCCTTG--TCGCCGGTCAAAGCCTCTTCG 298…707 CACAGCCTAATGTGACCGCTT-CGCCGGCGAC 737…1014 GTTTAGTGAGCGTTCCCTGA---CCAAGGGTCT 1043
C2_11 Allele 1 268 AACCCCCTTG--TCGCCGGTCAAAGCCTCTTCG 298…707 CACAGCCTAATGTGACCGCTT-CGCCGGCGAC 737…1014 GTTTAGTGAGCGTTCCCTGATA-CCAAGGGTCT 1045
      Allele 2 268 AACCCCCTTG--TCGCCGGTCAAAGCCTCTTCG 298…707 CACAGCCTAATGTGACCGCTT-CGCCGGCGAC 737…1014 GTTTAGTGAGCGTTCCCTGA---CCAAGGGTCT 1043
C2_12 Allele 1 268 AACCCCCTTG--TCGCCGGTCAAAGCCTCTTCG 298…707 CACAGCCTAATGTGACCGCTT-CGCCGGCGAC 737…1014 GTTTAGTGAGCGTTCCCTGATTACCAAGGGTCT 1046
      Allele 2 268 AACCCCCTTG--TCGCCGGTCAAAGCCTCTTCG 298…707 CACAGCCTAATGTGACCGCTT-CGCCGGCGAC 737…1014 GTTTAGTGAGCGTTCCCTGA---CCAAGGGTCT 1043
C2_13 Allele 1 268 AACCCCCTTGGTTCGCCGGTCAAAGCCTCTTCG 300…709 CACAGCCTAATGTGACCGCTT-CGCCGGCGAC 739…1016 GTTTAGTGAGCGTTCCCTGATA-CCAAGGGTCT 1047
      Allele 2 268 AACCCCCTTGGTTCGCCGGTCAAAGCCTCTTCG 300…709 CACAGCCTAATGTGACCGCTT-CGCCGGCGAC 739…1016 GTTTAGTGAGCGTTCCCTGA---CCAAGGGTCT 1045
C2_14 Allele 1 268 AACCCCCTTGA-TCGCCGGTCAAAGCCTCTTCG 299…708 CACAGCCTAATGTGACCGCTT-CGCCGGCGAC 738…1015 GTTTAGTGAGCGTTCCCTGATA-CCAAGGGTCT 1046
      Allele 2 268 AACCCCCTTGA-TCGCCGGTCAAAGCCTCTTCG 299…708 CACAGCCTAATGTGACCGCTT-CGCCGGCGAC 738…1015 GTTTAGTGAGCGTTCCCTGA---CCAAGGGTCT 1044
C2_15 Allele 1 268 AACCCCCTTGA-TCGCCGGTCAAAGCCTCTTCG 299…708 CACAGCCTAATGTGACCGCTT-CGCCGGCGAC 738…1015 GTTTAGTGAGCGTTCCCTGATA-CCAAGGGTCT 1046
      Allele 2 268 AACCCCCTTGA-TCGCCGGTCAAAGCCTCTTCG 299…708 CACAGCCTAATGTGACCGCTT-CGCCGGCGAC 738…1015 GTTTAGTGAGCGTTCCCTGA---CCAAGGGTCT 1044
C2_16 Allele 1 268 AACCCCCTTGA-TCGCCGGTCAAAGCCTCTTCG 299…708 CACAGCCTAATGTGACCGCTT-CGCCGGCGAC 738…1015 GTTTAGTGAGCGTTCCCTGATA-CCAAGGGTCT 1046
      Allele 2 268 AACCCCCTTGA-TCGCCGGTCAAAGCCTCTTCG 299…708 CACAGCCTAATGTGACCGCTT-CGCCGGCGAC 738…1015 GTTTAGTGAGCGTTCCCTGA---CCAAGGGTCT 1044
C2_17 Allele 1 268 AACCCCCTTG--TCGCCGGTCAAAGCCTCTTCG 298…707 CACAGCCTAATGTGACCGCTT-CGCCGGCGAC 737…1014 GTTTAGTGAGCGTTCCCTGA---CCAAGGGTCT 1043
      Allele 2 268 AACCCCCTTG--TCGCCGGTCAAAGCCTCTTCG 298…707 CACAGCCTAATGTGACCGCTT-CGCCGGCGAC 737…1014 GTTTAGTGAGCGTTCCCTGA---CCAAGGGTCT 1043
C2_18 Allele 1 268 AACCCCCTTGGTTCGCCGGTCAAAGCCTCTTCG 300…709 CACAGCCTAATGTGACCGCTT-CGCCGGCGAC 739…1016 GTTTAGTGAGCGTTCCCTGATA-CCAAGGGTCT 1047
      Allele 2 268 AACCCCCTTGGTTCGCCGGTCAAAGCCTCTTCG 300…709 CACAGCCTAATGTGACCGCTT-CGCCGGCGAC 739…1016 GTTTAGTGAGCGTTCCCTGA---CCAAGGGTCT 1045
C2_19 Allele 1 268 AACCCCCTTGA-TCGCCGGTCAAAGCCTCTTCG 299…708 CACAGCCTAATGTGACCGCTT-CGCCGGCGAC 738…1015 GTTTAGTGAGCGTTCCCTGATA-CCAAGGGTCT 1046
      Allele 2 268 AACCCCCTTGA-TCGCCGGTCAAAGCCTCTTCG 299…708 CACAGCCTAATGTGACCGCTT-CGCCGGCGAC 738…1015 GTTTAGTGAGCGTTCCCTGA---CCAAGGGTCT 1044
C2_20 Allele 1 268 AACCCCCTTGA-TCGCCGGTCAAAGCCTCTTCG 299…708 CACAGCCTAATGTGACCGCTTTCGCCGGCGAC 739…1016 GTTTAGTGAGCGTTCCCTGATA-CCAAGGGTCT 1047
      Allele 2 268 AACCCCCTTGA-TCGCCGGTCAAAGCCTCTTCG 299…708 CACAGCCTAATGTGACCGCTTTCGCCGGCGAC 739…1016 GTTTAGTGAGCGTTCCCTGA---CCAAGGGTCT 1045
C2_21 Allele 1 268 AACCCCCTTG--TCGCCGGTCAAAGCCTCTTCG 298…707 CACAGCCTAATGTGACCGCTT-CGCCGGCGAC 737…1014 GTTTAGTGAGCGTTCCCTGATA-CCAAGGGTCT 1045
      Allele 2 268 AACCCCCTTG--TCGCCGGTCAAAGCCTCTTCG 298…707 CACAGCCTAATGTGACCGCTT-CGCCGGCGAC 737…1014 GTTTAGTGAGCGTTCCCTGATA-CCAAGGGTCT 1045
C2_22 Allele 1 268 AACCCCCTTGA-TCGCCGGTCAAAGCCTCTTCG 299…708 CACAGCCTAATGTGACCGCTT-CGCCGGCGAC 738…1015 GTTTAGTGAGCGTTCCCTGATA-CCAAGGGTCT 1046
      Allele 2 268 AACCCCCTTGA-TCGCCGGTCAAAGCCTCTTCG 299…708 CACAGCCTAATGTGACCGCTT-CGCCGGCGAC 738…1015 GTTTAGTGAGCGTTCCCTGA---CCAAGGGTCT 1044
C2_23 Allele 1 268 AACCCCCTTGA-TCGCCGGTCAAAGCCTCTTCG 299…708 CACAGCCTAATGTGACCGCTT-CGCCGGCGAC 738…1015 GTTTAGTGAGCGTTCCCTGATAACCAAGGGTCT 1047
      Allele 2 268 AACCCCCTTGA-TCGCCGGTCAAAGCCTCTTCG 299…708 CACAGCCTAATGTGACCGCTT-CGCCGGCGAC 738…1015 GTTTAGTGAGCGTTCCCTGA---CCAAGGGTCT 1044
C2_24 Allele 1 268 AACCCCCTTGA-TCGCCGGTCAAAGCCTCTTCG 299…708 CACAGCCTAATGTGACCGCTT-CGCCGGCGAC 738…1015 GTTTAGTGAGCGTTCCCTGATA-CCAAGGGTCT 1046
      Allele 2 268 AACCCCCTTGA-TCGCCGGTCAAAGCCTCTTCG 299…708 CACAGCCTAATGTGACCGCTT-CGCCGGCGAC 738…1015 GTTTAGTGAGCGTTCCCTGA---CCAAGGGTCT 1044
C2_25 Allele 1 268 AACCCCCTTGA-TCGCCGGTCAAAGCCTCTTCG 299…708 CACAGCCTAATGTGACCGCTT-CGCCGGCGAC 738…1015 GTTTAGTGAGCGTTCCCTGATA-CCAAGGGTCT 1046
      Allele 2 268 AACCCCCTTGA-TCGCCGGTCAAAGCCTCTTCG 299…708 CACAGCCTAATGTGACCGCTT-CGCCGGCGAC 738…1015 GTTTAGTGAGCGTTCCCTGA---CCAAGGGTCT 1044
C2_26 Allele 1 268 AACCCCCTTGA-TCGCCGGTCAAAGCCTCTTCG 299…708 CACAGCCTAATGTGACCGCTT-CGCCGGCGAC 738…1015 GTTTAGTGAGCGTTCCCTGATA-CCAAGGGTCT 1046
      Allele 2 268 AACCCCCTTGA-TCGCCGGTCAAAGCCTCTTCG 299…708 CACAGCCTAATGTGACCGCTT-CGCCGGCGAC 738…1015 GTTTAGTGAGCGTTCCCTGA---CCAAGGGTCT 1044
C2_27 Allele 1 268 AACCCCCTTGA-TCGCCGGTCAAAGCCTCTTCG 299…708 CACAGCCTAATGTGACCGCTTTCGCCGGCGAC 739…1016 GTTTAGTGAGCGTTCCCTGATA-CCAAGGGTCT 1047
      Allele 2 268 AACCCCCTTGA-TCGCCGGTCAAAGCCTCTTCG 299…708 CACAGCCTAATGTGACCGCTTTCGCCGGCGAC 739…1016 GTTTAGTGAGCGTTCCCTGA---CCAAGGGTCT 1045
C2_28 Allele 1 268 AACCCCCTTGA-TCGCCGGTCAAAGCCTCTTCG 299…708 CACAGCCTAATGTGACCGCTT-CGCCGGCGAC 738…1015 GTTTAGTGAGCGTTCCCTGATAACCAAGGGTCT 1047
      Allele 2 268 AACCCCCTTGA-TCGCCGGTCAAAGCCTCTTCG 299…708 CACAGCCTAATGTGACCGCTT-CGCCGGCGAC 738…1015 GTTTAGTGAGCGTTCCCTGA---CCAAGGGTCT 1044
C2_29 Allele 1 268 AACCCCCTTGA-TCGCCGGTCAAAGCCTCTTCG 299…708 CACAGCCTAATGTGACCGCTT-CGCCGGCGAC 738…1015 GTTTAGTGAGCGTTCCCTGATA-CCAAGGGTCT 1046
      Allele 2 268 AACCCCCTTGA-TCGCCGGTCAAAGCCTCTTCG 299…708 CACAGCCTAATGTGACCGCTT-CGCCGGCGAC 738…1015 GTTTAGTGAGCGTTCCCTGA---CCAAGGGTCT 1044
C2_30 Allele 1 268 AACCCCCTTGA-TCGCCGGTCAAAGCCTCTTCG 299…708 CACAGCCTAATGTGACCGCTT-CGCCGGCGAC 738…1015 GTTTAGTGAGCGTTCCCTGATA-CCAAGGGTCT 1046
      Allele 2 268 AACCCCCTTGA-TCGCCGGTCAAAGCCTCTTCG 299…708 CACAGCCTAATGTGACCGCTT-CGCCGGCGAC 738…1015 GTTTAGTGAGCGTTCCCTGA---CCAAGGGTCT 1044
C2_31 Allele 1 268 AACCCCCTTGA-TCGCCGGTCAAAGCCTCTTCG 299…708 CACAGCCTAATGTGACCGCTT-CGCCGGCGAC 738…1015 GTTTAGTGAGCGTTCCCTGATA-CCAAGGGTCT 1046
      Allele 2 268 AACCCCCTTGA-TCGCCGGTCAAAGCCTCTTCG 299…708 CACAGCCTAATGTGACCGCTT-CGCCGGCGAC 738…1015 GTTTAGTGAGCGTTCCCTG-----CAAGGGTCT 1042
C2_32 Allele 1 268 AACCCCCTTGA-TCGCCGGTCAAAGCCTCTTCG 299…708 CACAGCCTAATGTGACCGCTT-CGCCGGCGAC 738…1015 GTTTAGTGAGCGTTCCCTGATA-CCAAGGGTCT 1046
      Allele 2 268 AACCCCCTTGA-TCGCCGGTCAAAGCCTCTTCG 299…708 CACAGCCTAATGTGACCGCTT-CGCCGGCGAC 738…1015 GTTTAGTGAGCGTTCCCTG-----CAAGGGTCT 1042
C2_33 Allele 1 268 AACCCCCTTGA-TCGCCGGTCAAAGCCTCTTCG 299…708 CACAGCCTAATGTGACCGCTT-CGCCGGCGAC 738…1015 GTTTAGTGAGCGTTCCCTGATTACCAAGGGTCT 1047
      Allele 2 268 AACCCCCTTGA-TCGCCGGTCAAAGCCTCTTCG 299…708 CACAGCCTAATGTGACCGCTT-CGCCGGCGAC 738…1015 GTTTAGTGAGCGTTCCCTGA---CCAAGGGTCT 1044
C2_34 Allele 1 268 AACCCCCTTGA-TCGCCGGTCAAAGCCTCTTCG 299…708 CACAGCCTAATGTGACCGCTT-CGCCGGCGAC 738…1015 GTTTAGTGAGCGTTCCCTGA---CCAAGGGTCT 1044
      Allele 2 268 AACCCCCTTGA-TCGCCGGTCAAAGCCTCTTCG 299…708 CACAGCCTAATGTGACCGCTT-CGCCGGCGAC 738…1015 GTTTAGTGAGCGTTCC------ACCAAGGGTCT 1040
C2_35 Allele 1 268 AACCCCCTTGA-TCGCCGGTCAAAGCCTCTTCG 299…708 CACAGCCTAATGTGACCGCTT-CGCCGGCGAC 738…1015 GTTTAGTGAGCGTTCCCTGATA-CCAAGGGTCT 1046
      Allele 2 268 AACCCCCTTGA-TCGCCGGTCAAAGCCTCTTCG 299…708 CACAGCCTAATGTGACCGCTT-CGCCGGCGAC 738…1015 GTTTAGTGAGCGTTCCCTGA---CCAAGGGTCT 1044
C2_36 Allele 1 268 AACCCCCTTGA-TCGCCGGTCAAAGCCTCTTCG 299…708 CACAGCCTAATGTGACCGCTT-CGCCGGCGAC 738…1015 GTTTAGTGAGCGTTCCCTGATA-CCAAGGGTCT 1046
      Allele 2 268 AACCCCCTTGA-TCGCCGGTCAAAGCCTCTTCG 299…708 CACAGCCTAATGTGACCGCTT-CGCCGGCGAC 738…1015 GTTTAGTGAGCGTTCCCTGA---CCAAGGGTCT 1044
C2_37 Allele 1 268 AACCCCCTTG--TCGCCGGTCAAAGCCTCTTCG 298…707 CACAGCCTAATGTGACCGCTT-CGCCGGCGAC 737…1014 GTTTAGTGAGCGTTCCCTGATA-CCAAGGGTCT 1045
      Allele 2 268 AACCCCCTTG--TCGCCGGTCAAAGCCTCTTCG 298…707 CACAGCCTAATGTGACCGCTT-CGCCGGCGAC 737…1014 GTTTAGTGAGCGTTCCCTGA---CCAAGGGTCT 1043
C2_38 Allele 1 268 AACCCCCTTG--TCGCCGGTCAAAGCCTCTTCG 298…707 CACAGCCTAATGTGACCGCTT-CGCCGGCGAC 737…1014 GTTTAGTGAGCGTTCCCTGATA-CCAAGGGTCT 1045
      Allele 2 268 AACCCCCTTG--TCGCCGGTCAAAGCCTCTTCG 298…707 CACAGCCTAATGTGACCGCTT-CGCCGGCGAC 737…1014 GTTTAGTGAGCGTTCCCTGA---CCAAGGGTCT 1043
C2_39 Allele 1 268 AACCCCCTTG--TCGCCGGTCAAAGCCTCTTCG 298…707 CACAGCCTAATGTGACCGCTT-CGCCGGCGAC 737…1014 GTTTAGTGAGCGTTCCCTGATA-CCAAGGGTCT 1045
      Allele 2 268 AACCCCCTTG--TCGCCGGTCAAAGCCTCTTCG 298…707 CACAGCCTAATGTGACCGCTT-CGCCGGCGAC 737…1014 GTTTAGTGAGCGTTCCCTGA---CCAAGGGTCT 1043
C2_40 Allele 1 268 AACCCCCTTG--TCGCCGGTCAAAGCCTCTTCG 298…707 CACAGCCTAATGTGACCGCTT-CGCCGGCGAC 737…1014 GTTTAGTGAGCGTTCCCTGATA-CCAAGGGTCT 1045
      Allele 2 268 AACCCCCTTG--TCGCCGGTCAAAGCCTCTTCG 298…707 CACAGCCTAATGTGACCGCTT-CGCCGGCGAC 737…1014 GTTTAGTGAGCGTTCCCTGA---CCAAGGGTCT 1043
C2_41 Allele 1 268 AACCCCCTTGA-TCGCCGGTCAAAGCCTCTTCG 299…708 CACAGCCTAATGTGACCGCTT-CGCCGGCGAC 738…1015 GTTTAGTGAGCGTTCCCTGATA-CCAAGGGTCT 1046
      Allele 2 268 AACCCCCTTGA-TCGCCGGTCAAAGCCTCTTCG 299…708 CACAGCCTAATGTGACCGCTT-CGCCGGCGAC 738…1015 GTTTAGTGAGCGTTCCCTGA---CCAAGGGTCT 1044
C2_42 Allele 1 268 AACCCCCTTGA-TCGCCGGTCAAAGCCTCTTCG 299…708 CACAGCCTAATGTGACCGCTT-CGCCGGCGAC 738…1015 GTTTAGTGAGCGTTCCCTGATA-CCAAGGGTCT 1046
      Allele 2 268 AACCCCCTTGA-TCGCCGGTCAAAGCCTCTTCG 299…708 CACAGCCTAATGTGACCGCTT-CGCCGGCGAC 738…1015 GTTTAGTGAGCGTTCCCTGA---CCAAGGGTCT 1044
```

```
WT    Allele 1 268 AACCCCCTTG--TCGCCGGTCAAAGCCTCTTCG 298…707 CACAGCCTAATGTGACCGCTT-CGCCGGCGAC 737…1014 GTTTAGTGAGCGTTCCCTGAT--CCAAGGGTCT 1044
      Allele 2 268 AACCCCCTTG--TCGCCGGTCAAAGCCTCTTCG 298…707 CACAGCCTAATGTGACCGCTT-CGCCGGCGAC 737…1014 GTTTAGTGAGCGTTCCCTGAT--CCAAGGGTCT 1044
C2_43 Allele 1 268 AACCCCCTTG--TCGCCGGTCAAAGCCTCTTCG 298…707 CACAGCCTAATGTGACCGCTT-CGCCGGCGAC 737…1014 GTTTAGTGAGCGTTCCCTGATA-CCAAGGGTCT 1045
      Allele 2 268 AACCCCCTTG--TCGCCGGTCAAAGCCTCTTCG 298…707 CACAGCCTAATGTGACCGCTT-CGCCGGCGAC 737…1014 GTTTAGTGAGCGTTCCCTGA---CCAAGGGTCT 1043
C2_44 Allele 1 268 AACCCCCTTG--TCGCCGGTCAAAGCCTCTTCG 298…707 CACAGCCTAATGTGACCGCTT-CGCCGGCGAC 737…1014 GTTTAGTGAGCGTTCCCTGATA-CCAAGGGTCT 1045
      Allele 2 268 AACCCCCTTG--TCGCCGGTCAAAGCCTCTTCG 298…707 CACAGCCTAATGTGACCGCTT-CGCCGGCGAC 737…1014 GTTTAGTGAGCGTTCCCTGA---CCAAGGGTCT 1043
C2_45 Allele 1 268 AACCCCCTTG--TCGCCGGTCAAAGCCTCTTCG 298…707 CACAGCCTAATGTGACCGCTT-CGCCGGCGAC 737…1014 GTTTAGTGAGCGTTCCCTGATA-CCAAGGGTCT 1045
      Allele 2 268 AACCCCCTTG--TCGCCGGTCAAAGCCTCTTCG 298…707 CACAGCCTAATGTGACCGCTT-CGCCGGCGAC 737…1014 GTTTAGTGAGCGTTCCCTGA---CCAAGGGTCT 1043
C2_46 Allele 1 268 AACCCCCTTG--TCGCCGGTCAAAGCCTCTTCG 298…707 CACAGCCTAATGTGACCGCTT-CGCCGGCGAC 737…1014 GTTTAGTGAGCGTTCCCTGATA-CCAAGGGTCT 1045
      Allele 2 268 AACCCCCTTG--TCGCCGGTCAAAGCCTCTTCG 298…707 CACAGCCTAATGTGACCGCTT-CGCCGGCGAC 737…1014 GTTTAGTGAGCGTTCCCTGA---CCAAGGGTCT 1043
C2_47 Allele 1 268 AACCCCCTTG--TCGCCGGTCAAAGCCTCTTCG 298…707 CACAGCCTAATGTGACCGCTT-CGCCGGCGAC 737…1014 GTTTAGTGAGCGTTC------AACCAAGGGTCT 1040
      Allele 2 268 AACCCCCTTG--TCGCCGGTCAAAGCCTCTTCG 298…707 CACAGCCTAATGTGACCGCTT-CGCCGGCGAC 737…1014 GTTTAGTGAGCGTTCCCTGA---CCAAGGGTCT 1043
C2_48 Allele 1 268 AACCCCCTTG--TCGCCGGTCAAAGCCTCTTCG 298…707 CACAGCCTAATGTGACCGCTT-CGCCGGCGAC 737…1014 GTTTAGTGAGCGTTCCCTGATA-CCAAGGGTCT 1045
      Allele 2 268 AACCCCCTTG--TCGCCGGTCAAAGCCTCTTCG 298…707 CACAGCCTAATGTGACCGCTT-CGCCGGCGAC 737…1014 GTTTAGTGAGCGTTCCCTGA---CCAAGGGTCT 1043
C2_49 Allele 1 268 AACCCCCTTGG-TCGCCGGTCAAAGCCTCTTCG 299…708 CACAGCCTAATGTGACCGCTT-CGCCGGCGAC 738…1015 GTTTAGTGAGCGTTCCCTGATA-CCAAGGGTCT 1046
      Allele 2 268 AACCCCCTTGG-TCGCCGGTCAAAGCCTCTTCG 299…708 CACAGCCTAATGTGACCGCTT-CGCCGGCGAC 738…1015 GTTTAGTGAGCGTTCCCTGA---CCAAGGGTCT 1044
C2_50 Allele 1 268 AACCCCCTTG--TCGCCGGTCAAAGCCTCTTCG 298…707 CACAGCCTAATGTGACCGCTT-CGCCGGCGAC 737…1014 GTTTAGTGAGCGTTCCCTGATA-CCAAGGGTCT 1045
      Allele 2 268 AACCCCCTTG--TCGCCGGTCAAAGCCTCTTCG 298…707 CACAGCCTAATGTGACCGCTT-CGCCGGCGAC 737…1014 GTTTAGTGAGCGTTCCCTGA---CCAAGGGTCT 1043
C2_51 Allele 1 268 AACCCCCTTG--TCGCCGGTCAAAGCCTCTTCG 298…707 CACAGCCTAATGTGACCGCTT-CGCCGGCGAC 737…1014 GTTTAGTGAGCGTTCCCTGATA-CCAAGGGTCT 1045
      Allele 2 268 AACCCCCTTG--TCGCCGGTCAAAGCCTCTTCG 298…707 CACAGCCTAATGTGACCGCTT-CGCCGGCGAC 737…1014 GTTTAGTGAGCGTTCCCTGA---CCAAGGGTCT 1043
C2_52 Allele 1 268 AACCCCCTTG--TCGCCGGTCAAAGCCTCTTCG 298…707 CACAGCCTAATGTGACCGCTT-CGCCGGCGAC 737…1014 GTTTAGTGAGCGTTCCCTGATA-CCAAGGGTCT 1045
      Allele 2 268 AACCCCCTTG--TCGCCGGTCAAAGCCTCTTCG 298…707 CACAGCCTAATGTGACCGCTT-CGCCGGCGAC 737…1014 GTTTAGTGAGCGTTCCCTGA---CCAAGGGTCT 1043
C2_53 Allele 1 268 AACCCCCTTG--TCGCCGGTCAAAGCCTCTTCG 298…707 CACAGCCTAATGTGACCGCTT-CGCCGGCGAC 737…1014 GTTTAGTGAGCGTTCCCTGATA-CCAAGGGTCT 1045
      Allele 2 268 AACCCCCTTG--TCGCCGGTCAAAGCCTCTTCG 298…707 CACAGCCTAATGTGACCGCTT-CGCCGGCGAC 737…1014 GTTTAGTGAGCGTTCCCTGA---CCAAGGGTCT 1043
C2_54 Allele 1 268 AACCCCCTTGGTTCGCCGGTCAAAGCCTCTTCG 300…709 CACAGCCTAATGTGACCGCTT-CGCCGGCGAC 739…1016 GTTTAGTGAGCGTTCCCTGATA-CCAAGGGTCT 1047
      Allele 2 268 AACCCCCTTGGTTCGCCGGTCAAAGCCTCTTCG 300…709 CACAGCCTAATGTGACCGCTT-CGCCGGCGAC 739…1016 GTTTAGTGAGCGTTCCCTGA---CCAAGGGTCT 1044
C2_55 Allele 1 268 AACCCCCTTG--TCGCCGGTCAAAGCCTCTTCG 298…707 CACAGCCTAATGTGACCGCTT-CGCCGGCGAC 737…1014 GTTTAGTGAGCGTTCCCTGATA-CCAAGGGTCT 1045
      Allele 2 268 AACCCCCTTG--TCGCCGGTCAAAGCCTCTTCG 298…707 CACAGCCTAATGTGACCGCTT-CGCCGGCGAC 737…1014 GTTTAGTGAGCGTTCCCTGA---CCAAGGGTCT 1043
C2_56 Allele 1 268 AACCCCCTTG-TTCGCCGGTCAAAGCCTCTTCG 299…708 CACAGCCTAATGTGACCGCTT-CGCCGGCGAC 738…1015 GTTTAGTGAGCGTTCCCTGATA-CCAAGGGTCT 1046
      Allele 2 268 AACCCCCTTG-TTCGCCGGTCAAAGCCTCTTCG 299…708 CACAGCCTAATGTGACCGCTT-CGCCGGCGAC 738…1015 GTTTAGTGAGCGTTCCCTGA---CCAAGGGTCT 1044
C2_57 Allele 1 268 AACCCCCTT---TCGCCGGTCAAAGCCTCTTCG 297…706 CACAGCCTAATGTGACCGCTT-CGCCGGCGAC 736…1013 GTTTAGTGAGCGTTCCCTGATA-CCAAGGGTCT 1044
      Allele 2 268 AACCCCCTTG--TCGCCGGTCAAAGCCTCTTCG 298…707 CACAGCCTAATGTGACCGCTT-CGCCGGCGAC 737…1014 GTTTAGTGAGCGTTCCCTGA---CCAAGGGTCT 1043
C2_58 Allele 1 268 AACCCCCTTGA-TCGCCGGTCAAAGCCTCTTCG 299…708 CACAGCCTAATGTGACCGCTT-CGCCGGCGAC 738…1015 GTTTAGTGAGCGTTCCCTGATA-CCAAGGGTCT 1046
      Allele 2 268 AACCCCCTTGA-TCGCCGGTCAAAGCCTCTTCG 299…708 CACAGCCTAATGTGACCGCTT-CGCCGGCGAC 738…1015 GTTTAGTGAGCGTTCCCTGA---CCAAGGGTCT 1044
C2_59 Allele 1 268 AACCCCCTTG--TCGCCGGTCAAAGCCTCTTCG 298…707 CACAGCCTAATGTGACCGCTT-CGCCGGCGAC 737…1014 GTTTAGTGAGCGTTCCCTGATA-CCAAGGGTCT 1045
      Allele 2 268 AACCCCCTTG--TCGCCGGTCAAAGCCTCTTCG 298…707 CACAGCCTAATGTGACCGCTT-CGCCGGCGAC 737…1014 GTTTAGTGAGCGTTCCCTGA---CCAAGGGTCT 1043
C2_60 Allele 1 268 AACCCCCTTG--TCGCCGGTCAAAGCCTCTTCG 298…707 CACAGCCTAATGTGACCGCTT-CGCCGGCGAC 737…1014 GTTTAGTGAGCGTTCCCTGATA-CCAAGGGTCT 1045
      Allele 2 268 AACCCCCTTG--TCGCCGGTCAAAGCCTCTTCG 298…707 CACAGCCTAATGTGACCGCTT-CGCCGGCGAC 737…1014 GTTTAGTGAGCGTTCCCTGA---CCAAGGGTCT 1043
C2_61 Allele 1 268 AACCCCCTTG--TCGCCGGTCAAAGCCTCTTCG 298…707 CACAGCCTAATGTGACCGCTT-CGCCGGCGAC 737…1014 GTTTAGTGAGCGTTCCCTGATA-CCAAGGGTCT 1045
      Allele 2 268 AACCCCCTTG--TCGCCGGTCAAAGCCTCTTCG 298…707 CACAGCCTAATGTGACCGCTT-CGCCGGCGAC 737…1014 GTTTAGTGAGCGTTCCCTGA---CCAAGGGTCT 1043
C2_62 Allele 1 268 AACCCCCTTG--TCGCCGGTCAAAGCCTCTTCG 298…707 CACAGCCTAATGTGACCGCTT-CGCCGGCGAC 737…1014 GTTTAGTGAGCGTTCCCTGATA-CCAAGGGTCT 1045
      Allele 2 268 AACCCCCTTG--TCGCCGGTCAAAGCCTCTTCG 298…707 CACAGCCTAATGTGACCGCTT-CGCCGGCGAC 737…1014 GTTTAGTGAGCGTTCCCTGA---CCAAGGGTCT 1043
C2_63 Allele 1 268 AACCCCCTTG--TCGCCGGTCAAAGCCTCTTCG 298…707 CACAGCCTAATGTGACCGCTT-CGCCGGCGAC 737…1014 GTTTAGTGAGCGTTCCCTGATA-CCAAGGGTCT 1045
      Allele 2 268 AACCCCCTTG--TCGCCGGTCAAAGCCTCTTCG 298…707 CACAGCCTAATGTGACCGCTT-CGCCGGCGAC 737…1014 GTTTAGTGAGCGTTCCCTGA---CCAAGGGTCT 1043
C2_64 Allele 1 268 AACCCCCTTGA-TCGCCGGTCAAAGCCTCTTCG 299…708 CACAGCCTAATGTGACCGCTT-CGCCGGCGAC 738…1015 GTTTAGTGAGCGTTCCCTGATA-CCAAGGGTCT 1046
      Allele 2 268 AACCCCCTTGA-TCGCCGGTCAAAGCCTCTTCG 299…708 CACAGCCTAATGTGACCGCTT-CGCCGGCGAC 738…1015 GTTTAGTGAGCGTTCCCTGA---CCAAGGGTCT 1044
C2_65 Allele 1 268 AACCCCCTTGA-TCGCCGGTCAAAGCCTCTTCG 299…708 CACAGCCTAATGTGACCGCTT-CGCCGGCGAC 738…1015 GTTTAGTGAGCGTTCCCTGATA-CCAAGGGTCT 1046
      Allele 2 268 AACCCCCTTG--TCGCCGGTCAAAGCCTCTTCG 298…707 CACAGCCTAATGTGACCGCTT-CGCCGGCGAC 737…1014 GTTTAGTGAGCGTTCCCTGA---CCAAGGGTCT 1043
C2_66 Allele 1 268 AACCCCCTTG--TCGCCGGTCAAAGCCTCTTCG 298…707 CACAGCCTAATGTGACCGCTT-CGCCGGCGAC 737…1014 GTTTAGTGAGCGTTCCCTGATA-CCAAGGGTCT 1045
      Allele 2 268 AACCCCCTTG--TCGCCGGTCAAAGCCTCTTCG 298…707 CACAGCCTAATGTGACCGCTT-CGCCGGCGAC 737…1014 GTTTAGTGAGCGTTCCCTGA---CCAAGGGTCT 1043
C2_67 Allele 1 268 AACCCCCTTG--TCGCCGGTCAAAGCCTCTTCG 298…707 CACAGCCTAATGTGACCGCTT-CGCCGGCGAC 737…1014 GTTTAGTGAGCGTTCCCTGATA-CCAAGGGTCT 1045
      Allele 2 268 AACCCCCTTG--TCGCCGGTCAAAGCCTCTTCG 298…707 CACAGCCTAATGTGACCGCTT-CGCCGGCGAC 737…1014 GTTTAGTGAGCGTTCCCTGA---CCAAGGGTCT 1043
C2_68 Allele 1 268 AACCCCCTTG--TCGCCGGTCAAAGCCTCTTCG 298…707 CACAGCCTAATGTGACCGCTT-CGCCGGCGAC 737…1014 GTTTAGTGAGCGTTC------AACCAAGGGTCT 1040
      Allele 2 268 AACCCCCTTG--TCGCCGGTCAAAGCCTCTTCG 298…707 CACAGCCTAATGTGACCGCTT-CGCCGGCGAC 737…1014 GTTTAGTGAGCGTTCCCTGA---CCAAGGGTCT 1043
C2_69 Allele 1 268 AACCCCCTTG--TCGCCGGTCAAAGCCTCTTCG 298…707 CACAGCCTAATGTGACCGCTT-CGCCGGCGAC 737…1014 GTTTAGTGAGCGTTCCCTGATA-CCAAGGGTCT 1045
      Allele 2 268 AACCCCCTTG--TCGCCGGTCAAAGCCTCTTCG 298…707 CACAGCCTAATGTGACCGCTT-CGCCGGCGAC 737…1014 GTTTAGTGAGCGTTCCCTGA---CCAAGGGTCT 1043
C2_70 Allele 1 268 AACCCCCTTG--TCGCCGGTCAAAGCCTCTTCG 298…707 CACAGCCTAATGTGACCGCTT-CGCCGGCGAC 737…1014 GTTTAGTGAGCGTTCCCTGATTACCAAGGGTCT 1046
      Allele 2 268 AACCCCCTTG--TCGCCGGTCAAAGCCTCTTCG 298…707 CACAGCCTAATGTGACCGCTT-CGCCGGCGAC 737…1014 GTTTAGTGAGCGTTCCCTGA---CCAAGGGTCT 1043
C2_71 Allele 1 268 AACCCCCTTG--TCGCCGGTCAAAGCCTCTTCG 298…707 CACAGCCTAATGTGACCGCTT-CGCCGGCGAC 737…1014 GTTTAGTGAGCGTTCCCTGATA-CCAAGGGTCT 1045
      Allele 2 268 AACCCCCTTG--TCGCCGGTCAAAGCCTCTTCG 298…707 CACAGCCTAATGTGACCGCTT-CGCCGGCGAC 737…1014 GTTTAGTGAGCGTTCCCTGA---CCAAGGGTCT 1043
C2_72 Allele 1 268 AACCCCCTTGA-TCGCCGGTCAAAGCCTCTTCG 299…708 CACAGCCTAATGTGACCGCTT-CGCCGGCGAC 738…1015 GTTTAGTGAGCGTTCCCTGATTACCAAGGGTCT 1047
      Allele 2 268 AACCCCCTTGA-TCGCCGGTCAAAGCCTCTTCG 299…708 CACAGCCTAATGTGACCGCTT-CGCCGGCGAC 738…1015 GTTTAGTGAGCGTTCCCTGA---CCAAGGGTCT 1044
C2_73 Allele 1 268 AACCCCCTTGC-TCGCCGGTCAAAGCCTCTTCG 299…708 CACAGCCTAATGTGACCGCTT-CGCCGGCGAC 738…1015 GTTTAGTGAGCGTTCCCTGATA-CCAAGGGTCT 1046
      Allele 2 268 AACCCCCTTGC-TCGCCGGTCAAAGCCTCTTCG 299…708 CACAGCCTAATGTGACCGCTT-CGCCGGCGAC 738…1015 GTTTAGTGAGCGTTCCCTGA---CCAAGGGTCT 1044
C2_74 Allele 1 268 AACCCCCTTGA-TCGCCGGTCAAAGCCTCTTCG 299…708 CACAGCCTAATGTGACCGCTTTCGCCGGCGAC 739…1016 GTTTAGTGAGCGTTCCCTGATA-CCAAGGGTCT 1047
      Allele 2 268 AACCCCCTTGA-TCGCCGGTCAAAGCCTCTTCG 299…708 CACAGCCTAATGTGACCGCTTTCGCCGGCGAC 739…1016 GTTTAGTGAGCGTTCCCTGA---CCAAGGGTCT 1045
C2_75 Allele 1 268 AACCCCCTTGA-TCGCCGGTCAAAGCCTCTTCG 299…708 CACAGCCTAATGTGACCGCTT-CGCCGGCGAC 738…1015 GTTTAGTGAGCGTTCCCTGATA-CCAAGGGTCT 1046
      Allele 2 268 AACCCCCTTGA-TCGCCGGTCAAAGCCTCTTCG 299…708 CACAGCCTAATGTGACCGCTT-CGCCGGCGAC 738…1015 GTTTAGTGAGCGTTCCCTGA---CCAAGGGTCT 1044
C2_76 Allele 1 268 AACCCCCTTGA-TCGCCGGTCAAAGCCTCTTCG 299…708 CACAGCCTAATGTGACCGCTT-CGCCGGCGAC 738…1015 GTTTAGTGAGCGTTCCCTGATTACCAAGGGTCT 1047
      Allele 2 268 AACCCCCTTGA-TCGCCGGTCAAAGCCTCTTCG 299…708 CACAGCCTAATGTGACCGCTT-CGCCGGCGAC 738…1015 GTTTAGTGAGCGTTCCCTGA---CCAAGGGTCT 1044
C2_77 Allele 1 268 AACCCCCTTGA-TCGCCGGTCAAAGCCTCTTCG 299…708 CACAGCCTAATGTGACCGCTTTCGCCGGCGAC 739…1016 GTTTAGTGAGCGTTCCCTGATA-CCAAGGGTCT 1047
      Allele 2 268 AACCCCCTTGA-TCGCCGGTCAAAGCCTCTTCG 299…708 CACAGCCTAATGTGACCGCTTTCGCCGGCGAC 739…1016 GTTTAGTGAGCGTTCCCTGA---CCAAGGGTCT 1045
C2_78 Allele 1 268 AACCCCCTTGA-TCGCCGGTCAAAGCCTCTTCG 299…708 CACAGCCTAATGTGACCGCTT-CGCCGGCGAC 738…1015 GTTTAGTGAGCGTTCCCTGATA-CCAAGGGTCT 1046
      Allele 2 268 AACCCCCTTGA-TCGCCGGTCAAAGCCTCTTCG 299…708 CACAGCCTAATGTGACCGCTT-CGCCGGCGAC 738…1015 GTTTAGTGAGCGTTCCCTGA---CCAAGGGTCT 1044
C2_79 Allele 1 268 AACCCCCTTG--TCGCCGGTCAAAGCCTCTTCG 298…707 CACAGCCTAATGTGACCGCTT-CGCCGGCGAC 737…1014 GTTTAGTGAGCGTTC------AACCAAGGGTCT 1040
      Allele 2 268 AACCCCCTTG--TCGCCGGTCAAAGCCTCTTCG 298…707 CACAGCCTAATGTGACCGCTT-CGCCGGCGAC 737…1014 GTTTAGTGAGCGTTCCCTGA---CCAAGGGTCT 1043
C2_80 Allele 1 268 AACCCCCTTGA-TCGCCGGTCAAAGCCTCTTCG 299…708 CACAGCCTAATGTGACCGCTT-CGCCGGCGAC 738…1015 GTTTAGTGAGCGTTCCCTGATAACCAAGGGTCT 1047
      Allele 2 268 AACCCCCTTGA-TCGCCGGTCAAAGCCTCTTCG 299…708 CACAGCCTAATGTGACCGCTT-CGCCGGCGAC 738…1015 GTTTAGTGAGCGTTCCCTGA---CCAAGGGTCT 1044
C2_81 Allele 1 268 AACCCCCTTG--TCGCCGGTCAAAGCCTCTTCG 298…707 CACAGCCTAATGTGACCGCTT-CGCCGGCGAC 737…1014 GTTTAGTGAGCGTTC------AACCAAGGGTCT 1040
      Allele 2 268 AACCCCCTTG--TCGCCGGTCAAAGCCTCTTCG 298…707 CACAGCCTAATGTGACCGCTT-CGCCGGCGAC 737…1014 GTTTAGTGAGCGTTCCCTGA---CCAAGGGTCT 1043
C2_82 Allele 1 268 AACCCCCTTG--TCGCCGGTCAAAGCCTCTTCG 298…707 CACAGCCTAATGTGACCGCTT-CGCCGGCGAC 737…1014 GTTTAGTGAGCGTTC------AACCAAGGGTCT 1040
      Allele 2 268 AACCCCCTTG--TCGCCGGTCAAAGCCTCTTCG 298…707 CACAGCCTAATGTGACCGCTT-CGCCGGCGAC 737…1014 GTTTAGTGAGCGTTCCCTGA---CCAAGGGTCT 1043
C2_83 Allele 1 268 AACCCCCTTGGTTCGCCGGTCAAAGCCTCTTCG 300…709 CACAGCCTAATGTGACCGCTT-CGCCGGCGAC 739…1016 GTTTAGTGAGCGTTCCCTGATA-CCAAGGGTCT 1047
      Allele 2 268 AACCCCCTTGGTTCGCCGGTCAAAGCCTCTTCG 300…709 CACAGCCTAATGTGACCGCTT-CGCCGGCGAC 739…1016 GTTTAGTGAGCGTTCCCTGA---CCAAGGGTCT 1045
C2_84 Allele 1 268 AACCCCCTTGGTTCGCCGGTCAAAGCCTCTTCG 300…709 CACAGCCTAATGTGACCGCTT-CGCCGGCGAC 739…1016 GTTTAGTGAGCGTTCCCTGATA-CCAAGGGTCT 1047
      Allele 2 268 AACCCCCTTGGTTCGCCGGTCAAAGCCTCTTCG 300…709 CACAGCCTAATGTGACCGCTT-CGCCGGCGAC 739…1016 GTTTAGTGAGCGTTCCCTGA---CCAAGGGTCT 1045
```

```
WT    Allele 1 268 AACCCCCTTG--TCGCCGGTCAAAGCCTCTTCG 298…707 CACAGCCTAATGTGACCGCTT-CGCCGGCGAC 737…1014 GTTTAGTGAGCGTTCCCTGAT--CCAAGGGTCT 1044
      Allele 2 268 AACCCCCTTG--TCGCCGGTCAAAGCCTCTTCG 298…707 CACAGCCTAATGTGACCGCTT-CGCCGGCGAC 737…1014 GTTTAGTGAGCGTTCCCTGAT--CCAAGGGTCT 1044
C2_85 Allele 1 268 AACCCCCTTGGTTCGCCGGTCAAAGCCTCTTCG 300…709 CACAGCCTAATGTGACCGCTT-CGCCGGCGAC 739…1016 GTTTAGTGAGCGTTCCCTGATA-CCAAGGGTCT 1047
      Allele 2 268 AACCCCCTTGGTTCGCCGGTCAAAGCCTCTTCG 300…709 CACAGCCTAATGTGACCGCTT-CGCCGGCGAC 739…1016 GTTTAGTGAGCGTTCCCTGA---CCAAGGGTCT 1045
C2_86 Allele 1 268 AACCCCCTTG--TCGCCGGTCAAAGCCTCTTCG 298…707 CACAGCCTAATGTGACCGCTT-CGCCGGCGAC 737…1014 GTTTAGTGAGCGTTCCCTGATA-CCAAGGGTCT 1045
      Allele 2 268 AACCCCCTTG--TCGCCGGTCAAAGCCTCTTCG 298…707 CACAGCCTAATGTGACCGCTT-CGCCGGCGAC 737…1014 GTTTAGTGAGCGTTCCCTGA---CCAAGGGTCT 1043
C2_87 Allele 1 268 AACCCCCTTGA-TCGCCGGTCAAAGCCTCTTCG 299…708 CACAGCCTAATGTGACCGCTT-CGCCGGCGAC 738…1015 GTTTAGTGAGCGTTCCCTGATA-CCAAGGGTCT 1046
      Allele 2 268 AACCCCCTTGA-TCGCCGGTCAAAGCCTCTTCG 299…708 CACAGCCTAATGTGACCGCTT-CGCCGGCGAC 738…1015 GTTTAGTGAGCGTTCCCTGA---CCAAGGGTCT 1044
C2_88 Allele 1 268 AACCCCCTTG--TCGCCGGTCAAAGCCTCTTCG 298…707 CACAGCCTAATGTGACCGCTT-CGCCGGCGAC 737…1014 GTTTAGTGAGCGTTCCCTGATA-CCAAGGGTCT 1045
      Allele 2 268 AACCCCCTTG--TCGCCGGTCAAAGCCTCTTCG 298…707 CACAGCCTAATGTGACCGCTT-CGCCGGCGAC 737…1014 GTTTAGTGAGCGTTCCCTGA---CCAAGGGTCT 1043
C2_89 Allele 1 268 AACCCCCTTG--TCGCCGGTCAAAGCCTCTTCG 298…707 CACAGCCTAATGTGACCGCTT-CGCCGGCGAC 737…1014 GTTTAGTGAGCGTTCCCTGA---CCAAGGGTCT 1043
      Allele 2 268 AACCCCCTTG--TCGCCGGTCAAAGCCTCTTCG 298…707 CACAGCCTAATGTGACCGCTT-CGCCGGCGAC 737…1014 GTTTAGTGAGCGTTCCCTGA---CCAAGGGTCT 1043
C2_90 Allele 1 268 AACCCCCTTG--TCGCCGGTCAAAGCCTCTTCG 298…707 CACAGCCTAATGTGACCGCTT-CGCCGGCGAC 737…1014 GTTTAGTGAGCGTTCCCTGATA-CCAAGGGTCT 1045
      Allele 2 268 AACCCCCTTG--TCGCCGGTCAAAGCCTCTTCG 298…707 CACAGCCTAATGTGACCGCTT-CGCCGGCGAC 737…1014 GTTTAGTGAGCGTTCCCTGA---CCAAGGGTCT 1043
C2_91 Allele 1 268 AACCCCCTTGA-TCGCCGGTCAAAGCCTCTTCG 299…708 CACAGCCTAATGTGACCGCTT-CGCCGGCGAC 738…1015 GTTTAGTGAGCGTTCCCTGATA-CCAAGGGTCT 1046
      Allele 2 268 AACCCCCTTGA-TCGCCGGTCAAAGCCTCTTCG 299…708 CACAGCCTAATGTGACCGCTT-CGCCGGCGAC 738…1015 GTTTAGTGAGCGTTCCCTGA---CCAAGGGTCT 1044
C2_92 Allele 1 268 AACCCCCTTGG-TCGCCGGTCAAAGCCTCTTCG 299…708 CACAGCCTAATGTGACCGCTT-CGCCGGCGAC 738…1015 GTTTAGTGAGCGTTCCCTGATA-CCAAGGGTCT 1046
      Allele 2 268 AACCCCCTTGG-TCGCCGGTCAAAGCCTCTTCG 299…708 CACAGCCTAATGTGACCGCTT-CGCCGGCGAC 738…1015 GTTTAGTGAGCGTTCCCTGA---CCAAGGGTCT 1044
C2_93 Allele 1 268 AACCCCCTTGGTTCGCCGGTCAAAGCCTCTTCG 300…709 CACAGCCTAATGTGACCGCTTTCGCCGGCGAC 740…1017 GTTTAGTGAGCGTTCCCTGATA-CCAAGGGTCT 1048
      Allele 2 268 AACCCCCTTGGTTCGCCGGTCAAAGCCTCTTCG 300…709 CACAGCCTAATGTGACCGCTT-CGCCGGCGAC 739…1016 GTTTAGTGAGCGTTCCCTGA---CCAAGGGTCT 1045
C2_94 Allele 1 268 AACCCCCTTGGTTCGCCGGTCAAAGCCTCTTCG 300…709 CACAGCCTAATGTGACCGCTT-CGCCGGCGAC 739…1016 GTTTAGTGAGCGTTCCCTGATA-CCAAGGGTCT 1047
      Allele 2 268 AACCCCCTTGGTTCGCCGGTCAAAGCCTCTTCG 300…709 CACAGCCTAATGTGACCGCTT-CGCCGGCGAC 739…1016 GTTTAGTGAGCGTTCCCTGA---CCAAGGGTCT 1045
C2_95 Allele 1 268 AACCCCCTTGA-TCGCCGGTCAAAGCCTCTTCG 299…708 CACAGCCTAATGTGACCGCTT-CGCCGGCGAC 738…1015 GTTTAGTGAGCGTTCCCTGATA-CCAAGGGTCT 1046
      Allele 2 268 AACCCCCTTGA-TCGCCGGTCAAAGCCTCTTCG 299…708 CACAGCCTAATGTGACCGCTT-CGCCGGCGAC 738…1015 GTTTAGTGAGCGTTCCCTGA---CCAAGGGTCT 1044
C2_96 Allele 1 268 AACCCCCTTGA-TCGCCGGTCAAAGCCTCTTCG 299…708 CACAGCCTAATGTGACCGCTT-CGCCGGCGAC 738…1015 GTTTAGTGAGCGTTCCCTGATA-CCAAGGGTCT 1046
      Allele 2 268 AACCCCCTTGA-TCGCCGGTCAAAGCCTCTTCG 299…708 CACAGCCTAATGTGACCGCTT-CGCCGGCGAC 738…1015 GTTTAGTGAGCGTTCCCTGA---CCAAGGGTCT 1044
C2_97 Allele 1 268 AACCCCCTTG--TCGCCGGTCAAAGCCTCTTCG 298…707 CACAGCCTAATGTGACCGCTT-CGCCGGCGAC 737…1014 GTTTAGTGAGCGTTCCCTGATA-CCAAGGGTCT 1045
      Allele 2 268 AACCCCCTTG--TCGCCGGTCAAAGCCTCTTCG 298…707 CACAGCCTAATGTGACCGCTT-CGCCGGCGAC 737…1014 GTTTAGTGAGCGTTCCCTGA---CCAAGGGTCT 1043
```

Figure S6. Alignment of the 97 PCR products sequenced against wild-type sequence in the region of the 3 sgRNA for transgenic lines transformed with construct 2 (*p35S::zCas9i*). Wild-type sgRNA sequence is highlighted in grey and all insertions or deletions are highlighted in red. Each allele is represented for all plantlets to highlight monoallelic or biallelic mutations.

```
                                sgRNA 1                                  sgRNA 2                                    sgRNA 3
WT     Allele 1 268 AACCCCCTTGTCGCCGGTCAAAGCCTCTTCG 298…707 CACAGCCTAATGTGACCGCTTCGCCGGCGAC 737…1014 GTTTAGTGAGCGTTCCCTGAT-CCAAGGGTCT 1044
       Allele 2 268 AACCCCCTTGTCGCCGGTCAAAGCCTCTTCG 298…707 CACAGCCTAATGTGACCGCTTCGCCGGCGAC 737…1014 GTTTAGTGAGCGTTCCCTGAT-CCAAGGGTCT 1044
C3_1   Allele 1 268 AACCCCCTTGTCGCCGGTCAAAGCCTCTTCG 298…707 CACAGCCTAATGTGACCGCTTCGCCGGCGAC 737…1014 GTTTAGTGAGCGTTCCCTGATTCCAAGGGTCT 1045
       Allele 2 268 AACCCCCTTGTCGCCGGTCAAAGCCTCTTCG 298…707 CACAGCCTAATGTGACCGCTTCGCCGGCGAC 737…1014 GTTTAGTGAGCGTTCCCTGATTCCAAGGGTCT 1045
C3_2   Allele 1 268 AACCCCCTTGTCGCCGGTCAAAGCCTCTTCG 298…707 CACAGCCTAATGTGACCGCTTCGCCGGCGAC 737…1014 GTTTAGTGAGCGTTCCCTG-T-CCAAGGGTCT 1043
       Allele 2 268 AACCCCCTTGTCGCCGGTCAAAGCCTCTTCG 298…707 CACAGCCTAATGTGACCGCTTCGCCGGCGAC 737…1014 GTTTAGTGAGCGTTCCCTGATTCCAAGGGTCT 1045
C3_3   Allele 1 268 AACCCCCTTGTCGCCGGTCAAAGCCTCTTCG 298…707 CACAGCCTAATGTGACCGCTTCGCCGGCGAC 737…1014 GTTTAGTGAGCGTTCCCTGATTCCAAGGGTCT 1045
       Allele 2 268 AACCCCCTTGTCGCCGGTCAAAGCCTCTTCG 298…707 CACAGCCTAATGTGACCGCTTCGCCGGCGAC 737…1014 GTTTAGTGAGCGTTCCCTGAT-CCAAGGGTCT 1044
C3_4   Allele 1 268 AACCCCCTTGTCGCCGGTCAAAGCCTCTTCG 298…707 CACAGCCTAATGTGACCGCTTCGCCGGCGAC 737…1014 GTTTAGTGAGCGTTCCCTGATTCCAAGGGTCT 1045
       Allele 2 268 AACCCCCTTGTCGCCGGTCAAAGCCTCTTCG 298…707 CACAGCCTAATGTGACCGCTTCGCCGGCGAC 737…1014 GTTTAGTGAGCGTTCCCTGATTCCAAGGGTCT 1045
C3_5   Allele 1 268 AACCCCCTTGTCGCCGGTCAAAGCCTCTTCG 298…707 CACAGCCTAATGTGACCGCTTCGCCGGCGAC 737…1014 GTTTAGTGAGCGTTCCCTGATTCCAAGGGTCT 1045
       Allele 2 268 AACCCCCTTGTCGCCGGTCAAAGCCTCTTCG 298…707 CACAGCCTAATGTGACCGCTTCGCCGGCGAC 737…1014 GTTTAGTGAGCGTTCCCTGAT-CCAAGGGTCT 1044
C3_6   Allele 1 268 AACCCCCTTGTCGCCGGTCAAAGCCTCTTCG 298…707 CACAGCCTAATGTGACCGCTTCGCCGGCGAC 737…1014 GTTTAGTGAGCGTTCCCTGATTCCAAGGGTCT 1045
       Allele 2 268 AACCCCCTTGTCGCCGGTCAAAGCCTCTTCG 298…707 CACAGCCTAATGTGACCGCTTCGCCGGCGAC 737…1014 GTTTAGTGAGCGTTCCCTGAT-CCAAGGGTCT 1044
C3_7   Allele 1 268 AACCCCCTTGTCGCCGGTCAAAGCCTCTTCG 298…707 CACAGCCTAATGTGACCGCTTCGCCGGCGAC 737…1014 GTTTAGTGAGCGTTCCCTGATTCCAAGGGTCT 1045
       Allele 2 268 AACCCCCTTGTCGCCGGTCAAAGCCTCTTCG 298…707 CACAGCCTAATGTGACCGCTTCGCCGGCGAC 737…1014 GTTTAGTGAGCGTTCCCTGAT-CCAAGGGTCT 1044
C3_8   Allele 1 268 AACCCCCTTGTCGCCGGTCAAAGCCTCTTCG 298…707 CACAGCCTAATGTGACCGCTTCGCCGGCGAC 737…1014 GTTTAGTGAGCGTTCCCTGATTCCAAGGGTCT 1045
       Allele 2 268 AACCCCCTTGTCGCCGGTCAAAGCCTCTTCG 298…707 CACAGCCTAATGTGACCGCTTCGCCGGCGAC 737…1014 GTTTAGTGAGCGTTCCCTGAT-CCAAGGGTCT 1044
C3_9   Allele 1 268 AACCCCCTTGTCGCCGGTCAAAGCCTCTTCG 298…707 CACAGCCTAATGTGACCGCTTCGCCGGCGAC 737…1014 GTTTAGTGAGCGTTCCCTGATTCCAAGGGTCT 1045
       Allele 2 268 AACCCCCTTGTCGCCGGTCAAAGCCTCTTCG 298…707 CACAGCCTAATGTGACCGCTTCGCCGGCGAC 737…1014 GTTTAGTGAGCGTTCCCTGATTCCAAGGGTCT 1045
C3_10  Allele 1 268 AACCCCCTTGTCGCCGGTCAAAGCCTCTTCG 298…707 CACAGCCTAATGTGACCGCTTCGCCGGCGAC 737…1014 GTTTAGTGAGCGTTCCCTGATTCCAAGGGTCT 1045
       Allele 2 268 AACCCCCTTGTCGCCGGTCAAAGCCTCTTCG 298…707 CACAGCCTAATGTGACCGCTTCGCCGGCGAC 737…1014 GTTTAGTGAGCGTTCCCTGATTCCAAGGGTCT 1045
C3_11  Allele 1 268 AACCCCCTTGTCGCCGGTCAAAGCCTCTTCG 298…707 CACAGCCTAATGTGACCGCTTCGCCGGCGAC 737…1014 GTTTAGTGAGCGTTCCCTGATTCCAAGGGTCT 1045
       Allele 2 268 AACCCCCTTGTCGCCGGTCAAAGCCTCTTCG 298…707 CACAGCCTAATGTGACCGCTTCGCCGGCGAC 737…1014 GTTTAGTGAGCGTTCCCTGATACCAAGGGTCT 1045
C3_12  Allele 1 268 AACCCCCTTGTCGCCGGTCAAAGCCTCTTCG 298…707 CACAGCCTAATGTGACCGCTTCGCCGGCGAC 737…1014 GTTTAGTGAGCGTTCCCTGATTCCAAGGGTCT 1045
       Allele 2 268 AACCCCCTTGTCGCCGGTCAAAGCCTCTTCG 298…707 CACAGCCTAATGTGACCGCTTCGCCGGCGAC 737…1014 GTTTAGTGAGCGTTCCCTGATTCCAAGGGTCT 1045
C3_13  Allele 1 268 AACCCCCTTGTCGCCGGTCAAAGCCTCTTCG 298…707 CACAGCCTAATGTGACCGCTTCGCCGGCGAC 737…1014 GTTTAGTGAGCGTTCCCTGATTCCAAGGGTCT 1045
       Allele 2 268 AACCCCCTTGTCGCCGGTCAAAGCCTCTTCG 298…707 CACAGCCTAATGTGACCGCTTCGCCGGCGAC 737…1014 GTTTAGTGAGCGTTCCCTGATTCCAAGGGTCT 1045
C3_14  Allele 1 268 AACCCCCTTGTCGCCGGTCAAAGCCTCTTCG 298…707 CACAGCCTAATGTGACCGCTTCGCCGGCGAC 737…1014 GTTTAGTGAGCGTTCCCTGATTCCAAGGGTCT 1045
       Allele 2 268 AACCCCCTTGTCGCCGGTCAAAGCCTCTTCG 298…707 CACAGCCTAATGTGACCGCTTCGCCGGCGAC 737…1014 GTTTAGTGAGCGTTCC------CCAAGGGTCT 1039
C3_15  Allele 1 268 AACCCCCTTGTCGCCGGTCAAAGCCTCTTCG 298…707 CACAGCCTAATGTGACCGCTTCGCCGGCGAC 737…1014 GTTTAGTGAGCGTTCCCTGATTCCAAGGGTCT 1045
       Allele 2 268 AACCCCCTTGTCGCCGGTCAAAGCCTCTTCG 298…707 CACAGCCTAATGTGACCGCTTCGCCGGCGAC 737…1014 GTTTAGTGAGCGTTCCCTGAT-CCAAGGGTCT 1044
C3_16  Allele 1 268 AACCCCCTTGTCGCCGGTCAAAGCCTCTTCG 298…707 CACAGCCTAATGTGACCGCTTCGCCGGCGAC 737…1014 GTTTAGTGAGCGTTCCCTGATTCCAAGGGTCT 1045
       Allele 2 268 AACCCCCTTGTCGCCGGTCAAAGCCTCTTCG 298…707 CACAGCCTAATGTGACCGCTTCGCCGGCGAC 737…1014 GTTTAGTGAGCGTTCCCTGATTCCAAGGGTCT 1045
C3_17  Allele 1 268 AACCCCCTTGTCGCCGGTCAAAGCCTCTTCG 298…707 CACAGCCTAATGTGACCGCTTCGCCGGCGAC 737…1014 GTTTAGTGAGCGTTCCCTGATTCCAAGGGTCT 1045
       Allele 2 268 AACCCCCTTGTCGCCGGTCAAAGCCTCTTCG 298…707 CACAGCCTAATGTGACCGCTTCGCCGGCGAC 737…1014 GTTTAGTGAGCGTTCCCTGA--CCAAGGGTCT 1044
C3_18  Allele 1 268 AACCCCCTTGTCGCCGGTCAAAGCCTCTTCG 298…707 CACAGCCTAATGTGACCGCTTCGCCGGCGAC 737…1014 GTTTAGTGAGCGTTCCCTGATTCCAAGGGTCT 1045
       Allele 2 268 AACCCCCTTGTCGCCGGTCAAAGCCTCTTCG 298…707 CACAGCCTAATGTGACCGCTTCGCCGGCGAC 737…1014 GTTTAGTGAGCGTTCCCTGA--CCAAGGGTCT 1044
C3_19  Allele 1 268 AACCCCCTTGTCGCCGGTCAAAGCCTCTTCG 298…707 CACAGCCTAATGTGACCGCTTCGCCGGCGAC 737…1014 GTTTAGTGAGCGTTCCCTGATTCCAAGGGTCT 1045
       Allele 2 268 AACCCCCTTGTCGCCGGTCAAAGCCTCTTCG 298…707 CACAGCCTAATGTGACCGCTTCGCCGGCGAC 737…1014 GTTTAGTGAGCGTTCCCTGAT-CCAAGGGTCT 1044
C3_20  Allele 1 268 AACCCCCTTGTCGCCGGTCAAAGCCTCTTCG 298…707 CACAGCCTAATGTGACCGCTTCGCCGGCGAC 737…1014 GTTTAGTGAGCGTTCCCTGATTCCAAGGGTCT 1045
       Allele 2 268 AACCCCCTTGTCGCCGGTCAAAGCCTCTTCG 298…707 CACAGCCTAATGTGACCGCTTCGCCGGCGAC 737…1014 GTTTAGTGAGCGT---------CCAAGGGTCT 1036
C3_21  Allele 1 268 AACCCCCTTGTCGCCGGTCAAAGCCTCTTCG 298…707 CACAGCCTAATGTGACCGCTTCGCCGGCGAC 737…1014 GTTTAGTGAGCGTTCCCTGATTCCAAGGGTCT 1045
       Allele 2 268 AACCCCCTTGTCGCCGGTCAAAGCCTCTTCG 298…707 CACAGCCTAATGTGACCGCTTCGCCGGCGAC 737…1014 GTTTAGTGAGCGTTCCCTGAT-CCAAGGGTCT 1044
C3_22  Allele 1 268 AACCCCCTTGTCGCCGGTCAAAGCCTCTTCG 298…707 CACAGCCTAATGTGACCGCTTCGCCGGCGAC 737…1014 GTTTAGTGAGCGTTCCCTGATTCCAAGGGTCT 1045
       Allele 2 268 AACCCCCTTGTCGCCGGTCAAAGCCTCTTCG 298…707 CACAGCCTAATGTGACCGCTTCGCCGGCGAC 737…1014 GTTTAGTGAGCGTTCCCTGATTCCAAGGGTCT 1045
C3_23  Allele 1 268 AACCCCCTTGTCGCCGGTCAAAGCCTCTTCG 298…707 CACAGCCTAATGTGACCGCTTCGCCGGCGAC 737…1014 GTTTAGTGAGCGTTCCCTGATTCCAAGGGTCT 1045
       Allele 2 268 AACCCCCTTGTCGCCGGTCAAAGCCTCTTCG 298…707 CACAGCCTAATGTGACCGCTTCGCCGGCGAC 737…1014 GTTTAGTGAGCGTTCCCTGAT-CCAAGGGTCT 1044
C3_24  Allele 1 268 AACCCCCTTGTCGCCGGTCAAAGCCTCTTCG 298…707 CACAGCCTAATGTGACCGCTTCGCCGGCGAC 737…1014 GTTTAGTGAGCGTTCCCTGATTCCAAGGGTCT 1045
       Allele 2 268 AACCCCCTTGTCGCCGGTCAAAGCCTCTTCG 298…707 CACAGCCTAATGTGACCGCTTCGCCGGCGAC 737…1014 GTTTAGTGAGCGTTCCCTGATTCCAAGGGTCT 1045
C3_25  Allele 1 268 AACCCCCTTGTCGCCGGTCAAAGCCTCTTCG 298…707 CACAGCCTAATGTGACCGCTTCGCCGGCGAC 737…1014 GTTTAGTGAGCGTTCCCTGATTCCAAGGGTCT 1045
       Allele 2 268 AACCCCCTTGTCGCCGGTCAAAGCCTCTTCG 298…707 CACAGCCTAATGTGACCGCTTCGCCGGCGAC 737…1014 GTTTAGTGAGCGTTCCCTGATTCCAAGGGTCT 1045
C3_26  Allele 1 268 AACCCCCTTGTCGCCGGTCAAAGCCTCTTCG 298…707 CACAGCCTAATGTGACCGCTTCGCCGGCGAC 737…1014 GTTTAGTGAGCGTTCCCTGATTCCAAGGGTCT 1045
       Allele 2 268 AACCCCCTTGTCGCCGGTCAAAGCCTCTTCG 298…707 CACAGCCTAATGTGACCGCTTCGCCGGCGAC 737…1014 GTTTAGTGAGCGTTCCCTGATTCCAAGGGTCT 1045
C3_27  Allele 1 268 AACCCCCTTGTCGCCGGTCAAAGCCTCTTCG 298…707 CACAGCCTAATGTGACCGCTTCGCCGGCGAC 737…1014 GTTTAGTGAGCGTTCCCTGATTCCAAGGGTCT 1045
       Allele 2 268 AACCCCCTTGTCGCCGGTCAAAGCCTCTTCG 298…707 CACAGCCTAATGTGACCGCTTCGCCGGCGAC 737…1014 GTTTAGTGAGCGTTCCCTGATTCCAAGGGTCT 1045
C3_28  Allele 1 268 AACCCCCTTGTCGCCGGTCAAAGCCTCTTCG 298…707 CACAGCCTAATGTGACCGCTTCGCCGGCGAC 737…1014 GTTTAGTGAGCGTTCCCTGATTCCAAGGGTCT 1045
       Allele 2 268 AACCCCCTTGTCGCCGGTCAAAGCCTCTTCG 298…707 CACAGCCTAATGTGACCGCTTCGCCGGCGAC 737…1014 GTTTAGTGAGCGTTCCCTGAT-CCAAGGGTCT 1044
C3_29  Allele 1 268 AACCCCCTTGTCGCCGGTCAAAGCCTCTTCG 298…707 CACAGCCTAATGTGACCGCTTCGCCGGCGAC 737…1014 GTTTAGTGAGCGTTCCCTGATTCCAAGGGTCT 1045
       Allele 2 268 AACCCCCTTGTCGCCGGTCAAAGCCTCTTCG 298…707 CACAGCCTAATGTGACCGCTTCGCCGGCGAC 737…1014 GTTTAGTGAGCGTTCCCTGAT-CCAAGGGTCT 1044
C3_30  Allele 1 268 AACCCCCTTGTCGCCGGTCAAAGCCTCTTCG 298…707 CACAGCCTAATGTGACCGCTTCGCCGGCGAC 737…1014 GTTTAGTGAGCGTTCCCTGATTCCAAGGGTCT 1045
       Allele 2 268 AACCCCCTTGTCGCCGGTCAAAGCCTCTTCG 298…707 CACAGCCTAATGTGACCGCTTCGCCGGCGAC 737…1014 GTTTAGTGAGCGTTCCCTGATACCAAGGGTCT 1045
C3_31  Allele 1 268 AACCCCCTTGTCGCCCGTCAAAGCCTCTTCG 298…707 CACAGCCTAATGTGACCGCTTCGCCGGCGAC 737…1014 GTTTAGTGAGCGTTCCCTGATTCCAAGGGTCT 1045
       Allele 2 268 AACCCCCTTGTCGCCCGTCAAAGCCTCTTCG 298…707 CACAGCCTAATGTGACCGCTTCGCCGGCGAC 737…1014 GTTTAGTGAGCGTTCCCTGAT-CCAAGGGTCT 1044
C3_32  Allele 1 268 AACCCCCTTGTCGCCGGTCAAAGCCTCTTCG 298…707 CACAGCCTAATGTGACCGCTTCGCCGGCGAC 737…1014 GTTTAGTGAGCGTTCCCTGATTCCAAGGGTCT 1045
       Allele 2 268 AACCCCCTTGTCGCCGGTCAAAGCCTCTTCG 298…707 CACAGCCTAATGTGACCGCTTCGCCGGCGAC 737…1014 GTTTAGTGAGCGTTCCCTG-T-CCAAGGGTCT 1043
C3_33  Allele 1 268 AACCCCCTTGTCGCCGGTCAAAGCCTCTTCG 298…707 CACAGCCTAATGTGACCGCTTCGCCGGCGAC 737…1014 GTTTAGTGAGCGTTCCCTGATTCCAAGGGTCT 1045
       Allele 2 268 AACCCCCTTGTCGCCGGTCAAAGCCTCTTCG 298…707 CACAGCCTAATGTGACCGCTTCGCCGGCGAC 737…1014 GTTTAGTGAGCGTTCCCTGATTCCAAGGGTCT 1045
C3_34  Allele 1 268 AACCCCCTTGTCGCCGGTCAAAGCCTCTTCG 298…707 CACAGCCTAATGTGACCGCTTCGCCGGCGAC 737…1014 GTTTAGTGAGCGTTCCCTGATTCCAAGGGTCT 1045
       Allele 2 268 AACCCCCTTGTCGCCGGTCAAAGCCTCTTCG 298…707 CACAGCCTAATGTGACCGCTTCGCCGGCGAC 737…1014 GTTTAGTGAGCGTTCCCTGATTCCAAGGGTCT 1045
C3_35  Allele 1 268 AACCCCCTTGTCGCCGGTCAAAGCCTCTTCG 298…707 CACAGCCTAATGTGACCGCTTCGCCGGCGAC 737…1014 GTTTAGTGAGCGTTCCCTGATTCCAAGGGTCT 1045
       Allele 2 268 AACCCCCTTGTCGCCGGTCAAAGCCTCTTCG 298…707 CACAGCCTAATGTGACCGCTTCGCCGGCGAC 737…1014 GTTTAGTGAGCGTTCCCTGATTCCAAGGGTCT 1045
C3_36  Allele 1 268 AACCCCCTTGTCGCCGGTCAAAGCCTCTTCG 298…707 CACAGCCTAATGTGACCGCTTCGCCGGCGAC 737…1014 GTTTAGTGAGCGTTCCCTGATTCCAAGGGTCT 1045
       Allele 2 268 AACCCCCTTGTCGCCGGTCAAAGCCTCTTCG 298…707 CACAGCCTAATGTGACCGCTTCGCCGGCGAC 737…1014 GTTTAGTGAGCGTTCCCTGATTCCAAGGGTCT 1045
C3_37  Allele 1 268 AACCCCCTTGTCGCCGGTCAAAGCCTCTTCG 298…707 CACAGCCTAATGTGACCGCTTCGCCGGCGAC 737…1014 GTTTAGTGAGCGTTCCCTGATTCCAAGGGTCT 1045
       Allele 2 268 AACCCCCTTGTCGCCGGTCAAAGCCTCTTCG 298…707 CACAGCCTAATGTGACCGCTTCGCCGGCGAC 737…1014 GTTTAGTGAGCGTTCCCTGATTCCAAGGGTCT 1045
C3_38  Allele 1 268 AACCCCCTTGTCGCCGGTCAAAGCCTCTTCG 298…707 CACAGCCTAATGTGACCGCTTCGCCGGCGAC 737…1014 GTTTAGTGAGCGTTCCCTGATTCCAAGGGTCT 1045
       Allele 2 268 AACCCCCTTGTCGCCGGTCAAAGCCTCTTCG 298…707 CACAGCCTAATGTGACCGCTTCGCCGGCGAC 737…1014 GTTTAGTGAGCGTTCCCTGAT-CCAAGGGTCT 1044
C3_39  Allele 1 268 AACCCCCTTGTCGCCGGTCAAAGCCTCTTCG 298…707 CACAGCCTAATGTGACCGCTTCGCCGGCGAC 737…1014 GTTTAGTGAGCGTTCCCTGATTCCAAGGGTCT 1045
       Allele 2 268 AACCCCCTTGTCGCCGGTCAAAGCCTCTTCG 298…707 CACAGCCTAATGTGACCGCTTCGCCGGCGAC 737…1014 GTTTAGTGAGCGTTCCCTGAT-CCAAGGGTCT 1044
C3_40  Allele 1 268 AACCCCCTTGTCGCCGGTCAAAGCCTCTTCG 298…707 CACAGCCTAATGTGACCGCTTCGCCGGCGAC 737…1014 GTTTAGTGAGCGTTCCCTGATTCCAAGGGTCT 1045
       Allele 2 268 AACCCCCTTGTCGCCGGTCAAAGCCTCTTCG 298…707 CACAGCCTAATGTGACCGCTTCGCCGGCGAC 737…1014 GTTTAGTGAGCGTTCCCTGATTCCAAGGGTCT 1045
C3_41  Allele 1 268 AACCCCCTTGTCGCCGGTCAAAGCCTCTTCG 298…707 CACAGCCTAATGTGACCGCTTCGCCGGCGAC 737…1014 GTTTAGTGAGCGTTCCCTGATTCCAAGGGTCT 1045
       Allele 2 268 AACCCCCTTGTCGCCGGTCAAAGCCTCTTCG 298…707 CACAGCCTAATGTGACCGCTTCGCCGGCGAC 737…1014 GTTTAGTGAGCGTTCCCTGATTCCAAGGGTCT 1045
C3_42  Allele 1 268 AACCCCCTTGTCGCCGGTCAAAGCCTCTTCG 298…707 CACAGCCTAATGTGACCGCTTCGCCGGCGAC 737…1014 GTTTAGTGAGCGTTCCCTGATTCCAAGGGTCT 1045
       Allele 2 268 AACCCCCTTGTCGCCGGTCAAAGCCTCTTCG 298…707 CACAGCCTAATGTGACCGCTTCGCCGGCGAC 737…1014 GTTTAGTGAGCGTTCCCTGAT-CCAAGGGTCT 1044
```

```
WT    Allele 1 268 AACCCCCTTGTCGCCGGTCAAAGCCTCTTCG 298…707 CACAGCCTAATGTGACCGCTTCGCCGGCGAC 737…1014 GTTTAGTGAGCGTTCCCTGAT-CCAAGGGTCT 1044
      Allele 2 268 AACCCCCTTGTCGCCGGTCAAAGCCTCTTCG 298…707 CACAGCCTAATGTGACCGCTTCGCCGGCGAC 737…1014 GTTTAGTGAGCGTTCCCTGAT-CCAAGGGTCT 1044
C3_43 Allele 1 268 AACCCCCTTGTCGCCGGTCAAAGCCTCTTCG 298…707 CACAGCCTAATGTGACCGCTTCGCCGGCGAC 737…1014 GTTTAGTGAGCGTTCCCTGATTCCAAGGGTCT 1045
      Allele 2 268 AACCCCCTTGTCGCCGGTCAAAGCCTCTTCG 298…707 CACAGCCTAATGTGACCGCTTCGCCGGCGAC 737…1014 GTTTAGTGAGCGTTCCCTGATTCCAAGGGTCT 1045
C3_44 Allele 1 268 AACCCCCTTGTCGCCGGTCAAAGCCTCTTCG 298…707 CACAGCCTAATGTGACCGCTTCGCCGGCGAC 737…1014 GTTTAGTGAGCGTTCCCTGATTCCAAGGGTCT 1045
      Allele 2 268 AACCCCCTTGTCGCCGGTCAAAGCCTCTTCG 298…707 CACAGCCTAATGTGACCGCTTCGCCGGCGAC 737…1014 GTTTAGTGAGCGTTCCCTGATTCCAAGGGTCT 1045
C3_45 Allele 1 268 AACCCCCTTGTCGCCGGTCAAAGCCTCTTCG 298…707 CACAGCCTAATGTGACCGCTTCGCCGGCGAC 737…1014 GTTTAGTGAGCGTTCCCTGATTCCAAGGGTCT 1045
      Allele 2 268 AACCCCCTTGTCGCCGGTCAAAGCCTCTTCG 298…707 CACAGCCTAATGTGACCGCTTCGCCGGCGAC 737…1014 GTTTAGTGAGCGTTCCCTGA--CCAAGGGTCT 1043
C3_46 Allele 1 268 AACCCCCTTGTCGCCGGTCAAAGCCTCTTCG 298…707 CACAGCCTAATGTGACCGCTTCGCCGGCGAC 737…1014 GTTTAGTGAGCGTTCCCTGATTCCAAGGGTCT 1045
      Allele 2 268 AACCCCCTTGTCGCCGGTCAAAGCCTCTTCG 298…707 CACAGCCTAATGTGACCGCTTCGCCGGCGAC 737…1014 GTTTAGTGAGCGTTCCCTGATTCCAAGGGTCT 1045
C3_47 Allele 1 268 AACCCCCTTGTCGCCGGTCAAAGCCTCTTCG 298…707 CACAGCCTAATGTGACCGCTTCGCCGGCGAC 737…1014 GTTTAGTGAGCGTTCCCTG-T-CCAAGGGTCT 1043
      Allele 2 268 AACCCCCTTGTCGCCGGTCAAAGCCTCTTCG 298…707 CACAGCCTAATGTGACCGCTTCGCCGGCGAC 737…1014 GTTTAGTGAGCGTTCCCTGATTCCAAGGGTCT 1045
C3_48 Allele 1 268 AACCCCCTTGTCGCCGGTCAAAGCCTCTTCG 298…707 CACAGCCTAATGTGACCGCTTCGCCGGCGAC 737…1014 GTTTAGTGAGCGTTCCCTGATTCCAAGGGTCT 1045
      Allele 2 268 AACCCCCTTGTCGCCGGTCAAAGCCTCTTCG 298…707 CACAGCCTAATGTGACCGCTTCGCCGGCGAC 737…1014 GTTTAGTGAGCGTTCCCTGAT-CCAAGGGTCT 1044
C3_49 Allele 1 268 AACCCCCTTGTCGCCGGTCAAAGCCTCTTCG 298…707 CACAGCCTAATGTGACCGCTTCGCCGGCGAC 737…1014 GTTTAGTGAGCGTTCCCTGATTCCAAGGGTCT 1045
      Allele 2 268 AACCCCCTTGTCGCCGGTCAAAGCCTCTTCG 298…707 CACAGCCTAATGTGACCGCTTCGCCGGCGAC 737…1014 GTTTAGTGAGCGT---------CCAAGGGTCT 1036
C3_50 Allele 1 268 AACCCCCTTGTCGCCGGTCAAAGCCTCTTCG 298…707 CACAGCCTAATGTGACCGCTTCGCCGGCGAC 737…1014 GTTTAGTGAGCGTTCCCTGATTCCAAGGGTCT 1045
      Allele 2 268 AACCCCCTTGTCGCCGGTCAAAGCCTCTTCG 298…707 CACAGCCTAATGTGACCGCTTCGCCGGCGAC 737…1014 GTTTAGTGAGCGTTCCCTGAT-CCAAGGGTCT 1044
C3_51 Allele 1 268 AACCCCCTTGTCGCCGGTCAAAGCCTCTTCG 298…707 CACAGCCTAATGTGACCGCTTCGCCGGCGAC 737…1014 GTTTAGTGAGCGTTCCCTGATTCCAAGGGTCT 1045
      Allele 2 268 AACCCCCTTGTCGCCGGTCAAAGCCTCTTCG 298…707 CACAGCCTAATGTGACCGCTTCGCCGGCGAC 737…1014 GTTTAGTGAGCGTTCCCTGATTCCAAGGGTCT 1045
C3_52 Allele 1 268 AACCCCCTTGTCGCCGGTCAAAGCCTCTTCG 298…707 CACAGCCTAATGTGACCGCTTCGCCGGCGAC 737…1014 GTTTAGTGAGCGTTCCCTGATTCCAAGGGTCT 1045
      Allele 2 268 AACCCCCTTGTCGCCGGTCAAAGCCTCTTCG 298…707 CACAGCCTAATGTGACCGCTTCGCCGGCGAC 737…1014 GTTTAGTGAGCGTTCCCTGATTCCAAGGGTCT 1045
C3_53 Allele 1 268 AACCCCCTTGTCGCCGGTCAAAGCCTCTTCG 298…707 CACAGCCTAATGTGACCGCTTCGCCGGCGAC 737…1014 GTTTAGTGAGCGTTCCCTG-T-CCAAGGGTCT 1043
      Allele 2 268 AACCCCCTTGTCGCCGGTCAAAGCCTCTTCG 298…707 CACAGCCTAATGTGACCGCTTCGCCGGCGAC 737…1014 GTTTAGTGAGCGTTCCCTGATTCCAAGGGTCT 1045
C3_54 Allele 1 268 AACCCCCTTGTCGCCGGTCAAAGCCTCTTCG 298…707 CACAGCCTAATGTGACCGCTTCGCCGGCGAC 737…1014 GTTTAGTGAGCGTTCCCTGATTCCAAGGGTCT 1045
      Allele 2 268 AACCCCCTTGTCGCCGGTCAAAGCCTCTTCG 298…707 CACAGCCTAATGTGACCGCTTCGCCGGCGAC 737…1014 GTTTAGTGAGCGTTCCCTGATTCCAAGGGTCT 1045
C3_55 Allele 1 268 AACCCCCTTGTCGCCGGTCAAAGCCTCTTCG 298…707 CACAGCCTAATGTGACCGCTTCGCCGGCGAC 737…1014 GTTTAGTGAGCGTTCCCTGATTCCAAGGGTCT 1045
      Allele 2 268 AACCCCCTTGTCGCCGGTCAAAGCCTCTTCG 298…707 CACAGCCTAATGTGACCGCTTCGCCGGCGAC 737…1014 GTTTAGTGAGCGTTCCCTGAT-CCAAGGGTCT 1044
C3_56 Allele 1 268 AACCCCCTTGTCGCCGGTCAAAGCCTCTTCG 298…707 CACAGCCTAATGTGACCGCTTCGCCGGCGAC 737…1014 GTTTAGTGAGCGTTCCCTGATTCCAAGGGTCT 1045
      Allele 2 268 AACCCCCTTGTCGCCGGTCAAAGCCTCTTCG 298…707 CACAGCCTAATGTGACCGCTTCGCCGGCGAC 737…1014 GTTTAGTGAGCGTTCCCTGATTCCAAGGGTCT 1045
C3_57 Allele 1 268 AACCCCCTTGTCGCCGGTCAAAGCCTCTTCG 298…707 CACAGCCTAATGTGACCGCTTCGCCGGCGAC 737…1014 GTTTAGTGAGCGTTCCCTGATTCCAAGGGTCT 1045
      Allele 2 268 AACCCCCTTGTCGCCGGTCAAAGCCTCTTCG 298…707 CACAGCCTAATGTGACCGCTTCGCCGGCGAC 737…1014 GTTTAGTGAGCGTTCCCTGA--CCAAGGGTCT 1044
C3_58 Allele 1 268 AACCCCCTTGTCGCCGGTCAAAGCCTCTTCG 298…707 CACAGCCTAATGTGACCGCTTCGCCGGCGAC 737…1014 GTTTAGTGAGCGTTCCCTGATTCCAAGGGTCT 1045
      Allele 2 268 AACCCCCTTGTCGCCGGTCAAAGCCTCTTCG 298…707 CACAGCCTAATGTGACCGCTTCGCCGGCGAC 737…1014 GTTTAGTGAGCGTTCCCTGAT-CCAAGGGTCT 1044
C3_59 Allele 1 268 AACCCCCTTGTCGCCGGTCAAAGCCTCTTCG 298…707 CACAGCCTAATGTGACCGCTTCGCCGGCGAC 737…1014 GTTTAGTGAGCGTTCCCTGATTCCAAGGGTCT 1045
      Allele 2 268 AACCCCCTTGTCGCCGGTCAAAGCCTCTTCG 298…707 CACAGCCTAATGTGACCGCTTCGCCGGCGAC 737…1014 GTTTAGTGAGCGTTCCCTGATTCCAAGGGTCT 1045
C3_60 Allele 1 268 AACCCCCTTGTCGCCGGTCAAAGCCTCTTCG 298…707 CACAGCCTAATGTGACCGCTTCGCCGGCGAC 737…1014 GTTTAGTGAGCGTTCCCTGATTCCAAGGGTCT 1045
      Allele 2 268 AACCCCCTTGTCGCCGGTCAAAGCCTCTTCG 298…707 CACAGCCTAATGTGACCGCTTCGCCGGCGAC 737…1014 GTTTAGTGAGCGTTCCCTGATTCCAAGGGTCT 1045
C3_61 Allele 1 268 AACCCCCTTGTCGCCGGTCAAAGCCTCTTCG 298…707 CACAGCCTAATGTGACCGCTTCGCCGGCGAC 737…1014 GTTTAGTGAGCGTTCCCTGATTCCAAGGGTCT 1045
      Allele 2 268 AACCCCCTTGTCGCCGGTCAAAGCCTCTTCG 298…707 CACAGCCTAATGTGACCGCTTCGCCGGCGAC 737…1014 GTTTAGTGAGCGTTCCCTGATTCCAAGGGTCT 1045
C3_62 Allele 1 268 AACCCCCTTGTCGCCGGTCAAAGCCTCTTCG 298…707 CACAGCCTAATGTGACCGCTTCGCCGGCGAC 737…1014 GTTTAGTGAGCGTTCCCTG-T-CCAAGGGTCT 1043
      Allele 2 268 AACCCCCTTGTCGCCGGTCAAAGCCTCTTCG 298…707 CACAGCCTAATGTGACCGCTTCGCCGGCGAC 737…1014 GTTTAGTGAGCGTTCCCTGATTCCAAGGGTCT 1045
C3_63 Allele 1 268 AACCCCCTTGTCGCCGGTCAAAGCCTCTTCG 298…707 CACAGCCTAATGTGACCGCTTCGCCGGCGAC 737…1014 GTTTAGTGAGCGTTCCCTGATTCCAAGGGTCT 1045
      Allele 2 268 AACCCCCTTGTCGCCGGTCAAAGCCTCTTCG 298…707 CACAGCCTAATGTGACCGCTTCGCCGGCGAC 737…1014 GTTTAGTGAGCGTTCCCTGAT-CCAAGGGTCT 1044
C3_64 Allele 1 268 AACCCCCTTGTCGCCGGTCAAAGCCTCTTCG 298…707 CACAGCCTAATGTGACCGCTTCGCCGGCGAC 737…1014 GTTTAGTGAGCGTTCCCTGATTCCAAGGGTCT 1045
      Allele 2 268 AACCCCCTTGTCGCCGGTCAAAGCCTCTTCG 298…707 CACAGCCTAATGTGACCGCTTCGCCGGCGAC 737…1014 GTTTAGTGAGCGTTCCCTGAT-CCAAGGGTCT 1044
C3_65 Allele 1 268 AACCCCCTTGTCGCCGGTCAAAGCCTCTTCG 298…707 CACAGCCTAATGTGACCGCTTCGCCGGCGAC 737…1014 GTTTAGTGAGCGTTCCCTG-T-CCAAGGGTCT 1043
      Allele 2 268 AACCCCCTTGTCGCCGGTCAAAGCCTCTTCG 298…707 CACAGCCTAATGTGACCGCTTCGCCGGCGAC 737…1014 GTTTAGTGAGCGTTCCCTGATTCCAAGGGTCT 1045
C3_66 Allele 1 268 AACCCCCTTGTCGCCGGTCAAAGCCTCTTCG 298…707 CACAGCCTAATGTGACCGCTTCGCCGGCGAC 737…1014 GTTTAGTGAGCGTTCCCTGATTCCAAGGGTCT 1045
      Allele 2 268 AACCCCCTTGTCGCCGGTCAAAGCCTCTTCG 298…707 CACAGCCTAATGTGACCGCTTCGCCGGCGAC 737…1014 GTTTAGTGAGCGTTCCCTGATTCCAAGGGTCT 1045
C3_67 Allele 1 268 AACCCCCTTGTCGCCGGTCAAAGCCTCTTCG 298…707 CACAGCCTAATGTGACCGCTTCGCCGGCGAC 737…1014 GTTTAGTGAGCGTTCCCTGATTCCAAGGGTCT 1045
      Allele 2 268 AACCCCCTTGTCGCCGGTCAAAGCCTCTTCG 298…707 CACAGCCTAATGTGACCGCTTCGCCGGCGAC 737…1014 GTTTAGTGAGCGTTCCCTGATTCCAAGGGTCT 1045
C3_68 Allele 1 268 AACCCCCTTGTCGCCGGTCAAAGCCTCTTCG 298…707 CACAGCCTAATGTGACCGCTTCGCCGGCGAC 737…1014 GTTTAGTGAGCGTTCCCTGATTCCAAGGGTCT 1045
      Allele 2 268 AACCCCCTTGTCGCCGGTCAAAGCCTCTTCG 298…707 CACAGCCTAATGTGACCGCTTCGCCGGCGAC 737…1014 GTTTAGTGAGCGTTCCCTGAT--CAAGGGTCT 1043
C3_69 Allele 1 268 AACCCCCTTGTCGCCGGTCAAAGCCTCTTCG 298…707 CACAGCCTAATGTGACCGCTTCGCCGGCGAC 737…1014 GTTTAGTGAGCGTTCCCTGATTCCAAGGGTCT 1045
      Allele 2 268 AACCCCCTTGTCGCCGGTCAAAGCCTCTTCG 298…707 CACAGCCTAATGTGACCGCTTCGCCGGCGAC 737…1014 GTTTAGTGAGCGTTCCCTGATTCCAAGGGTCT 1045
C3_70 Allele 1 268 AACCCCCTTGTCGCCGGTCAAAGCCTCTTCG 298…707 CACAGCCTAATGTGACCGCTTCGCCGGCGAC 737…1014 GTTTAGTGAGCGTTCCCTGATTCCAAGGGTCT 1045
      Allele 2 268 AACCCCCTTGTCGCCGGTCAAAGCCTCTTCG 298…707 CACAGCCTAATGTGACCGCTTCGCCGGCGAC 737…1014 GTTTAGTGAGCGTTCCCTGA--CCAAGGGTCT 1043
C3_71 Allele 1 268 AACCCCCTTGTCGCCGGTCAAAGCCTCTTCG 298…707 CACAGCCTAATGTGACCGCTTCGCCGGCGAC 737…1014 GTTTAGTGAGCGTTCCCTGATTCCAAGGGTCT 1045
      Allele 2 268 AACCCCCTTGTCGCCGGTCAAAGCCTCTTCG 298…707 CACAGCCTAATGTGACCGCTTCGCCGGCGAC 737…1014 GTTTAGTGAGCGTTCCCTGAT-CCAAGGGTCT 1044
```

Figure S7. Alignment of the 71 PCR products sequenced against wild-type sequence in the region of the 3 sgRNA for construct 3 (*pRPS5a::zCas9i*). Wild-type sgRNA sequence is highlighted in grey and all insertions or deletions are highlighted in red. Each allele is represented for all plantlets to highlight monoallelic or biallelic mutations.

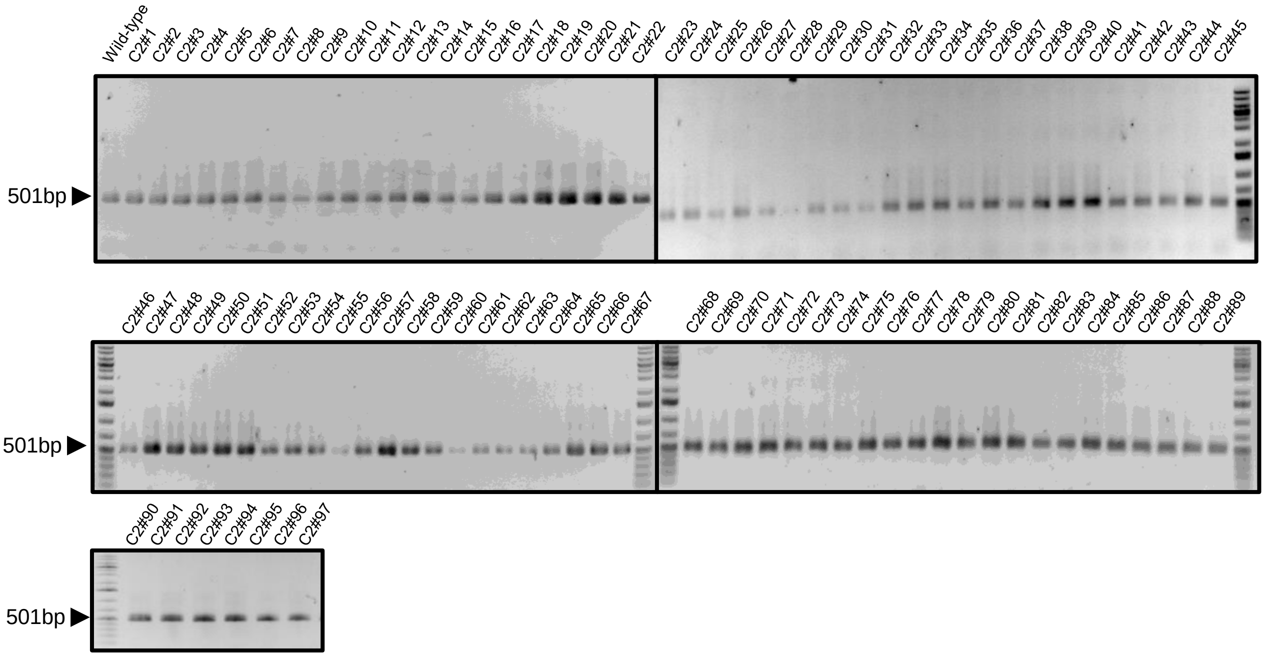

Figure S8. Genotyping of 97 selected plantlets for off-target 1 analysis with transgenic plantlets expressing construct 2 (*p35S::zCas9i*). PCR amplification of a region including off-target 1 sequence (501bp) in 97 independent plantlets showing the presence of T-DNA insertion.

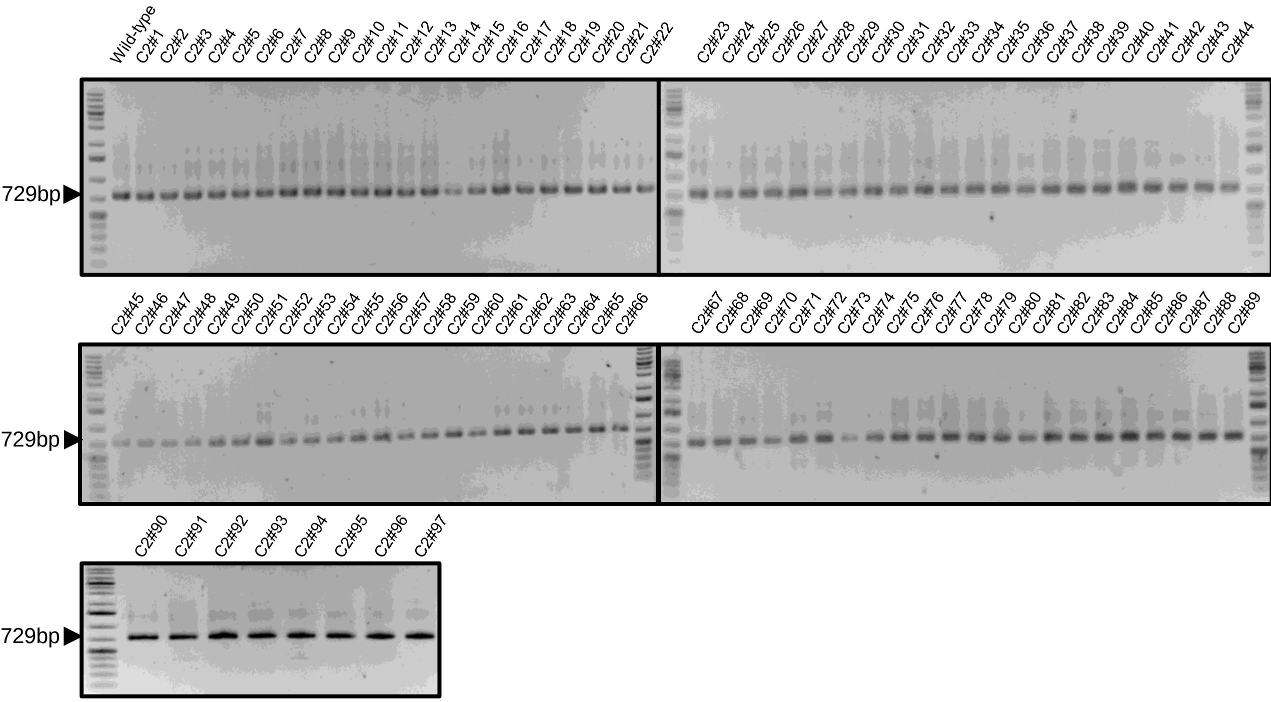

Figure S9. Genotyping of 97 selected plantlets for off-target 3 analysis with transgenic plantlets expressing construct 2 (*p35S::zCas9i*). PCR amplification of a region including off-target 3 sequence (729bp) in 97 independent plantlets showing the presence of T-DNA insertion.

WILD-TYPE off-target 1	TGCTCCGATTCC**GAGGATGGCGCCGGCGACAA**TGGCAAAGCC
Construct2_1 off-target 1	TGCTCCGATTCC**GAGGATGGCGCCGGCGACAA**TGGCGAAGCC
Construct2_2 off-target 1	TGCTCCGATTCC**GAGGATGGCGCCGGCGACAA**TGGCAAAGCC
Construct2_3 off-target 1	TGCTCCGATTCC**GAGGATGGCGCCGGCGACAA**TGGCAAAGCC
Construct2_4 off-target 1	TGCTCCGATTCC**GAGGATGGCGCCGGCGACAA**TGGCGAAGCC
Construct2_5 off-target 1	TGCTCCGATTCC**GAGGATGGCGCCGGCGACAA**TGGCAAAGCC
Construct2_6 off-target 1	TGCTCCGATTCC**GAGGATGGCGCCGGCGACAA**TGGCGAAGCC
Construct2_7 off-target 1	TGCTCCGATTCC**GAGGATGGCGCCGGCGACAA**TGGCGAAGCC
Construct2_8 off-target 1	TGCTCCGATTCC**GAGGATGGCGCCGGCGACAA**TGGCAAAGCC
Construct2_9 off-target 1	TGCTCCGATTCC**GAGGATGGCGCCGGCGACAA**TGGCAAAGCC
Construct2_10 off-target 1	TGCTCCGATTCC**GAGGATGGCGCCGGCGACAA**TGGCGAAGCC
Construct2_11 off-target 1	TGCTCCGATTCC**GAGGATGGCGCCGGCGACAA**TGGCGAAGCC
Construct2_12 off-target 1	TGCTCCGATTCC**GAGGATGGCGCCGGCGACAA**TGGCGAAGCC
Construct2_13 off-target 1	TGCTCCGATTCC**GAGGATGGCGCCGGCGACAA**TGGCAAAGCC
Construct2_14 off-target 1	TGCTCCGATTCC**GAGGATGGCGCCGGCGACAA**TGGCGAAGCC
Construct2_15 off-target 1	TGCTCCGATTCC**GAGGATGGCGCCGGCGACAA**TGGCGAAGCC
Construct2_16 off-target 1	TGCTCCGATTCC**GAGGATGGCGCCGGCGACAA**TGGCGAAGCC
Construct2_17 off-target 1	TGCTCCGATTCC**GAGGATGGCGCCGGCGACAA**TGGCAAAGCC
Construct2_18 off-target 1	TGCTCCGATTCC**GAGGATGGCGCCGGCGACAA**TGGCGAAGCC
Construct2_19 off-target 1	TGCTCCGATTCC**GAGGATGGCGCCGGCGACAA**TGGCAAAGCC
Construct2_20 off-target 1	TGCTCCGATTCC**GAGGATGGCGCCGGCGACAA**TGGCGAAGCC
Construct2_21 off-target 1	TGCTCCGATTCC**GAGGATGGCGCCGGCGACAA**TGGCGAAGCC
Construct2_22 off-target 1	TGCTCCGATTCC**GAGGATGGCGCCGGCGACAA**TGGCGAAGCC
Construct2_23 off-target 1	TGCTCCGATTCC**GAGGATGGCGCCGGCGACAA**TGGCGAAGCC
Construct2_24 off-target 1	TGCTCCGATTCC**GAGGATGGCGCCGGCGACAA**TGGCGAAGCC
Construct2_25 off-target 1	TGCTCCGATTCC**GAGGATGGCGCCGGCGACAA**TGGCGAAGCC
Construct2_26 off-target 1	TGCTCCGATTCC**GAGGATGGCGCCGGCGACAA**TGGCAAAGCC
Construct2_27 off-target 1	TGCTCCGATTCC**GAGGATGGCGCCGGCGACAA**TGGCAAAGCC
Construct2_28 off-target 1	TGCTCCGATTCC**GAGGATGGCGCCGGCGACAA**TGGCGAAGCC
Construct2_29 off-target 1	TGCTCCGATTCC**GAGGATGGCGCCGGCGACAA**TGGCAAAGCC
Construct2_30 off-target 1	TGCTCCGATTCC**GAGGATGGCGCCGGCGACAA**TGGCAAAGCC
Construct2_31 off-target 1	TGCTCCGATTCC**GAGGATGGCGCCGGCGACAA**TGGCGAAGCC
Construct2_32 off-target 1	TGCTCCGATTCC**GAGGATGGCGCCGGCGACAA**TGGCGAAGCC
Construct2_33 off-target 1	TGCTCCGATTCC**GAGGATGGCGCCGGCGACAA**TGGCAAAGCC
Construct2_34 off-target 1	TGCTCCGATTCC**GAGGATGGCGCCGGCGACAA**TGGCAAAGCC
Construct2_35 off-target 1	TGCTCCGATTCC**GAGGATGGCGCCGGCGACAA**TGGCAAAGCC
Construct2_36 off-target 1	TGCTCCGATTCC**GAGGATGGCGCCGGCGACAA**TGGCAAAGCC
Construct2_37 off-target 1	TGCTCCGATTCC**GAGGATGGCGCCGGCGACAA**TGGCAAAGCC
Construct2_38 off-target 1	TGCTCCGATTCC**GAGGATGGCGCCGGCGACAA**TGGCAAAGCC
Construct2_39 off-target 1	TGCTCCGATTCC**GAGGATGGCGCCGGCGACAA**TGGCGAAGCC
Construct2_40 off-target 1	TGCTCCGATTCC**GAGGATGGCGCCGGCGACAA**TGGCGAAGCC
Construct2_41 off-target 1	TGCTCCGATTCC**GAGGATGGCGCCGGCGACAA**TGGCAAAGCC
Construct2_42 off-target 1	TGCTCCGATTCC**GAGGATGGCGCCGGCGACAA**TGGCGAAGCC
Construct2_43 off-target 1	TGCTCCGATTCC**GAGGATGGCGCCGGCGACAA**TGGCGAAGCC
Construct2_44 off-target 1	TGCTCCGATTCC**GAGGATGGCGCCGGCGACAA**TGGCGAAGCC
Construct2_45 off-target 1	TGCTCCGATTCC**GAGGATGGCGCCGGCGACAA**TGGCAAAGCC
Construct2_46 off-target 1	TGCTCCGATTCC**GAGGATGGCGCCGGCGACAA**TGGCGAAGCC
Construct2_47 off-target 1	TGCTCCGATTCC**GAGGATGGCGCCGGCGACAA**TGGCGAAGCC
Construct2_48 off-target 1	TGCTCCGATTCC**GAGGATGGCGCCGGCGACAA**TGGCAAAGCC
Construct2_49 off-target 1	TGCTCCGATTCC**GAGGATGGCGCCGGCGACAA**TGGCAAAGCC
Construct2_50 off-target 1	TGCTCCGATTCC**GAGGATGGCGCCGGCGACAA**TGGCGAAGCC
Construct2_51 off-target 1	TGCTCCGATTCC**GAGGATGGCGCCGGCGACAA**TGGCGAAGCC
Construct2_52 off-target 1	TGCTCCGATTCC**GAGGATGGCGCCGGCGACAA**TGGCAAAGCC
Construct2_53 off-target 1	TGCTCCGATTCC**GAGGATGGCGCCGGCGACAA**TGGCGAAGCC
Construct2_54 off-target 1	TGCTCCGATTCC**GAGGATGGCGCCGGCGACAA**TGGCAAAGCC
Construct2_55 off-target 1	TGCTCCGATTCC**GAGGATGGCGCCGGCGACAA**TGGCGAAGCC
Construct2_56 off-target 1	TGCTCCGATTCC**GAGGATGGCGCCGGCGACAA**TGGCAAAGCC
Construct2_57 off-target 1	TGCTCCGATTCC**GAGGATGGCGCCGGCGACAA**TGGCAAAGCC
Construct2_58 off-target 1	TGCTCCGATTCC**GAGGATGGCGCCGGCGACAA**TGGCAAAGCC
Construct2_59 off-target 1	TGCTCCGATTCC**GAGGATGGCGCCGGCGACAA**TGGCAAAGCC
Construct2_60 off-target 1	TGCTCCGATTCC**GAGGATGGCGCCGGCGACAA**TGGCAAAGCC
Construct2_61 off-target 1	TGCTCCGATTCC**GAGGATGGCGCCGGCGACAA**TGGCAAAGCC
Construct2_62 off-target 1	TGCTCCGATTCC**GAGGATGGCGCCGGCGACAA**TGGCAAAGCC
Construct2_63 off-target 1	TGCTCCGATTCC**GAGGATGGCGCCGGCGACAA**TGGCGAAGCC
Construct2_64 off-target 1	TGCTCCGATTCC**GAGGATGGCGCCGGCGACAA**TGGCGAAGCC
Construct2_65 off-target 1	TGCTCCGATTCC**GAGGATGGCGCCGGCGACAA**TGGCGAAGCC
Construct2_66 off-target 1	TGCTCCGATTCC**GAGGATGGCGCCGGCGACAA**TGGCGAAGCC
Construct2_67 off-target 1	TGCTCCGATTCC**GAGGATGGCGCCGGCGACAA**TGGCAAAGCC
Construct2_68 off-target 1	TGCTCCGATTCC**GAGGATGGCGCCGGCGACAA**TGGCAAAGCC
Construct2_69 off-target 1	TGCTCCGATTCC**GAGGATGGCGCCGGCGACAA**TGGCAAAGCC
Construct2_70 off-target 1	TGCTCCGATTCC**GAGGATGGCGCCGGCGACAA**TGGCGAAGCC
Construct2_71 off-target 1	TGCTCCGATTCC**GAGGATGGCGCCGGCGACAA**TGGCAAAGCC
Construct2_72 off-target 1	TGCTCCGATTCC**GAGGATGGCGCCGGCGACAA**TGGCGAAGCC
Construct2_73 off-target 1	TGCTCCGATTCC**GAGGATGGCGCCGGCGACAA**TGGCAAAGCC
Construct2_74 off-target 1	TGCTCCGATTCC**GAGGATGGCGCCGGCGACAA**TGGCAAAGCC
Construct2_75 off-target 1	TGCTCCGATTCC**GAGGATGGCGCCGGCGACAA**TGGCAAAGCC
Construct2_76 off-target 1	TGCTCCGATTCC**GAGGATGGCGCCGGCGACAA**TGGCAAAGCC
Construct2_77 off-target 1	TGCTCCGATTCC**GAGGATGGCGCCGGCGACAA**TGGCAAAGCC
Construct2_78 off-target 1	TGCTCCGATTCC**GAGGATGGCGCCGGCGACAA**TGGCAAAGCC
Construct2_79 off-target 1	TGCTCCGATTCC**GAGGATGGCGCCGGCGACAA**TGGCAAAGCC
Construct2_80 off-target 1	TGCTCCGATTCC**GAGGATGGCGCCGGCGACAA**TGGCAAAGCC
Construct2_81 off-target 1	TGCTCCGATTCC**GAGGATGGCGCCGGCGACAA**TGGCGAAGCC
Construct2_82 off-target 1	TGCTCCGATTCC**GAGGATGGCGCCGGCGACAA**TGGCAAAGCC
Construct2_83 off-target 1	TGCTCCGATTCC**GAGGATGGCGCCGGCGACAA**TGGCAAAGCC
Construct2_84 off-target 1	TGCTCCGATTCC**GAGGATGGCGCCGGCGACAA**TGGCAAAGCC
Construct2_85 off-target 1	TGCTCCGATTCC**GAGGATGGCGCCGGCGACAA**TGGCAAAGCC
Construct2_86 off-target 1	TGCTCCGATTCC**GAGGATGGCGCCGGCGACAA**TGGCAAAGCC
Construct2_87 off-target 1	TGCTCCGATTCC**GAGGATGGCGCCGGCGACAA**TGGCAAAGCC
Construct2_88 off-target 1	TGCTCCGATTCC**GAGGATGGCGCCGGCGACAA**TGGCAAAGCC
Construct2_89 off-target 1	TGCTCCGATTCC**GAGGATGGCGCCGGCGACAA**TGGCAAAGCC
Construct2_90 off-target 1	TGCTCCGATTCC**GAGGATGGCGCCGGCGACAA**TGGCAAAGCC
Construct2_91 off-target 1	TGCTCCGATTCC**GAGGATGGCGCCGGCGACAA**TGGCAAAGCC
Construct2_92 off-target 1	TGCTCCGATTCC**GAGGATGGCGCCGGCGACAA**TGGCAAAGCC
Construct2_93 off-target 1	TGCTCCGATTCC**GAGGATGGCGCCGGCGACAA**TGGCAAAGCC
Construct2_94 off-target 1	TGCTCCGATTCC**GAGGATGGCGCCGGCGACAA**TGGCAAAGCC
Construct2_95 off-target 1	TGCTCCGATTCC**GAGGATGGCGCCGGCGACAA**TGGCAAAGCC
Construct2_96 off-target 1	TGCTCCGATTCC**GAGGATGGCGCCGGCGACAA**TGGCAAAGCC
Construct2_97 off-target 1	TGCTCCGATTCC**GAGGATGGCGCCGGCGACAA**TGGCAAAGCC
	************************************ *****

WILD-TYPE off-target 3	AACAGTGTGC**AGGAAGCCTTAGCTGATCCA**AGGTGGAAAGCAGCCA
Construct2_1 off-target 3	AACAGTGTGC**AGGAAGCCTTAGCTGATCCA**AGGTGGAAAGCAGCCA
Construct2_2 off-target 3	AACAGTGTGC**AGGAAGCCTTAGCTGATCCA**AGGTGGAAAGCAGCCA
Construct2_3 off-target 3	AACAGTGTGC**AGGAAGCCTTAGCTGATCCA**AGGTGGAAAGCAGCCA
Construct2_4 off-target 3	AACAGTGTGC**AGGAAGCCTTAGCTGATCCA**AGGTGGAAAGCAGCCA
Construct2_5 off-target 3	AACAGTGTGC**AGGAAGCCTTAGCTGATCCA**AGGTGGAAAGCAGCCA
Construct2_6 off-target 3	AACAGTGTGC**AGGAAGCCTTAGCTGATCCA**AGGTGGAAAGCAGCCA
Construct2_7 off-target 3	AACAGTGTGC**AGGAAGCCTTAGCTGATCCA**AGGTGGAAAGCAGCCA
Construct2_8 off-target 3	AACAGTGTGC**AGGAAGCCTTAGCTGATCCA**AGGTGGAAAGCAGCCA
Construct2_9 off-target 3	AACAGTGTGC**AGGAAGCCTTAGCTGATCCA**AGGTGGAAAGCAGCCA
Construct2_10 off-target 3	AACAGTGTGC**AGGAAGCCTTAGCTGATCCA**AGGTGGAAAGCAGCCA
Construct2_11 off-target 3	AACAGTGTGC**AGGAAGCCTTAGCTGATCCA**AGGTGGAAAGCAGCCA
Construct2_12 off-target 3	AACAGTGTGC**AGGAAGCCTTAGCTGATCCA**AGGTGGAAAGCAGCCA
Construct2_13 off-target 3	AACAGTGTGC**AGGAAGCCTTAGCTGATCCA**AGGTGGAAAGCAGCCA
Construct2_14 off-target 3	AACAGTGTGC**AGGAAGCCTTAGCTGATCCA**AGGTGGAAAGCAGCCA
Construct2_15 off-target 3	AACAGTGTGC**AGGAAGCCTTAGCTGATCCA**AGGTGGAAAGCAGCCA
Construct2_16 off-target 3	AACAGTGTGC**AGGAAGCCTTAGCTGATCCA**AGGTGGAAAGCAGCCA
Construct2_17 off-target 3	AACAGTGTGC**AGGAAGCCTTAGCTGATCCA**AGGTGGAAAGCAGCCA
Construct2_18 off-target 3	AACAGTGTGC**AGGAAGCCTTAGCTGATCCA**AGGTGGAAAGCAGCCA
Construct2_19 off-target 3	AACAGTGTGC**AGGAAGCCTTAGCTGATCCA**AGGTGGAAAGCAGCCA
Construct2_20 off-target 3	AACAGTGTGC**AGGAAGCCTTAGCTGATCCA**AGGTGGAAAGCAGCCA
Construct2_21 off-target 3	AACAGTGTGC**AGGAAGCCTTAGCTGATCCA**AGGTGGAAAGCAGCCA
Construct2_22 off-target 3	AACAGTGTGC**AGGAAGCCTTAGCTGATCCA**AGGTGGAAAGCAGCCA
Construct2_23 off-target 3	AACAGTGTGC**AGGAAGCCTTAGCTGATCCA**AGGTGGAAAGCAGCCA
Construct2_24 off-target 3	AACAGTGTGC**AGGAAGCCTTAGCTGATCCA**AGGTGGAAAGCAGCCA
Construct2_25 off-target 3	AACAGTGTGC**AGGAAGCCTTAGCTGATCCA**AGGTGGAAAGCAGCCA
Construct2_26 off-target 3	AACAGTGTGC**AGGAAGCCTTAGCTGATCCA**AGGTGGAAAGCAGCCA
Construct2_27 off-target 3	AACAGTGTGC**AGGAAGCCTTAGCTGATCCA**AGGTGGAAAGCAGCCA
Construct2_28 off-target 3	AACAGTGTGC**AGGAAGCCTTAGCTGATCCA**AGGTGGAAAGCAGCCA
Construct2_29 off-target 3	AACAGTGTGC**AGGAAGCCTTAGCTGATCCA**AGGTGGAAAGCAGCCA
Construct2_30 off-target 3	AACAGTGTGC**AGGAAGCCTTAGCTGATCCA**AGGTGGAAAGCAGCCA
Construct2_31 off-target 3	AACAGTGTGC**AGGAAGCCTTAGCTGATCCA**AGGTGGAAAGCAGCCA
Construct2_32 off-target 3	AACAGTGTGC**AGGAAGCCTTAGCTGATCCA**AGGTGGAAAGCAGCCA
Construct2_33 off-target 3	AACAGTGTGC**AGGAAGCCTTAGCTGATCCA**AGGTGGAAAGCAGCCA
Construct2_34 off-target 3	AACAGTGTGC**AGGAAGCCTTAGCTGATCCA**AGGTGGAAAGCAGCCA
Construct2_35 off-target 3	AACAGTGTGC**AGGAAGCCTTAGCTGATCCA**AGGTGGAAAGCAGCCA
Construct2_36 off-target 3	AACAGTGTGC**AGGAAGCCTTAGCTGATCCA**AGGTGGAAAGCAGCCA
Construct2_37 off-target 3	AACAGTGTGC**AGGAAGCCTTAGCTGATCCA**AGGTGGAAAGCAGCCA
Construct2_38 off-target 3	AACAGTGTGC**AGGAAGCCTTAGCTGATCCA**AGGTGGAAAGCAGCCA
Construct2_39 off-target 3	AACAGTGTGC**AGGAAGCCTTAGCTGATCCA**AGGTGGAAAGCAGCCA
Construct2_40 off-target 3	AACAGTGTGC**AGGAAGCCTTAGCTGATCCA**AGGTGGAAAGCAGCCA
Construct2_41 off-target 3	AACAGTGTGC**AGGAAGCCTTAGCTGATCCA**AGGTGGAAAGCAGCCA
Construct2_42 off-target 3	AACAGTGTGC**AGGAAGCCTTAGCTGATCCA**AGGTGGAAAGCAGCCA
Construct2_43 off-target 3	AACAGTGTGC**AGGAAGCCTTAGCTGATCCA**AGGTGGAAAGCAGCCA
Construct2_44 off-target 3	AACAGTGTGC**AGGAAGCCTTAGCTGATCCA**AGGTGGAAAGCAGCCA
Construct2_45 off-target 3	AACAGTGTGC**AGGAAGCCTTAGCTGATCCA**AGGTGGAAAGCAGCCA
Construct2_46 off-target 3	AACAGTGTGC**AGGAAGCCTTAGCTGATCCA**AGGTGGAAAGCAGCCA
Construct2_47 off-target 3	AACAGTGTGC**AGGAAGCCTTAGCTGATCCA**AGGTGGAAAGCAGCCA
Construct2_48 off-target 3	AACAGTGTGC**AGGAAGCCTTAGCTGATCCA**AGGTGGAAAGCAGCCA
Construct2_49 off-target 3	AACAGTGTGC**AGGAAGCCTTAGCTGATCCA**AGGTGGAAAGCAGCCA
Construct2_50 off-target 3	AACAGTGTGC**AGGAAGCCTTAGCTGATCCA**AGGTGGAAAGCAGCCA
Construct2_51 off-target 3	AACAGTGTGC**AGGAAGCCTTAGCTGATCCA**AGGTGGAAAGCAGCCA
Construct2_52 off-target 3	AACAGTGTGC**AGGAAGCCTTAGCTGATCCA**AGGTGGAAAGCAGCCA
Construct2_53 off-target 3	AACAGTGTGC**AGGAAGCCTTAGCTGATCCA**AGGTGGAAAGCAGCCA
Construct2_54 off-target 3	AACAGTGTGC**AGGAAGCCTTAGCTGATCCA**AGGTGGAAAGCAGCCA
Construct2_55 off-target 3	AACAGTGTGC**AGGAAGCCTTAGCTGATCCA**AGGTGGAAAGCAGCCA
Construct2_56 off-target 3	AACAGTGTGC**AGGAAGCCTTAGCTGATCCA**AGGTGGAAAGCAGCCA
Construct2_57 off-target 3	AACAGTGTGC**AGGAAGCCTTAGCTGATCCA**AGGTGGAAAGCAGCCA
Construct2_58 off-target 3	AACAGTGTGC**AGGAAGCCTTAGCTGATCCA**AGGTGGAAAGCAGCCA
Construct2_59 off-target 3	AACAGTGTGC**AGGAAGCCTTAGCTGATCCA**AGGTGGAAAGCAGCCA
Construct2_60 off-target 3	AACAGTGTGC**AGGAAGCCTTAGCTGATCCA**AGGTGGAAAGCAGCCA
Construct2_61 off-target 3	AACAGTGTGC**AGGAAGCCTTAGCTGATCCA**AGGTGGAAAGCAGCCA
Construct2_62 off-target 3	AACAGTGTGC**AGGAAGCCTTAGCTGATCCA**AGGTGGAAAGCAGCCA
Construct2_63 off-target 3	AACAGTGTGC**AGGAAGCCTTAGCTGATCCA**AGGTGGAAAGCAGCCA
Construct2_64 off-target 3	AACAGTGTGC**AGGAAGCCTTAGCTGATCCA**AGGTGGAAAGCAGCCA
Construct2_65 off-target 3	AACAGTGTGC**AGGAAGCCTTAGCTGATCCA**AGGTGGAAAGCAGCCA
Construct2_66 off-target 3	AACAGTGTGC**AGGAAGCCTTAGCTGATCCA**AGGTGGAAAGCAGCCA
Construct2_67 off-target 3	AACAGTGTGC**AGGAAGCCTTAGCTGATCCA**AGGTGGAAAGCAGCCA
Construct2_68 off-target 3	AACAGTGTGC**AGGAAGCCTTAGCTGATCCA**AGGTGGAAAGCAGCCA
Construct2_69 off-target 3	AACAGTGTGC**AGGAAGCCTTAGCTGATCCA**AGGTGGAAAGCAGCCA
Construct2_70 off-target 3	AACAGTGTGC**AGGAAGCCTTAGCTGATCCA**AGGTGGAAAGCAGCCA
Construct2_71 off-target 3	AACAGTGTGC**AGGAAGCCTTAGCTGATCCA**AGGTGGAAAGCAGCCA
Construct2_72 off-target 3	AACAGTGTGC**AGGAAGCCTTAGCTGATCCA**AGGTGGAAAGCAGCCA
Construct2_73 off-target 3	AACAGTGTGC**AGGAAGCCTTAGCTGATCCA**AGGTGGAAAGCAGCCA
Construct2_74 off-target 3	AACAGTGTGC**AGGAAGCCTTAGCTGATCCA**AGGTGGAAAGCAGCCA
Construct2_75 off-target 3	AACAGTGTGC**AGGAAGCCTTAGCTGATCCA**AGGTGGAAAGCAGCCA
Construct2_76 off-target 3	AACAGTGTGC**AGGAAGCCTTAGCTGATCCA**AGGTGGAAAGCAGCCA
Construct2_77 off-target 3	AACAGTGTGC**AGGAAGCCTTAGCTGATCCA**AGGTGGAAAGCAGCCA
Construct2_78 off-target 3	AACAGTGTGC**AGGAAGCCTTAGCTGATCCA**AGGTGGAAAGCAGCCA
Construct2_79 off-target 3	AACAGTGTGC**AGGAAGCCTTAGCTGATCCA**AGGTGGAAAGCAGCCA
Construct2_80 off-target 3	AACAGTGTGC**AGGAAGCCTTAGCTGATCCA**AGGTGGAAAGCAGCCA
Construct2_81 off-target 3	AACAGTGTGC**AGGAAGCCTTAGCTGATCCA**AGGTGGAAAGCAGCCA
Construct2_82 off-target 3	AACAGTGTGC**AGGAAGCCTTAGCTGATCCA**AGGTGGAAAGCAGCCA
Construct2_83 off-target 3	AACAGTGTGC**AGGAAGCCTTAGCTGATCCA**AGGTGGAAAGCAGCCA
Construct2_84 off-target 3	AACAGTGTGC**AGGAAGCCTTAGCTGATCCA**AGGTGGAAAGCAGCCA
Construct2_85 off-target 3	AACAGTGTGC**AGGAAGCCTTAGCTGATCCA**AGGTGGAAAGCAGCCA
Construct2_86 off-target 3	AACAGTGTGC**AGGAAGCCTTAGCTGATCCA**AGGTGGAAAGCAGCCA
Construct2_87 off-target 3	AACAGTGTGC**AGGAAGCCTTAGCTGATCCA**AGGTGGAAAGCAGCCA
Construct2_88 off-target 3	AACAGTGTGC**AGGAAGCCTTAGCTGATCCA**AGGTGGAAAGCAGCCA
Construct2_89 off-target 3	AACAGTGTGC**AGGAAGCCTTAGCTGATCCA**AGGTGGAAAGCAGCCA
Construct2_90 off-target 3	AACAGTGTGC**AGGAAGCCTTAGCTGATCCA**AGGTGGAAAGCAGCCA
Construct2_91 off-target 3	AACAGTGTGC**AGGAAGCCTTAGCTGATCCA**AGGTGGAAAGCAGCCA
Construct2_92 off-target 3	AACAGTGTGC**AGGAAGCCTTAGCTGATCCA**AGGTGGAAAGCAGCCA
Construct2_93 off-target 3	AACAGTGTGC**AGGAAGCCTTAGCTGATCCA**AGGTGGAAAGCAGCCA
Construct2_94 off-target 3	AACAGTGTGC**AGGAAGCCTTAGCTGATCCA**AGGTGGAAAGCAGCCA
Construct2_95 off-target 3	AACAGTGTGC**AGGAAGCCTTAGCTGATCCA**AGGTGGAAAGCAGCCA
Construct2_96 off-target 3	AACAGTGTGC**AGGAAGCCTTAGCTGATCCA**AGGTGGAAAGCAGCCA
Construct2_97 off-target 3	AACAGTGTGC**AGGAAGCCTTAGCTGATCCA**AGGTGGAAAGCAGCCA
	**

Figure S10. Alignment of sequenced Off-target1 and off-target3 sites from 97 independent transgenic lines expressing construct 2 (*p35S::zCas9i*). Wild-type sequence is highlighted in grey. In the off-target 1 sequence TGGCA(A/G)AAGCC, the base A or G in (A/G) is normally found as an heterozygous nucleotide in wild-type.

primer name	5' -> 3' sequence
VvLYK6_cDNA_Full_F	ATGAAGCTCAAACCCCTAAACGA
VvLYK6_cDNA_Full_R	CTATCTAGCTATAACTGCAGCGGG
hCas9_genotyping_FP	GCCTGTTTGGTAATCTTATCGC
hCas9_genotyping_RP	TCTTTCCACTCTGCTTGTCTCG
Zcas9i_genotyping_FP	TTGGCACATACCATGACCTG
Zcas9i_genotyping_RP	ATCTGACTCCCAAGCTCCTT
VvLYK6_genotyping_FP	GAGCCCAGCAACATCTCCTC
VvLYK6_genotyping_RP	CAGCTCTTCATAGGCATTCCACT
hCas9_QPCR_FP2	AGACCGAAGTACAGACCGGA
hCas9_QPCR_RP2	ATCCGCCGTATTTCTTGGGG
zCas9i_QPCR_FP2	GCCAAGGCAATACTGTCTGC
zCas9i_QPCR_FP2	CCCAGAGACAGTGCAATCAGA
hcas9_Quant_FP	ATTCGGAAGCGACCACTTATC
hcas9_Quant_RP	CAGTTCCTTGACGCTTTTGAG
zCas9i_Quant_FP	GCTCGTCCAAACCTACAATCA
zCas9i_Quant_RP	GCGAGGTCAAAGTTGCTTTT
VvEF1alpha-QPCR_FP	TCTGCCTTCTTCCTTGGGTA
VvEF1alpha-QPCR_RP	GCACCTCGATCAAAAGAGGA
VvEF1alpha-Quant_FP	CTGTCATAGATCGTCCGCTTT
VvEF1alpha-Quant_RP	TCCGGAGTAAAAGACACAACAA
VATP16_QPCR_FP	GTTTTTCGGATTCCTCGGCG
VATP16_QPCR_RP	CGAGAGATGCGCATAGCCA
VATP16_Quant_FP	CGGCACCGTTTTTCGGATTC
VATP16_Quant_RP	ATGCGCATAGCCATCGAAAAG
Off-target1_genotyping_FP	AGCAGCATCTTCCAATTTCGG
Off-target1_genotyping_RP	CTTGGGTGGTGGCATCTACA
Off-target2_genotyping_FP	GTGGTCAACAATTTGGGTCCG
Off-target2_genotyping_RP	ATGCTCTCGGGGATTGCTTC

Table S1. Primers used in this study.

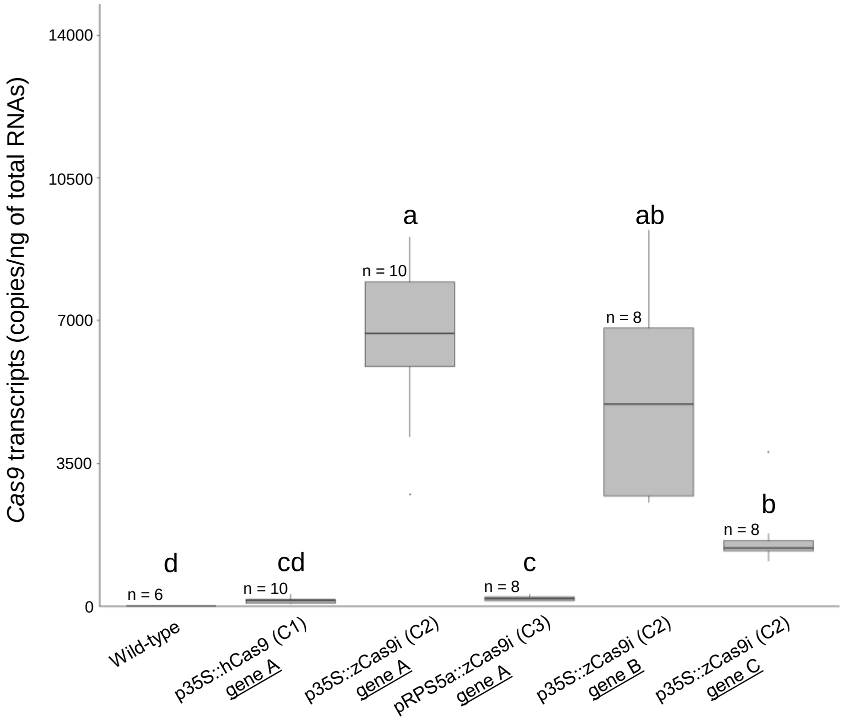

Figure S11. Cas9 transcripts in stable transformed grapevine plantlets including transgenic lines expressing *p35S::zCas9i* from other transformation events to target different genes. *Cas9* transcripts levels from leaves collected on grapevine plantlets. Lowercase letters indicate significant differences between samples (Kruskal Wallis, $p<0.001$). Target genes correspond to gene A = Vitvi05g00623, gene B = Vitvi04g01216, gene C = Vitvi04g01214. For experiment, "n" represents the number of biological replicates. Each independent biological replicate involved sampling three leaves from three distinct plantlets. Different plantlets were sampled for each independent biological replicate. All the plantlets sampled correspond to those sequenced in Figure 4.

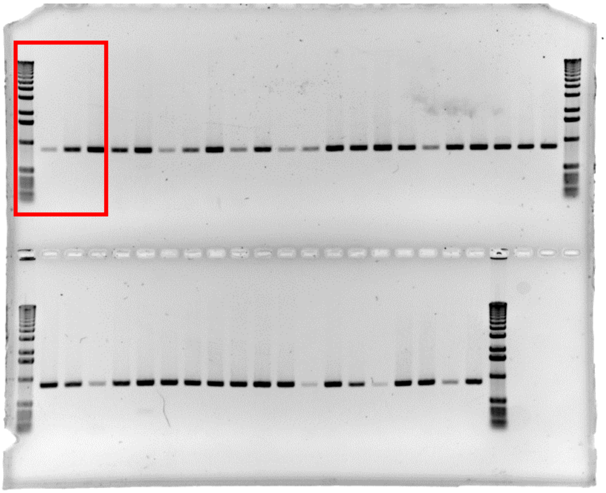

Figure S12. Original agarose gel illustrated Figure 2h. The surrounded area in red is the part illustrated figure 2h.

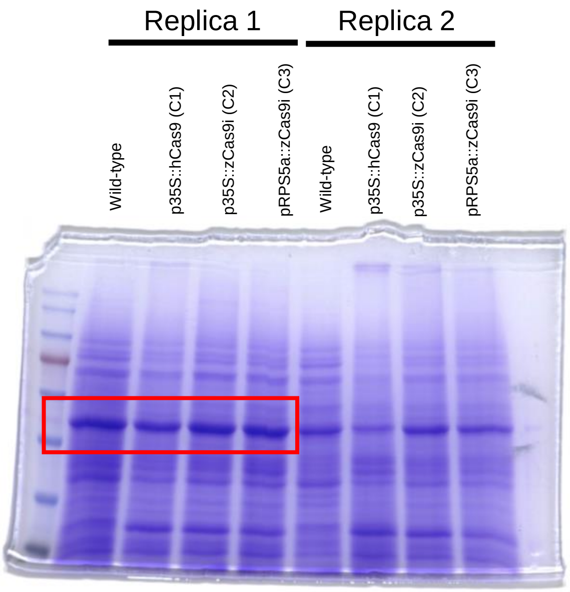

Figure S13. Original Coomassie blue gel illustrated figure 5b. Coomassie blue gel with surrounded area in red corresponding to Coomassie blue picture illustrated figure 5b.

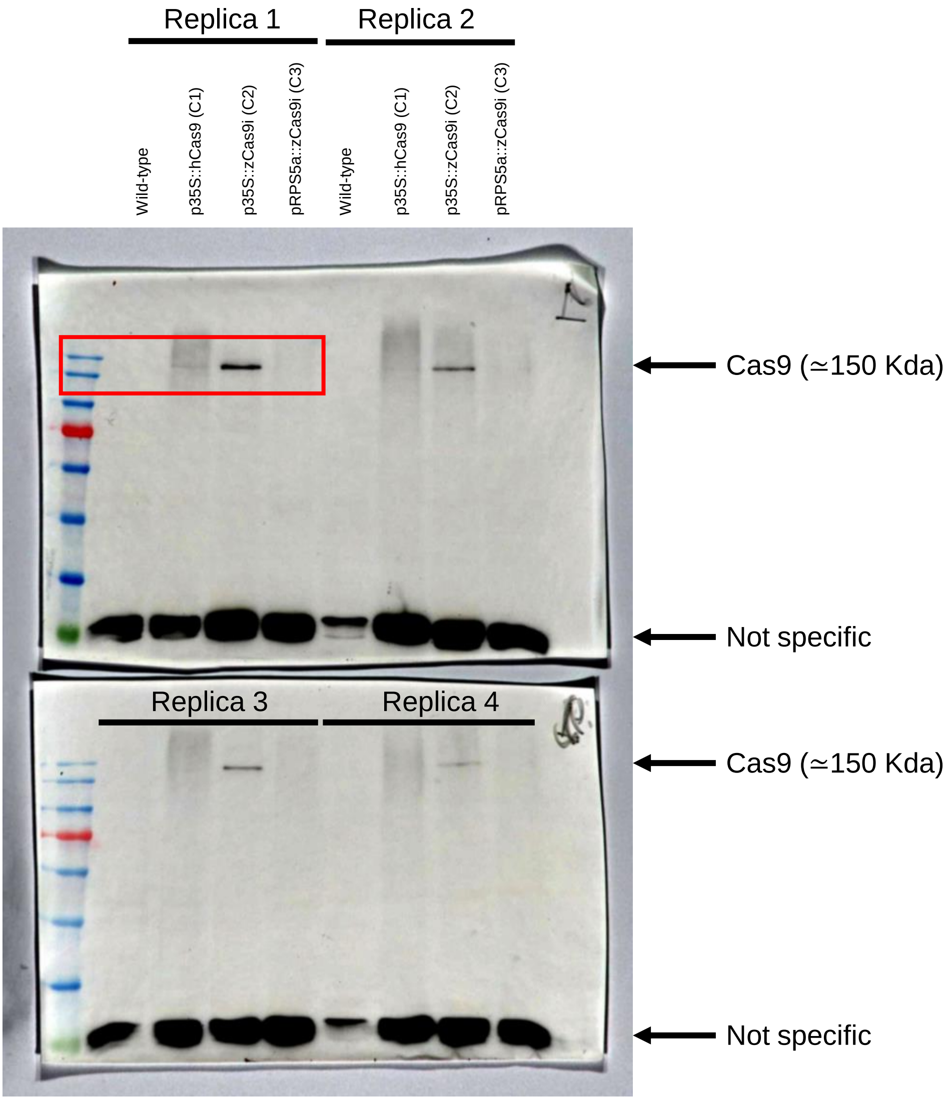

Figure S14. Original westernblotting with Cas9 antibody illustrated figure 5b. The surrounded area in red is the part illustrated figure 5b.
